# Synthesis and Insecticidal/Fungicidal Activities of Triazone Derivatives Containing Acylhydrazone Moieties

**DOI:** 10.3390/molecules30020340

**Published:** 2025-01-16

**Authors:** Peipei Cui, Yan Yang

**Affiliations:** 1 College of Architecture and Arts, Taiyuan University of Technology, Jinzhong 030060, China; cui_peipei@163.com; 2 College of Chemistry and Chemical Engineering, Taiyuan University of Technology, Taiyuan 030024, China

**Keywords:** triazone, hydrazone, biological activities

## Abstract

A series of novel triazone derivatives containing aldehyde hydrazone or ketone hydrazone moieties were designed, synthesized and their biological activities were investigated against *Aphis craccivora*, *Culex pipiens pallens*, *Helicoverpa armigera*, *Ostrinia nubilalis*, *Mythimna separata* and 14 Kinds of fungi. Most of the aldehyde hydrazone exhibited excellent insecticidal activities against *A. craccivora*. In particular, the aphicidal activities of compounds **3t** (35%) and **3w** (30%) were equivalent to pymetrozine (30%) at 5 mg/kg. The aphicidal activities of derivatives **3p**, **3u**, **3y**, **5g**, **5i**, **5l**, **5q** and **5u** against *C. pipiens pallens* were higher than that of pymetrozine. Compound **3u** (100%) exhibited good larvicidal activities against *C. pipiens pallens* at 0.25 mg/kg. Most derivatives exhibited broad-spectrum fungicidal activities against 14 kinds of plant fungi at 50 mg/kg. Thirty-nine compounds exhibited a more than 50% inhibition rate against *Physalospora piricola*. Compounds **3h**, **3t** and **3w** were expected to be the leading structure for the development of new triazone insecticides agents.

## 1. Introduction

Owing to their direct feeding, transmission of destructive plant viruses and high reproductive rate [1], aphids (Hemiptera: *Aphididae*) have become the most destructive pests, causing plant diseases that significantly reduce crop yield and quality [2].

**Figure 1:**
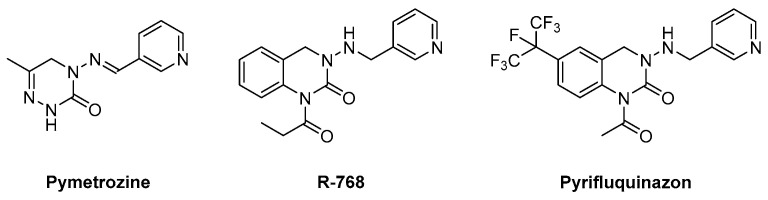
Pymetrozine, R-768 and pyrifluquinazon.

As a pyridine azomethine insecticide, pymetrozine was first developed by Syngenta in 1988 [3] and has become a hot research topic globally due to its high efficiency, satisfied selectivity and environmental friendliness [4,5,6,7,8,9,10,11,12,13,14]. However, only two analogs of this type of insecticides (R-768 and pyrifluquinazon (Figure 1)) have been found so far, and their activities were significantly lower than that of pymetrozine [9,15].

**Figure 2:**
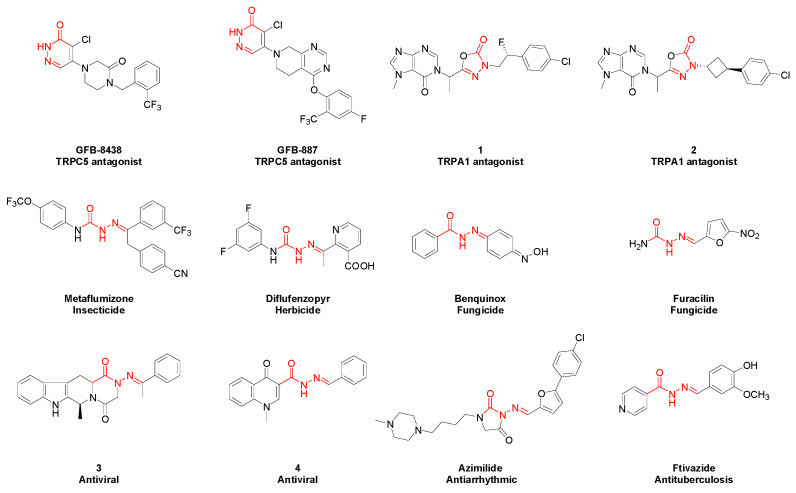
Selected TRP antagonists, pesticides and drugs containing hydrazones moieties. The hydrazone structure is marked with different colors. For compounds with proprietary names, proprietary names are used. For compounds without proprietary names, numbers are used.

In 2015, Nesterov et al. found that the action targets of pymetrozine insecticide were transient receptor potential (TRP) channels [16], which were non-selective cation channels composed of 28 cation-permeable channels. TRPC (transient receptor potential-canonical) was a subfamily of TRP channels [17], which was inwardly rectifying and was considered as a therapeutic target for mental disorders such as depression, borderline personality disorder and post-traumatic stress disorder [18,19]. The pyridazinone compound GFB-8438 (Figure 2) was a highly selective, potent TRPC5 antagonist, which specifically interacted with the TRPC5-Rac1 pathway in podocytes to attenuate FSGS and diabetic nephropathy [20]. Pymetrozine acted on the same target site as GFB-8438 [16].

**Figure 3:**
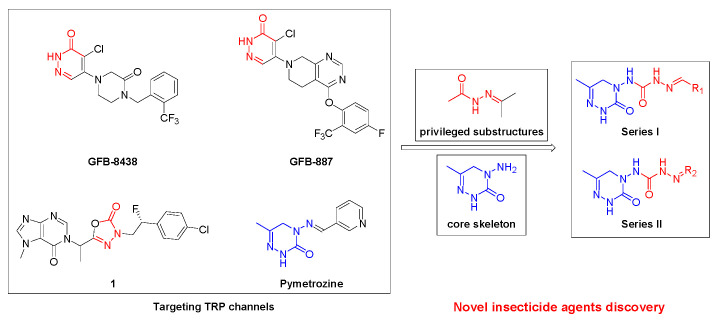
Design of target compounds. The hydrazone structure is marked with different colors. For compounds with proprietary names, proprietary names are used. For compounds without proprietary names, numbers are used.

Hydrazones moieties are important structural units that exist in many bioactive molecules, with a wide range of biological activities. Metaflumizone is a semicarbazone insecticide developed by BASF (Ludwigshafen, Germany) and Nihon Nohyaku Co., Ltd. (Tokyo, Japan) which provides good-to-excellent control of lepidopterous pests and certain pests in the orders Coleoptera, Hemiptera, Hymenoptera, Diptera, Isoptera and Siphonaptera [21]. Diflufenzopyr containing hydrazone moiety is also used as a commercialized insecticide [22]. Benquinox is a desirable fungicide which protects seeds and seedlings against fungal infections [23]. Furacilin has been used for the treatment of bacterial and protozoal infections for many years [24]. Compounds **3** [25] and **4** [26] exhibit higher antiviral activities than ribavirin. Azimilide and Ftivazide exhibit antiarrhythmic [27] and antituberculosis [28] activities, respectively. GFB-887 [29] compounds **1** and **2** [30] (Figure 2) containing hydrazone moieties were used as TRP antagonists. Based on the bioactivies and targeting TRP channels of GFB-8438, GFB-887, 1 and pymetrozine, the acylhydrazone structure was introduced into pymetrozine to design and synthesize a series of triazone aldehyde and ketone hydrazone derivatives (Figure 3). Their insecticidal activities against bean aphid and lepidopteran pests were repeated three times. In addition, their antiphytopathogenic fungus activities were also investigated.

## 2. Results and Discussions

### 2.1. General Synthesis

**Scheme 1:**
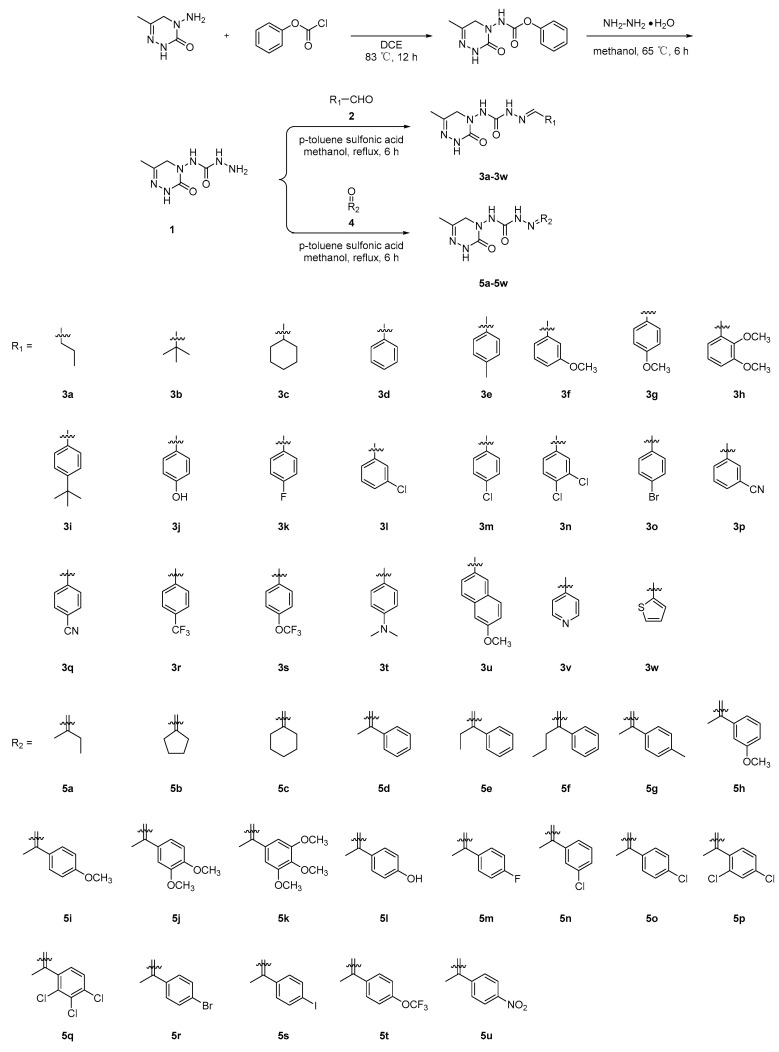
Synthetic Route for **3a**–**3w** and **5a**–**5u**.

The synthetic routes of the target compounds **3a**–**3w** and **5a**–**5u** are given in Scheme 1. Intermediate 1 was synthesized by the method reported in the literature [31]. 4-Amino-6-methyl-4,5-dihydro-2H-[1,2,4]triazin-3-one reacted with benzoyl chloride to give (6-Methyl-3-oxo-2,5-dihydro-3H-[1,2,4]triazin-4-yl)-carbamic acid phenyl ester, which reacted with hydrazine hydrate to give intermediate 1. Using p-toluenesulfonic acid as a catalyst, compound **1** reacted with aldehydes **2a**–**2w** to form compounds **3a**–**3w** in 57–88% yields. Compounds **5a**–**5u** were synthesized by the condensation of compound **1** and ketones **4a**–**4u** in 56–97% yields. Alkyl, cycloalkyl, and substituted phenyl were introduced into acylhydrazone derivatives to investigate the effect of different substituents on activity. Experimental operating steps and data of target compounds **3a**–**3w** and **5a**–**5u** are available in the Supplementary Materials.

### 2.2. Biological Evaluation

#### 2.2.1. Foliar Contact Activity Against Bean Aphid (*A. craccivora*)

As seen from Table 1, most aldehyde hydrazone compounds exhibited weak insecticidal activities; only compounds **3h** (2, 3-dimethoxy, 15%), **3t** (4-dimethylamino, 35%) and **3w** (2-thiophene, 30%) exhibited the same level of aphicidal activities as pymetrozine (30%) at 5 mg/kg. Compounds **3t** and **3w** exhibited high insecticidal activities because nitrogen and sulfur atoms may form hydrogen bonding with the TRP channels [16]. The rotation of chemical bonds of acylhydrazone compounds can lead to different conformations [32], also affecting their biological activities.

When R was an alkyl substituent, the size of the substituent had significant effects on the aphicidal activities (**3a**–**3c**). For example, the insecticidal activity of compound **3b** (85%, t-butyl) was higher than that of compound **3a** (30%, n-propyl) at 100 mg/kg. When R was aromatic groups, the activities increased obviously. In order to investigate the effects of the substituent positions, electrical properties and the number of substituents on the phenyl group on the insecticidal activities, different substituents were introduced to the benzene ring (**3e**–**3t**). The results indicated that most of these derivatives exhibited more than 30% activity at 10 mg/kg, which was lower than that of pymetrozine (90%). The positions and numbers of electron-donating substituents had important effects on the activities; for example, compounds **3f** (3-methoxy, 20%) and **3g** (4-methoxy, 45%) exhibited lower activities than **3h** (2, 3-dimethoxy, 70%) at 10 mg/kg. The positions and numbers of electron-withdrawing substituents had little effect on the activities; for example, **3l** (3-chlorine, 35%) and **3m** (4-chlorine, 40%) exhibited activities which were equivalent to **3n** (3, 4-dichlorine, 30%) at 10 mg/kg. Meanwhile, it was found that the electron-donating compound **3t** (4-dimethylamino, 35%) and electron-withdrawing compound **3w** (2-thiophene, 30%) exhibited activities which were equivalent to pymetrozine (30%) at 5 mg/kg.

When R was changed from phenyl to naphthyl, the insecticidal activity of the compound remained unchanged; such as in compound **3d** (phenyl, 30%), which exhibited activity equivalent to **3u** (naphthyl, 30%) at 10 mg/kg. In addition, while the benzene ring was replaced with other aromatic heterocycles (**3v**–**3w**), the insecticidal activities were enhanced. For example, compound **3w** (2-thiophene, 30%) exhibited the same level of activity as pymetrozine (30%) at 5 mg/kg.

The aphicidal activities of ketone hydrazone derivatives (**5a**–**5u**) are listed in Table 2. From Table 1 and Table 2, it can be seen that most aldehyde hydrazone derivatives exhibited better activities than the ketone hydrazone compounds.

#### 2.2.2. Larvicidal Activities Against Mosquito (*C. pipiens pallens*), Cotton Bollworm (*H. armigera*), Oriental Armyworm (*M. separata*) and Corn Borer (*O. nubilalis*)

The larvicidal activities of synthesized compounds **3a**–**3w**, **5a**–**5u** and pymetrozine against mosquitoes are shown in Table 3 and Table 4. Most compounds exhibited good larvicidal activities against *C. pipiens pallens*. In particular, the larvicidal activities of derivatives **3p**, **3u**, **3y**, **5g**, **5i**, **5l**, **5q** and **5u** were higher than that of pymetrozine, and compound **3u** (containing a 6-methoxy on the naphthyl) showed the highest larvicidal activity (40% at 0.1 mg/kg). The positions and numbers of substituents on the phenyl of these derivatives were critical for the activities against mosquitoes. For example, **5h** (3-methoxy) only showed 20% correcting mortality at 0.5 mg/kg, whereas **5i** (4-methoxy) exhibited 40% correcting mortality at 0.25 mg/kg. Meanwhile, **5j** (3,4-diemethoxy) and **5k** (3,4,5-triemethoxy) exhibited 50% and 30% correcting mortality at 0.5 mg/kg, respectively.

Compounds **3a**–**3w** and **5a**–**5u** have high stability. According to previous research on compounds with similar structures, compounds **3a**–**3w** and **5a**–**5u** may exhibit lower toxicity to non-target organisms. Compounds with higher nitrogen atom ratios may have relatively higher acute toxicity. Therefore, we predict that compounds **3t**, **3p**, **3q**, **3v** and **5u** may exhibit relatively higher toxicity. Therefore, compounds **3t** and **3w** can be further developed as potential insecticides.

#### 2.2.3. Fungicidal Activities

Compounds **3a**–**3w** and **5a**–**5u** were also evaluated for their fungicidal activities, with commercial fungicides carbendazim and chlorothalonil as controls (Table 5). The fungicidal activities test was repeated three times. Most derivatives showed broad-spectrum fungicidal activities against 14 kinds of phytopathogens. Overall, these derivatives showed excellent fungicidal activities against *P. piricola*. In particular, compound **5l** exhibited more than 50% inhibition against six kinds of fungi; compounds **5c**, **5f** and **5g** exhibited more than 50% inhibition against five kinds of fungi and compound **5t** exhibited a 90% inhibition rate against *P. piricola* at 50 mg/kg. The fungicidal activities of these compounds also exhibited a certain selectivity. At 50 mg/kg, 20 compounds exhibited a more than 50% inhibition rate against *Fusarium graminearum*, which was higher than chlorothalonil. A total of 7 compounds (against *Fusarium oxysporum* f. sp. *cucumeris*), 19 compounds (against *Cercospora arachidicola* Hori), 39 kinds of compounds (against *P. piricola*) and 13 compounds (against *Fusarium moniliforme*) showed a more than 50% inhibition rate at the concentration of 50 mg/kg, which was higher than the fungicidal activities of carbendazim.

## 3. Materials and Methods

Pymetrozine (Chemieliva Pharmaceutical Co., Ltd., Chongqing, China), carbendazim (Bailing Agrochemical Co., Ltd., Jiangyin, China), chlorothalonil (Bailing Agrochemical Co., Ltd., Beijing, China) and other reagents were used as received. All solvents were dried and purified using standard techniques. ^1^H NMR and ^13^C NMR spectra were obtained at 400 MHz using a Bruker (Billerica, MA, USA) AV400 spectrometer in CDCl_3_ or DMSO-*d_6_* solution with tetramethylsilane as the internal standard. Chemical shift values (*δ*) are given in parts per million. HRMS data were obtained on an FTICR-MS instrument (Ionspec 7.0 T, Agilent, Santa Clara, CA, USA). The melting points were determined using an X-4 binocular microscope melting-point apparatus and are uncorrected.

The biological assays for the foliar contact activity against bean aphid (*A. craccivora*), the larvicidal activities against mosquito larvae (*C. pipiens pallens*) and the stomach toxicity against cotton bollworm (*H. armigera*), oriental armyworm (*M. separata*) and corn borer (*O. nubilalis*) of compounds **3a**–**3w**, **5a**–**5u** and pymetrozine were conducted using the reported procedure [33,34,35]. The tests of fungicidal activities were carried out using the reported methods [36]. The testing process can also be found in the Supporting Information. The biological assays were repeated three times. The correcting mortality values were rounded to their nearest integers.

## 4. Conclusions

In summary, a series of novel triazone compounds containing aldehyde hydrazone or ketone hydrazone moieties were synthesized and evaluated for their insecticidal and fungicidal activities. Compounds **3t** and **3w** exhibited aphicidal activities comparable to pymetrozine. Compound **3u** showed the highest larvicidal activity against *C. pipiens pallens* (40% at 0.1 mg/kg). In addition, most derivatives exhibited broad-spectrum fungicidal activities against 14 kinds of plant fungi at 50 mg/kg. A total of 20 compounds exhibited a more than 50% inhibition rate against *F. graminearum* at 50 mg/kg and 39 compounds exhibited a more than 50% inhibition rate against *P. piricola*. Triazone compounds containing hydrazone moieties could be further explored as candidate insecticides against aphids.

## Figures and Tables

**Table 1:** Foliar contact activities against *A. craccivora* of compounds **3a**–**3w** and pymetrozine.

Compound	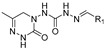 R_1_	Correcting Mortality (%) at Concentration (mg/kg)			
		600	100	10	5
**3a**	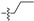	70 ± 2	30 ± 0	-^a^	-^a^
**3b**	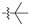	100 ± 0	85 ± 2	30 ± 0	-^a^
**3c**	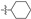	100 ± 0	70 ± 1	-^a^	-^a^
**3d**	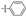	100 ± 0	95 ± 2	30 ± 0	-^a^
**3e**	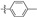	100 ± 0	90 ± 0	30 ± 0	-^a^
**3f**	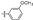	100 ± 0	70 ± 0	20 ± 0	-^a^
**3g**	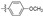	100 ± 0	95 ± 2	45 ± 2	-^a^
**3h**	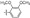	100 ± 0	100 ± 0	70 ± 0	15 ± 1
**3i**	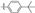	95 ± 1	90 ± 0	50 ± 0	-^a^
**3j**	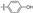	100 ± 0	100 ± 0	40 ± 0	-^a^
**3k**	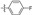	100 ± 0	95 ±2	30 ± 0	-^a^
**3l**	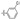	100 ± 0	95 ± 2	35	-^a^
**3m**	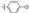	100 ± 0	100 ± 0	40 ± 0	-^a^
**3n**	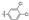	100 ± 0	90 ± 0	30 ± 0	-^a^
**3o**	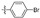	100 ± 0	95 ± 0	35 ± 0	-^a^
**3p**	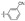	85 ± 2	40 ± 0	-^a^	-^a^
**3q**	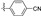	100 ± 0	90 ± 0	35 ± 1	-^a^
**3r**	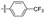	100 ± 0	80 ± 0	20 ± 0	-^a^
**3s**	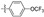	100 ± 0	90 ± 0	25 ± 2	-^a^
**3t**	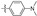	100 ± 0	100 ± 0	95 ± 0	35 ± 2
**3u**	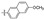	100 ± 0	90 ± 0	30 ± 0	-^a^
**3v**	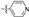	100 ± 0	95 ± 0	45 ± 0	-^a^
**3w**	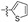	100 ± 0	100 ± 0	85 ± 2	30 ± 0
pymetrozine	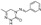	100 ± 0	100 ± 0	90 ± 0	30 ± 0

^a^-not tested.

**Table 2:** Foliar contact activities against *A. craccivora* of compounds **5a**–**5u** and pymetrozine.

Compound	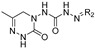 R_2_	Correcting Mortality (%) at Concentration (mg/kg)			
		600	100	10	5
**5a**	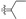	90 ± 0	30 ± 0	-^a^	-^a^
**5b**	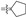	100 ± 0	35 ± 2	-^a^	-^a^
**5c**	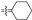	100 ± 0	40 ± 0	-^a^	-^a^
**5d**	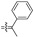	95 ± 2	70 ± 0	15 ± 1	-^a^
**5e**	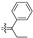	80 ± 0	45 ± 2	-^a^	-^a^
**5f**	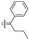	80 ± 0	30 ± 0	-^a^	-^a^
**5g**	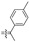	95 ± 2	60 ± 0	10 ± 0	-^a^
**5h**	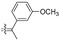	100 ± 0	50 ± 0	-^a^	-^a^
**5i**	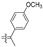	100 ± 0	65 ± 1	10 ± 0	-^a^
**5j**	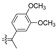	100 ± 0	80 ± 0	35 ± 2	-^a^
**5k**	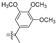	100 ± 0	95 ± 2	65 ± 2	-^a^
**5l**	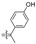	95 ± 2	80 ± 0	10 ± 0	-^a^
**5m**	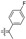	100 ± 0	70 ± 0	10 ± 0	-^a^
**5n**	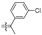	90 ± 0	30 ± 0	-^a^	-^a^
**5o**	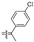	100 ± 0	75 ± 2	15 ± 1	-^a^
**5p**	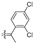	95 ± 1	85 ± 0	25 ± 1	-^a^
**5q**	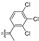	100 ± 0	95 ± 0	45 ± 0	-^a^
**5r**	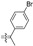	100 ± 0	80 ± 0	25 ± 0	-^a^
**5s**	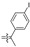	100 ± 0	95 ± 0	35 ± 0	-^a^
**5t**	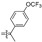	100 ± 0	85 ± 0	35 ± 1	-^a^
**5u**	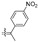	100 ± 0	90 ± 0	35 ± 1	-^a^
pymetrozine	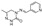	100 ± 0	100 ± 0	90 ± 0	30 ± 0

^a^—not tested.

**Table 3:** Insecticidal activities of compounds **3a**–**3w**, **5a**–**5u** and pymetrozine against *C. pipiens pallens*.

Compound	Correcting Mortality (%) at Concentration (mg/kg)						
	10	5	2	1	0.5	0.25	0.1
**3a**	30 ± 0	-^a^	-^a^	-^a^	-^a^	-^a^	-^a^
**3b**	100 ± 0	0	-^a^	-^a^	-^a^	-^a^	-^a^
**3c**	100 ± 0	100 ± 0	100 ± 0	40 ± 0	-^a^	-^a^	-^a^
**3d**	40 ± 0	-^a^	-^a^	-^a^	-^a^	-^a^	-^a^
**3e**	100 ± 0	100 ± 0	50 ± 0	-^a^	-^a^	-^a^	-^a^
**3f**	100 ± 0	100 ± 0	60 ± 0	-^a^	-^a^	-^a^	-^a^
**3g**	100 ± 0	100 ± 0	40 ± 0	-^a^	-^a^	-^a^	-^a^
**3h**	100 ± 0	100 ± 0	100 ± 0	20 ± 0	-^a^	-^a^	-^a^
**3i**	100 ± 0	40 ± 0	-^a^	-^a^	-^a^	-^a^	-^a^
**3j**	100 ± 0	100 ± 0	100 ± 0	100 ± 0	60 ± 0	-^a^	-^a^
**3k**	100 ± 0	20 ± 0	-	-	-	-^a^	-^a^
**3l**	100 ± 0	100 ± 0	100 ± 0	100 ± 0	60 ± 0	-^a^	-^a^
**3m**	100 ± 0	100 ± 0	100 ± 0	100 ± 0	40 ± 0	-^a^	-^a^
**3n**	100 ± 0	100 ± 0	70 ± 0	-^a^	-^a^	-^a^	-^a^
**3o**	80 ± 0	-^a^	-^a^	-^a^	-^a^	-^a^	-^a^
**3p**	100 ± 0	100 ± 0	100 ± 0	100 ± 0	100 ± 0	40 ± 0	-^a^
**3q**	100 ± 0	100 ± 0	20 ± 0	-^a^	-^a^	-^a^	-^a^
**3r**	100 ± 0	100 ± 0	100 ± 0	100 ± 0	60 ± 0	-^a^	-^a^
**3s**	100 ± 0	100 ± 0	10 ± 0	-^a^	-^a^	-^a^	-^a^
**3t**	30 ± 0	-^a^	-^a^	-^a^	-^a^	-^a^	-^a^
**3u**	100 ± 0	100 ± 0	100 ± 0	100 ± 0	100 ± 0	100 ± 0	40 ± 0
**3v**	100 ± 0	100 ± 0	100 ± 0	100 ± 0	100 ± 0	20 ± 0	-^a^
**3w**	100 ± 0	100 ± 0	100 ± 0	100 ± 0	20 ± 0	-^a^	-^a^
**5a**	100 ± 0	100 ± 0	40 ± 0	-^a^	-^a^	-^a^	-^a^
**5b**	100 ± 0	100 ± 0	100 ± 0	100 ± 0	20 ± 0	-^a^	-^a^
**5c**	50 ± 0	-^a^	-^a^	-^a^	-^a^	-^a^	-^a^
**5d**	30 ± 0	-^a^	-^a^	-^a^	-^a^	-^a^	-^a^
**5e**	30 ± 0	-^a^	-^a^	-^a^	-^a^	-^a^	-^a^
**5f**	60 ± 0	-^a^	-^a^	-^a^	-^a^	-^a^	-^a^
**5g**	100 ± 0	100 ± 0	100 ± 0	100 ± 0	100 ± 0	20 ± 0	-^a^
**5h**	100 ± 0	100 ± 0	100 ± 0	80 ± 0	20 ± 0	-^a^	-^a^
**5i**	100 ± 0	100 ± 0	100 ± 0	100 ± 0	100 ± 0	40 ± 0	-^a^
**5j**	100 ± 0	100 ± 0	100 ± 0	100 ± 0	50 ± 0	-^a^	-^a^
**5k**	100 ± 0	100 ± 0	100 ± 0	100 ± 0	30 ± 0	-^a^	-^a^
**5l**	100 ± 0	100 ± 0	100 ± 0	100 ± 0	100 ± 0	40 ± 0	-^a^
**5m**	100 ± 0	100 ± 0	100 ± 0	100 ± 0	20 ± 0	-^a^	-^a^
**5n**	100 ± 0	100 ± 0	100 ± 0	100 ± 0	60 ± 0	-^a^	-^a^
**5o**	100 ± 0	100 ± 0	100 ± 0	100 ± 0	100 ± 0	40 ± 0	-^a^
**5p**	100 ± 0	100 ± 0	100 ± 0	60 ± 0	-^a^	-^a^	-^a^
**5q**	100 ± 0	100 ± 0	100 ± 0	80 ± 0	20 ± 0	-^a^	-^a^
**5r**	80 ± 0	40 ± 0	-^a^	-^a^	-^a^	-^a^	-^a^
**5s**	90 ± 0	30± 0	-^a^	-^a^	-^a^	-^a^	-^a^
**5t**	70 ± 0	-^a^	-^a^	-^a^	-^a^	-^a^	-^a^
**5u**	100 ± 0	100 ± 0	100 ± 0	100 ± 0	20 ± 0	-^a^	-^a^
pymetrozine	100 ± 0	40 ± 0	-^a^	-^a^	-^a^	-^a^	-^a^

^a^—not tested.

**Table 4:** Insecticidal activities of compounds **3a**–**3w**, **5a**–**5u** and pymetrozine against *H. armigera*, *O. nubilalis* and *M. separata* at 600 mg/kg.

Compound	Correcting Mortality (%) at Concentration 600 mg/kg mg/kg)		
	*H. armigera*	*O. ubilalis*	*M. eparata*
**3a**	15 ± 0	15 ± 0	10 ± 0
**3b**	0	0	5 ± 0
**3c**	25 ± 0	15 ± 0	20 ± 0
**3d**	15 ± 0	10 ± 0	20 ± 0
**3e**	5 ± 0	20 ± 0	20 ± 0
**3f**	15 ± 0	5 ± 0	15 ± 0
**3g**	0	0	0
**3h**	20 ± 0	15 ± 0	25 ± 0
**3i**	0	0	5 ± 0
**3j**	25 ± 0	15 ± 0	25 ± 0
**3k**	10 ± 0	5 ± 0	20 ± 0
**3l**	55 ± 0	45 ± 0	70 ± 0
**3m**	15 ± 0	10 ± 0	20 ± 0
**3n**	55 ± 0	45 ± 0	65 ± 0
**3o**	0	0	5 ± 0
**3p**	100 ± 0/100 ^a^ ± 0/ 20^b^ ± 0	100 ± 0/100 ^a^ ± 0/30 ^b^ ± 0	100 ± 0/100 ^a^ ± 0/20 ^b^ ± 0
**3q**	25 ± 0	20 ± 0	35 ± 0
**3r**	35 ± 0	25 ± 0	45 ± 0
**3s**	10 ± 0	20 ± 0	20 ± 0
**3t**	100 ± 0/100 ^a^ ± 0/30 ^b^ ± 0	100 ± 0/100 ^a^ ± 0/20 ^b^ ± 0	100 ± 0/100 ^a^ ± 0/60 ^b^ ± 0
**3u**	15 ± 0	10 ± 0	15 ± 0
**3v**	15 ± 0	10 ± 0	25 ± 0
**3w**	35 ± 0	25 ± 0	40 ± 0
**5a**	15 ± 0	10 ± 0	25 ± 0
**5b**	55 ± 0	50 ± 0	80 ± 0
**5c**	20 ± 0	10 ± 0	20 ± 0
**5d**	0	0	0
**5e**	15 ± 0	10 ± 0	20 ± 0
**5f**	50 ± 0	45 ± 0	65 ± 0
**5g**	20 ± 0	15 ± 0	25 ± 0
**5h**	35 ± 0	20 ± 0	25 ± 0
**5i**	55 ± 0	50 ± 0	65 ± 0
**5j**	25 ± 0	30 ± 0	10 ± 0
**5k**	10 ± 0	25 ± 0	15 ± 0
**5l**	50 ± 0	40 ± 0	60 ± 0
**5m**	0	0	5 ± 0
**5n**	20 ± 0	15 ± 0	30 ± 0
**5o**	55 ± 0	45 ± 0	65 ± 0
**5p**	20 ± 0	25 ± 0	25 ± 0
**5q**	30 ± 0	15 ± 0	15 ± 0
**5r**	0	0	5 ± 0
**5s**	30 ± 0	25 ± 0	15 ± 0
**5t**	20 ± 0	15 ± 0	20 ± 0
**5u**	20 ± 0	10 ± 0	20 ± 0
pymetrozine	20 ± 0	40 ± 0	50 ± 0

^a^ Mortality at 200 mg/L, ^b^ Mortality at 100 mg/L.

**Table 5:** In vitro fungicidal activities of compounds **3a**–**3w**, **5a**–**5u**, carbendazim and chlorothalonil against 14 kinds of fungi.

Compound	Fungicidal Activities (%) ^a^ at 50 mg/kg													
	AS ^b^	FG ^b^	PI ^b^	PC ^b^	SS ^b^	BC ^b^	RS ^b^	FC ^b^	CH ^b^	PP ^b^	RC ^b^	BM ^b^	WA ^b^	FM ^b^
**3a**	33 ± 1	13 ± 1	4 ± 1	14 ± 1	11 ± 1	12 ± 1	28 ± 1	8 ± 3	0	21 ± 1	18 ± 1	42 ± 2	27 ± 2	11 ± 1
**3b**	6 ± 1	11 ± 2	11 ± 1	23 ± 1	13 ± 1	7 ± 3	17 ± 2	47 ± 2	42 ± 3	86 ± 3	79 ± 1	35 ± 1	47 ± 1	58 ± 1
**3c**	9 ± 2	38 ± 1	14 ± 2	27 ± 2	37 ± 1	18 ± 1	22 ± 2	55 ± 1	60 ± 1	85 ± 1	85 ± 1	55 ± 2	39 ± 1	53 ± 1
**3d**	31 ± 3	26 ± 1	19 ± 1	37 ± 3	25 ± 1	5 ± 1	36 ± 1	12 ± 1	0	37 ± 2	38 ± 2	25 ± 1	27 ± 3	19 ± 1
**3e**	21 ± 1	26 ± 1	12 ± 1	20 ± 2	11 ± 1	11 ± 1	8 ± 2	6 ± 1	25 ± 1	72 ± 1	58 ± 1	25 ± 1	20 ± 2	21 ± 1
**3f**	12 ± 1	76 ± 1	9 ± 2	10 ± 1	25 ± 3	5 ± 1	43 ± 2	35 ± 1	50± 1	65 ± 2	68 ± 1	35 ± 1	52 ± 2	38 ± 1
**3g**	31 ± 2	30 ± 1	9 ± 1	10 ± 1	20 ± 2	10 ± 2	33 ± 1	45 ± 1	56 ± 1	73 ± 3	79 ± 1	65 ± 1	39 ± 1	20 ± 1
**3h**	6 ± 1	38 ± 1	14 ± 1	34 ± 2	37 ± 3	36 ± 2	57 ± 2	42 ± 2	44 ± 1	60 ± 2	71 ± 1	30 ± 2	32 ± 1	36 ± 1
**3i**	12 ± 1	38 ± 2	4 ± 2	24 ± 1	25 ± 3	13 ± 1	45 ± 2	32 ± 1	42 ± 3	65 ± 1	42 ± 1	40 ± 1	44 ± 2	48 ± 1
**3j**	12 ±2	69 ± 1	14 ± 1	10 ± 1	13 ± 2	13 ± 1	26 ± 1	35 ± 1	39 ± 1	57 ± 1	56 ± 2	47 ± 1	41 ± 2	38 ± 1
**3k**	6 ± 1	57 ± 2	14 ± 1	41 ± 1	15 ± 1	2 ± 2	33 ± 1	32 ± 2	57 ± 1	70 ± 1	49 ± 1	47 ± 1	33 ± 1	32 ± 1
**3l**	12 ± 2	30 ± 2	4 ± 1	6 ± 3	29 ± 1	23 ± 2	40 ± 1	45 ± 1	48 ± 1	77 ± 1	72 ± 2	42 ± 1	25 ± 1	50 ± 1
**3m**	12 ± 2	73 ± 1	14 ± 1	13 ± 2	15 ± 1	5 ± 1	14 ± 1	32 ± 2	46 ± 1	54 ± 1	60 ± 1	42 ± 1	44 ± 1	38 ± 2
**3n**	18 ± 1	11 ± 1	9 ± 2	37 ± 2	20 ± 2	18 ± 1	45 ± 2	32 ± 2	44 ± 1	52 ± 1	49 ± 1	47 ± 2	25 ± 1	46 ± 2
**3o**	25 ± 1	61 ± 1	19 ± 1	17 ± 1	20 ± 2	7 ± 3	24 ± 2	45 ± 1	50 ± 1	72 ± 1	62 ± 2	40 ± 1	30 ± 2	45 ± 1
**3p**	18 ± 1	50 ± 1	14 ± 1	44 ± 1	15 ± 1	18 ± 1	19 ± 1	27 ± 1	50 ± 1	60 ± 2	56 ± 1	47 ± 2	41 ± 3	41 ± 3
**3q**	12 ± 2	50 ± 1	14 ± 1	37 ± 1	34 ± 1	10 ± 2	10 ± 2	30 ± 1	56 ± 1	73 ± 3	74 ± 1	42 ± 2	39 ± 1	46 ± 2
**3r**	6 ± 1	34 ± 1	23 ± 2	34 ± 1	17 ± 1	13 ± 1	22 ± 2	22 ± 1	32 ± 1	65 ± 2	50 ± 1	12 ± 2	0	16 ± 1
**3s**	6 ± 2	25 ± 2	16 ± 2	35 ± 1	17 ± 3	25 ± 2	26 ± 2	32 ± 2	35 ± 2	56 ± 1	58 ± 1	42 ± 1	31 ± 1	15 ± 1
**3t**	12 ± 1	51 ± 1	4 ± 1	10 ± 1	27 ± 2	31 ± 2	54 ± 1	35 ± 1	50 ± 1	78 ± 2	62 ± 1	47 ± 2	52 ± 2	41 ± 3
**3u**	12 ± 1	34 ± 2	4 ± 1	17 ± 1	24 ± 1	5 ± 1	40 ± 1	35 ± 1	44 ± 1	72 ± 1	72 ± 3	35 ± 1	39 ± 1	33 ± 1
**3v**	17 ± 2	11 ± 1	11.8	11 ± 1	11 ± 2	7 ± 2	15 ± 1	47 ± 2	57 ± 1	83 ± 2	78 ± 1	60 ± 1	55 ± 2	58 ± 1
**3w**	5 ± 1	17 ± 2	8 ± 3	11 ± 1	16 ± 1	7 ± 2	13 ± 1	47 ± 2	50 ± 1	78 ± 2	78 ± 1	40 ± 1	27 ± 2	41 ± 3
**5a**	18 ± 3	61 ± 1	23 ± 1	41 ± 1	10 ± 1	5 ± 1	22 ± 3	47 ± 1	48 ± 2	78 ± 2	71 ± 2	32 ± 2	35 ± 2	40 ± 1
**5b**	18 ± 1	11 ± 2	4 ± 2	17 ± 1	15 ± 2	10 ± 2	49 ± 1	60 ± 1	60 ±2	80 ± 1	82 ± 2	57 ± 2	42 ± 2	46 ± 2
**5c**	18 ± 1	73 ± 1	14 ± 1	10 ± 1	25 ± 3	13 ± 1	24 ± 2	60 ± 1	56 ± 2	82 ± 1	84 ± 1	50 ± 1	42 ± 2	53 ± 1
**5d**	25 ± 2	76 ± 2	14 ± 1	31 ± 1	24 ± 1	10 ± 1	42 ± 1	45 ± 2	44 ± 1	85 ± 1	86 ± 1	57 ± 1	50 ± 1	56 ± 1
**5e**	12 ± 1	73 ± 3	4 ± 2	27 ± 2	34 ± 1	26 ± 1	45 ± 1	45 ± 1	44 ± 1	72 ± 1	82 ± 2	52 ± 1	35 ± 1	56 ± 1
**5f**	25 ± 1	73 ± 2	33 ± 1	31 ± 1	29 ± 1	31 ± 2	22 ± 2	51 ± 1	56 ± 1	65 ± 2	67 ± 3	55 ± 1	39 ± 1	56 ± 1
**5g**	18 ± 2	57 ± 2	14 ± 1	10 ± 1	15 ± 1	10 ± 1	49 ± 1	52 ± 2	52 ± 1	70 ± 2	71 ± 1	60 ± 1	42 ± 2	63 ± 1
**5h**	18 ± 1	26 ± 3	9 ± 1	17 ± 1	16 ± 1	16 ± 1	16 ± 1	42 ± 1	49 ± 1	54 ± 1	59 ± 1	42 ± 1	34 ± 1	37 ± 1
**5i**	18 ± 1	53 ± 1	9 ± 2	31 ± 1	37 ± 3	13 ± 1	10 ± 1	47 ± 2	52 ± 1	86 ± 2	80 ± 1	57 ± 2	57 ± 1	56 ± 2
**5j**	12 ± 1	44 ± 2	11 ± 1	11 ± 2	23 ± 2	24 ± 1	31 ± 1	47 ± 2	45 ± 2	49 ± 1	54 ± 2	39 ± 1	38 ± 2	27 ± 2
**5k**	17 ± 2	52 ± 1	15 ± 3	10 ± 2	17 ± 1	6 ± 1	24 ± 3	50 ± 1	53 ± 1	42 ± 1	52 ± 1	47 ± 1	39 ± 1	23 ± 1
**5l**	50 ± 1	61 ± 2	23 ± 3	17 ± 1	24 ± 1	5 ± 1	28 ± 1	57 ± 2	52 ± 1	75 ± 1	59 ± 1	40 ± 1	35 ± 2	53 ± 1
**5m**	31 ± 1	61 ± 2	33 ± 1	37 ± 1	15 ± 1	10 ± 2	15 ± 3	45 ± 1	36 ± 1	82 ± 1	64 ± 1	47 ± 2	39 ± 1	70 ± 1
**5n**	10 ± 1	23 ± 1	14 ± 2	14 ± 1	30 ± 1	30 ± 2	21 ± 2	47 ± 2	41 ± 2	51 ± 1	52 ± 1	38 ± 1	25 ± 1	24 ± 1
**5o**	18 ± 2	23 ± 1	4 ± 3	75 ± 1	17 ± 1	13 ± 1	10 ± 2	42 ± 2	36 ± 1	68 ± 2	59 ± 1	42 ± 2	14 ± 1	26 ± 1
**5p**	9 ± 2	18 ± 3	7 ± 3	24 ± 2	18 ± 2	15 ± 1	31 ± 1	26 ± 2	29 ± 3	46 ± 2	45 ± 2	40 ± 1	16 ± 1	18 ± 2
**5q**	15 ± 2	24 ± 2	9 ± 1	16 ± 3	17 ± 2	11 ± 2	17 ± 3	36 ± 3	34 ± 2	52 ± 2	57 ± 2	37 ± 3	29 ± 1	13 ± 1
**5r**	25 ± 1	53 ± 1	23 ± 3	10 ± 1	15 ± 2	18 ± 1	10 ± 2	27 ± 1	44 ± 1	68 ± 3	70 ± 2	42 ± 1	25 ± 1	46 ± 2
**5s**	17 ± 1	42 ± 2	13 ± 1	8 ± 2	14 ± 2	9 ± 3	14 ± 2	31 ± 1	42 ± 2	50 ± 3	56 ± 2	42 ± 2	24 ± 3	45 ± 1
**5t**	12 ± 1	73 ± 1	23 ± 2	6 ± 1	12 ± 1	18 ± 1	28 ± 1	47 ± 2	56 ± 1	90 ± 1	91 ± 1	35 ± 1	39 ± 1	66 ± 2
**5u**	6 ± 1	30 ± 1	14 ± 2	24 ± 1	24 ± 1	5 ± 1	10 ± 1	37 ± 2	52 ± 1	65 ± 2	67 ± 3	60 ± 1	50 ± 1	30 ± 1
Carbendazim ^c^	<50	100	100	<50	100	<50	100	<50	<50	<50	100	100	100	<50
Chlorothalonil ^c^	73 ± 1	<50	86 ± 1	100	<50	100	100	100	73 ± 1	100	100	91 ± 1	91 ± 1	100

^a^ Average of three replicates. All results are expressed as the mean ± SD. ^b^ Abbreviations: AS—*Alternaria solani*; FG—*Fusarium graminearum*; PI—*Phytophthora infestans*; PC—*Phytophthora capsici*; SS—*Sclerotinia sclerotiorum*; BC—*Botrytis cinerea*; RS—*Rhizoctonia solani.*; FC—*Fusarium oxysporum* f. sp. *cucumeris*; CH—*Cercospora arachidicola Hori*; PP—*Physalospora piricola*; RC—*Rhizoctonia cerealis*; BM—*Bipolaris maydis*; WA—*watermelon anthracnose*; FM—*Fusarium moniliforme*. ^c^ commercial agricultural fungicides were used for comparison of antifungal activities.

## Data Availability

Data will be made available on request.

## Supplementary Materials

The following supporting information can be downloaded at: https://www.mdpi.com/article/10.3390/molecules30020340/s1, File S1: the general preparation process of **3a–3w** and **5a–5w**, ^1^H and ^13^C NMR data and spectra of **3a–3w** and **5a–5w**, detailed bioassay procedures for the anti-TMV activities and fungicidal activities.
