# Supplementary material for: Synthesis and Insecticidal/Fungicidal Activities of Triazone Derivatives Containing Acylhydrazone Moieties
Source: Molecules. 2025 Jan 16;30(2):340. doi: 10.3390/molecules30020340 (PMC11767570; doi:10.3390/molecules30020340)
Supplement: Supplementary file 1 [file molecules-30-00340-s001.zip › molecules-3368665-supplementary.pdf]

Supporting Information

Synthesis and Insecticidal/Fungicidal Activities of Triazone Derivatives Containing Acylhydrazone Moiety

Peipei Cui[1], Yan Yang[2]*

[1]College of Architecture and Arts, Taiyuan University of Technology, Jinzhong Shanxi 030060, People's Republic of China

[2]College of Chemistry and Chemical Engineering, Taiyuan University of Technology, Taiyuan Shanxi 030024, People's Republic of China

*Corresponding author: cui_peipei@163.com (Peipei Cui); yy529222@163.com (Yan Yang)

The general preparation process of **3a-3w**…………………….……………….……………..…….… S1–S9

The general preparation process of **5a-5u**……………….………….………….…………. S9–S15

^{1}H NMR and ^{13}C NMR data for compounds **3a-3w** and **5a-5u**…………………….…….…S16–S59

Biological assay……………………………………………………………....…...…..…S60–S61

The general preparation process of 3a-3w. To a solution of compound **1** (0.74g, 4 mmol) and *p*-toluene sulfonic acid (0.14 g, 0.80 mmol)in methanol (300 mL) was added the corresponding aldehydes **2** (4 mmol), and then the mixture was refluxed for 6 h. The solution was cooled to room temperature, and evaporated under reduced pressure to remove methanol until a large amount of solid precipitates. The resulting precipitate was collected by filtration in vacuo and was washed with methanol to provide compounds **3a-3w**.

(*E*)-N-(6-methyl-3-oxo-2,3-dihydro-1,2,4-triazin-4(*5H*)-yl)-2-propylidenehydrazine carboxamide(**3a**). White solid, m.p. 173-175 °C, yield 57%; ^{1}H NMR (400 MHz, $CDCl_3$) δ 8.63 (s, 1H), 7.90 (s, 1H), 7.66 (s, 1H), 7.08 (t, J = 5.2 Hz,1H), 4.25 (s, 2H), 2.21 (m, 2H), 1.95 (s, 3H), 1.54 (m, 2H), 0.95 (t, J = 7.6 Hz, 3H); ^{13}C NMR (100 MHz, $CDCl_3$) δ 155.4, 152.0, 147.3, 145.6, 53.0, 34.3, 20.4, 19.8, 13.9; HRMS(ESI) m/z $[M+H]^+$ calcd. for $C_9H_{16}N_6O_2$: 241.1408, found: 241.1407.

(*E*)-2-(2,2-dimethylpropylidene)-N-(6-methyl-3-oxo-2,3-dihydro-1,2,4-triazin-4(*5H*)-yl)hydrazinecarboxamide (**3b**). White solid, m.p. 230-231 °C, yield 67%; ^{1}H NMR (400 MHz, DMSO-d_6) δ 10.25 (s, 1H), 9.73 (s, 1H), 8.60 (s, 1H), 7.12 (s, 1H), 4.06 (s, 2H), 1.84 (s, 3H), 1.05 (s, 9H); ^{13}C NMR (100 MHz, DMSO-d_6) δ 154.6, 152.5, 151.7, 144.8, 52.2, 34.2, 27.4, 19.8; HRMS(ESI) m/z $[M+H]^+$ calcd. for $C_{10}H_{19}N_6O_2$: 255.1564, found: 255.1567.

(*E*)-2-(cyclohexylmethylene)-N-(6-methyl-3-oxo-2,3-dihydro-1,2,4-triazin-4(*5H*)-yl)hydrazinecarboxamide (**3c**). White solid, m.p. 213-214 °C, yield 75%; ^{1}H NMR (400 MHz, DMSO-d_6) δ 10.26 (s, 1H), 9.72 (s, 1H), 8.64 (s, 1H), 7.10 (d, J = 4.0 Hz, 1H),

4.05 (s, 2H), 1.84 (s, 3H), 1.76–1.68 (m, 4H), 1.64–1.61 (m, 1H), 1.27–1.14 (m, 6H); ^{13}C NMR (100 MHz, DMSO-*d₆*) *δ* 154.6, 151.6, 149.0, 144.8, 52.2, 29.8, 25.6, 25.1, 19.9; HRMS(ESI) m/z [M+H]$^+$ calcd. for $C_{12}H_{21}N_6O_2$: 281.1721, found: 281.1725.

(*E*)-2-benzylidene-N-(6-methyl-3-oxo-2,3-dihydro-1,2,4-triazin-4(*5H*)-yl)hydrazine carboxamide (**3d**). White solid, m.p. 219-220 °C, yield 69%; ^{1}H NMR (400 MHz, DMSO-d_6) *δ* 10.77 (s, 1H), 9.78 (s, 1H), 9.25 (s, 1H), 7.87 (s, 1H), 7.78 (d, *J* = 6.8 Hz, 2H), 7.43–7.37 (m, 3H), 4.12 (s, 2H), 1.87 (s, 3H); ^{13}C NMR (100 MHz, DMSO-d_6) *δ* 154.7, 151.7, 144.9, 141.1, 134.4, 129.5, 128.6, 126.9, 52.2, 19.9; HRMS(ESI) m/z [M+H]$^+$ calcd. for $C_{12}H_{15}N_6O_2$: 275.1251, found: 275.1246.

(*E*)-N-(6-methyl-3-oxo-2,3-dihydro-1,2,4-triazin-4(*5H*)-yl)-2-(4-methylbenzylidene)hydrazinecarboxamide (**3e**). White solid, m.p. 264-265 °C, yield 78%; ^{1}H NMR (400 MHz, DMSO-d_6): *δ* 10.69 (s, 1H), 9.76 (s, 1H), 9.20 (s, 1H), 7.83 (s, 1H), 7.67 (d, *J* = 7.2 Hz, 2H), 7.22 (d, *J* = 7.2 Hz, 2H), 4.11 (s, 2H), 2.32 (s, 3H), 1.87 (s, 3H); ^{13}C NMR (100 MHz, DMSO-d_6) *δ* 154.7, 151.8, 144.9, 141.2, 139.2, 131.8, 129.3, 126.9, 52.2, 21.0, 19.9; HRMS(ESI) m/z [M+H]$^+$ calcd. for $C_{13}H_{17}N_6O_2$: 289.1408, found: 289.1413.

(*E*)-2-(3-methoxybenzylidene)-N-(6-methyl-3-oxo-2,3-dihydro-1,2,4-triazin-4(*5H*)-yl)hydrazinecarboxamide (**3f**). White solid, m.p. 226-227 °C, yield 67%; ^{1}H NMR (400 MHz, DMSO-d_6) *δ* 10.78 (s, 1H), 9.78 (s, 1H), 9.29 (s, 1H), 7.83 (s, 1H), 7.43 (s, 1H), 7.31 (t, *J* = 7.2 Hz, 1H), 7.25 (d, *J* = 7.2 Hz, 1H), 6.94 (d, *J* = 7.2 Hz, 1H), 4.12 (s, 2H), 3.80 (s, 3H), 1.87 (s, 3H); ^{13}C NMR (100 MHz, DMSO-d_6) *δ* 159.6, 154.6, 151.8, 144.9, 141.0, 135.8, 129.7, 120.2, 115.8, 110.8, 55.3, 52.2, 19.9; HRMS(ESI) m/z [M+H]$^+$ calcd. for $C_{13}H_{17}N_6O_3$: 305.1357, found: 305.1359.

(*E*)-2-(4-methoxybenzylidene)-N-(6-methyl-3-oxo-2,3-dihydro-1,2,4-triazin-4(*5H*)-yl)hydrazinecarboxamide (**3g**). White solid, m.p. 230-231 °C, yield 82%; ^{1}H NMR (400 MHz, DMSO-*d*$_6$) *δ* 10.62 (s, 1H), 9.76 (s, 1H), 9.17 (s, 1H), 7.80 (s, 1H), 7.72 (d, *J* = 8.0 Hz, 2H), 6.96 (d, *J* = 8.0 Hz, 2H), 4.11 (s, 2H), 3.79 (s, 3H), 1.87 (s, 3H); ^{13}C NMR (100 MHz, DMSO-*d*$_6$) *δ* 160.4, 154.7, 151.7, 144.8, 141.0, 128.5, 127.1, 114.1, 55.3, 52.2, 19.9; HRMS(ESI) m/z: [M+H]$^+$ calcd. for $C_{13}H_{17}N_6O_3$: 305.1357, found: 305.1359.

(*E*)-2-(2,3-dimethoxybenzylidene)-N-(6-methyl-3-oxo-2,3-dihydro-1,2,4-triazin-4(*5H*)-yl)hydrazinecarboxamide (**3h**). White solid, m.p. 226-227 °C, yield 80%; ^{1}H NMR (400 MHz, DMSO-*d*$_6$) *δ* 10.75 (s, 1H), 9.77 (s, 1H), 9.21 (s, 1H), 8.16 (s, 1H), 7.68 (d, *J* = 6.8 Hz, 1H), 7.10–7.04 (m, 2H), 4.10 (s, 2H), 3.82 (s, 3H), 3.74 (s, 3H), 1.86 (s, 3H); ^{13}C NMR (100 MHz, DMSO-*d*$_6$) *δ* 154.6, 152.6, 151.7, 147.5, 144.8, 136.7, 127.8, 124.1, 117.4, 113.6, 61.2, 55.7, 52.2, 19.9; HRMS(ESI) m/z [M+H]$^+$ calcd. for $C_{14}H_{19}N_6O_4$: 335.1462, found: 335.1469.

(*E*)-2-(4-tert-butylbenzylidene)-N-(6-methyl-3-oxo-2,3-dihydro-1,2,4-triazin-4(*5H*)-yl)hydrazinecarboxamide (**3i**). White solid, m.p. 211-212 °C, yield 69%; ^{1}H NMR (400 MHz, DMSO-*d*$_6$) *δ* 10.71(s, 1H), 9.77 (s, 1H), 9.19 (s, 1H), 7.84 (s, 1H), 7.69 (d, *J* = 7.2 Hz, 2H), 7.42 (d, *J* = 7.2 Hz, 2H), 4.12 (s, 2H), 1.87 (s, 3H), 1.29 (s, 9H); ^{13}C NMR (100 MHz, DMSO-*d*$_6$) *δ* 154.7, 152.2, 151.7, 144.8, 141.1, 131.7, 126.7, 125.4, 52.2, 34.6, 31.0, 19.9; HRMS(ESI) m/z [M+H]$^+$ calcd. for $C_{16}H_{23}N_6O_2$: 331.1877, found: 331.1882.

(*E*)-2-(4-hydroxybenzylidene)-N-(6-methyl-3-oxo-2,3-dihydro-1,2,4-triazin-4(*5H*)-yl)hydrazinecarboxamide (**3j**). White solid, m.p. 237-238 °C, yield 74%; ^{1}H NMR (400 MHz, DMSO-*d*$_6$) *δ* 10.54 (s, 1H), 9.81 (s, 1H), 9.75 (s, 1H), 9.11 (s, 1H), 7.76 (s, 1H),

7.59 (d, J = 8.0 Hz, 2H), 6.77 (d, J = 8.0 Hz, 2H), 4.10 (s, 2H), 1.86 (s, 3H); ^{13}C NMR (100 MHz, DMSO-d_6) δ 158.9, 154.8, 151.8, 144.8, 141.4, 128.6, 125.5, 115.5, 52.2, 19.9; HRMS(ESI) m/z $[M+H]^+$ calcd. for $C_{12}H_{15}N_6O_3$: 291.1200, found: 291.1200.

(*E*)-2-(4-fluorobenzylidene)-N-(6-methyl-3-oxo-2,3-dihydro-1,2,4-triazin-4(*5H*)-yl) hydrazinecarboxamide (**3k**). White solid, m.p. 240-241 °C, yield 75%; ^{1}H NMR (400 MHz, DMSO-d_6) δ 10.77 (s, 1H), 9.77 (s, 1H), 9.27 (s, 1H), 7.87 (s, 1H), 7.85 (d, J = 8.0 Hz, 2H), 7.25 (t, J = 8.0 Hz, 2H), 4.11 (s, 2H), 1.87 (s, 3H); ^{13}C NMR (100 MHz, DMSO-d_6) δ 162.8 (d, J=245.4 Hz), 154.7, 151.7, 144.8, 139.9, 131.7 (d, J = 2.4 Hz), 129.0 (d, J = 8.2 Hz), 115.6 (d, J = 21.6 Hz), 52.2, 19.8; HRMS(ESI) m/z $[M+H]^+$ calcd. for $C_{12}H_{14}FN_6O_2$: 293.1157, found: 293.1159.

(*E*)-2-(3-chlorobenzylidene)-N-(6-methyl-3-oxo-2,3-dihydro-1,2,4-triazin-4(*5H*)-yl) hydrazinecarboxamide (**3l**). White solid, m.p. 242-243 °C, yield 88%; ^{1}H NMR (400 MHz, DMSO-d_6) δ 10.89 (s, 1H), 9.79 (s, 1H), 9.42 (s, 1H), 8.05 (s, 1H), 7.84 (s, 1H), 7.61 (d, J = 3.2 Hz, 1H), 7.44–7.41 (m, 2H), 4.12 (s, 2H), 1.87 (s, 3H); ^{13}C NMR (100 MHz, DMSO-d_6) δ 154.6, 151.8, 144.9, 139.5, 136.7, 133.8, 130.5, 129.1, 126.3, 125.6, 52.2, 19.9; HRMS(ESI) m/z $[M+H]^+$ calcd. for $C_{12}H_{14}ClN_6O_2$: 309.0861, found: 309.0867.

(*E*)-2-(4-chlorobenzylidene)-N-(6-methyl-3-oxo-2,3-dihydro-1,2,4-triazin-4(*5H*)-yl) hydrazinecarboxamide (**3m**). White solid, m.p. 275-276 °C, yield 85%; ^{1}H NMR (400 MHz, DMSO-d_6) δ 10.86 (s, 1H), 9.79 (s, 1H), 9.33 (s, 1H), 7.85 (s, 1H), 7.83 (d, J = 8.0 Hz, 2H), 7.47 (d, J = 8.0 Hz, 2H), 4.11 (s, 2H), 1.87 (s, 3H); ^{13}C NMR (100 MHz, DMSO-d_6) δ 154.6, 151.7, 144.9, 139.7, 133.8, 133.4, 128.7, 128.6, 52.2, 19.9;

HRMS(ESI) m/z $[M+H]^+$ calcd. for $C_{12}H_{14}ClN_6O_2$: 309.0861, found: 309.0861.

(*E*)-2-(3,4-dichlorobenzylidene)-N-(6-methyl-3-oxo-2,3-dihydro-1,2,4-triazin-4(*5H*)-yl)hydrazinecarboxamide (**3n**). White solid, m.p. 265-266 °C, yield 88%; ^{1}H NMR (400 MHz, DMSO-*d6*) *δ* 10.97 (s, 1H), 9.80 (s, 1H), 9.47 (s, 1H), 8.24 (s, 1H), 7.83 (s, 1H), 7.71 (d, *J* = 8.0 Hz, 1H), 7.67 (d, *J* = 8.0 Hz, 1H), 4.12 (s, 2H), 1.87 (s, 3H); ^{13}C NMR (100 MHz, DMSO-*d6*) *δ* 154.5, 151.7, 144.8, 138.4, 135.3, 131.7, 131.5, 130.8, 127.8, 127.5, 52.1, 19.9; HRMS(ESI) m/z $[M+H]^+$ calcd. for $C_{12}H_{13}Cl_2N_6O_2$: 343.0472, found: 343.0466.

(*E*)-2-(4-bromobenzylidene)-N-(6-methyl-3-oxo-2,3-dihydro-1,2,4-triazin-4(*5H*)-yl) hydrazinecarboxamide (**3o**). White solid, m.p. 271-272 °C, yield 82%; ^{1}H NMR (400 MHz, DMSO-*d6*) *δ* 10.86 (s, 1H), 9.79 (s, 1H), 9.33 (s, 1H), 7.84 (s, 1H), 7.76 (d, *J* = 8.0 Hz, 2H), 7.61 (d, *J* = 8.0 Hz, 2H), 4.11 (s, 2H), 1.87 (s, 3H); ^{13}C NMR (100 MHz, DMSO-*d6*) *δ* 154.6, 151.7, 144.9, 139.8, 133.8, 131.6, 128.9, 122.6, 52.2, 19.9; HRMS(ESI) m/z $[M+H]^+$ calcd. for $C_{12}H_{14}BrN_6O_2$: 353.0356, found: 353.0354.

(*E*)-2-(3-cyanobenzylidene)-N-(6-methyl-3-oxo-2,3-dihydro-1,2,4-triazin-4(*5H*)-yl) hydrazinecarboxamide (**3p**). White solid, m.p. 249-250 °C, yield 65%; ^{1}H NMR (300 MHz, DMSO-*d6*) *δ* 10.99 (s, 1H), 9.79 (s, 1H), 9.46 (s, 1H), 8.46 (s, 1H), 8.02 (d, *J* = 10.4 Hz, 1H), 7.88 (s, 1H), 7.81 (d, *J* = 10.4 Hz, 1H), 7.61 (t, *J* = 10.4 Hz, 1H), 4.13 (s, 2H), 1.88 (s, 3H); ^{13}C NMR (100 MHz, DMSO-*d6*) *δ* 154.5, 151.7, 144.9, 138.6, 135.8, 132.3, 131.9, 129.8, 129.7, 118.7, 111.9, 52.1, 19.9; HRMS(ESI) m/z $[M+H]^+$ calcd. for $C_{13}H_{14}N_7O_2$: 300.1203, found: 300.1210.

(*E*)-2-(4-cyanobenzylidene)-N-(6-methyl-3-oxo-2,3-dihydro-1,2,4-triazin-4(5*H*)-yl)

hydrazinecarboxamide (**3q**). White solid, m.p. 293-294 °C, yield 78%; ^{1}H NMR (400 MHz, DMSO-d_6) δ 11.06 (s, 1H), 9.80 (s, 1H), 9.44 (s, 1H), 8.00 (d, J = 7.2 Hz, 2H), 7.90 (s, 1H), 7.88 (d, J = 7.2 Hz, 2H), 4.12 (s, 2H), 1.87 (s, 3H); ^{13}C NMR (100 MHz, DMSO-d_6) δ 154.5, 151.6, 144.8, 139.1, 139.0, 132.5, 127.5, 118.8, 111.2, 52.1, 19.9; HRMS(ESI) m/z [M+H]$^+$ calcd. for $C_{13}H_{14}N_7O_2$: 300.1203, found: 300.1206.

(*E*)-N-(6-methyl-3-oxo-2,3-dihydro-1,2,4-triazin-4(*5H*)-yl)-2-(4-(trifluoromethyl)benzylidene)hydrazinecarboxamide (**3r**). White solid, m.p. 239-240 °C, yield 76%; ^{1}H NMR (400 MHz, DMSO-d_6) δ 10.99 (s, 1H), 9.79 (s, 1H), 9.40 (s, 1H), 8.02 (d, J = 8.0 Hz, 2H), 7.94 (s, 1H), 7.76 (d, J = 8.0 Hz, 2H), 4.13 (s, 2H), 1.88 (s, 3H); ^{13}C NMR (100 MHz, DMSO-d_6) δ 154.5, 151.7, 144.9, 139.4, 138.5, 129.1 (q, J = 252 Hz), 127.4, 125.5, 122.9, 52.2, 19.9; HRMS(ESI) m/z [M+H]$^+$ calcd. for $C_{13}H_{14}F_3N_6O_2$: 343.1125, found: 343.1130.

(*E*)-N-(6-methyl-3-oxo-2,3-dihydro-1,2,4-triazin-4(*5H*)-yl)-2-(4-(trifluoromethoxy)benzylidene)hydrazinecarboxamide (**3s**). White solid, m.p. 266-267 °C, yield 87%; ^{1}H NMR (400 MHz, DMSO-d_6) δ 10.86 (s, 1H), 9.78 (s, 1H), 9.32 (s, 1H), 7.93 (d, J = 8.4 Hz, 2H), 7.89 (s, 1H), 7.40 (d, J = 8.4 Hz, 2H), 4.12 (s, 2H), 1.87 (s, 3H); ^{13}C NMR (100 MHz, DMSO-d_6) δ 154.7, 151.8, 148.9, 144.9, 139.5, 133.9, 128.8, 121.2, 120.1 (q, J = 254.8 Hz), 52.2, 19.9. HRMS(ESI) m/z [M+H]$^+$ calcd. for $C_{13}H_{14}F_3N_6O_3$: 359.1074, found: 359.1081.

(*E*)-2-(4-(dimethylamino)benzylidene)-N-(6-methyl-3-oxo-2,3-dihydro-1,2,4-triazin-4(*5H*)-yl)hydrazinecarboxamide (**3t**). Pink solid, m.p. 229-230 °C, yield 69%; ^{1}H NMR (400 MHz, DMSO-d_6) δ 10.46 (s, 1H), 9.76 (s, 1H), 9.06 (s, 1H), 7.73 (s, 1H), 7.57 (d, J

= 8.4 Hz, 2H), 6.70 (d, *J* = 8.4 Hz, 2H), 4.11 (s, 2H), 2.95 (s, 6H), 1.87 (s, 3H); ^{13}C NMR (100 MHz, DMSO-*d*$_6$) *δ* 154.7, 151.7, 151.1, 144.8, 141.9, 128.1, 121.9, 111.7, 52.2, 39.8, 19.8; HRMS(ESI) m/z $[M+H]^+$ calcd. for $C_{14}H_{20}N_7O_2$: 318.1673, found: 318.1677.

(*E*)-2-((6-methoxynaphthalen-2-yl)methylene)-N-(6-methyl-3-oxo-2,3-dihydro-1,2,4-triazin-4(*5H*)-yl)hydrazinecarboxamide (**3u**). Yellow solid, m.p. 232-233 °C, yield 79 %; ^{1}H NMR (300 MHz, DMSO-*d*$_6$) *δ* 10.79 (s, 1H), 9.79 (s, 1H), 9.29 (s, 1H), 8.11 (d, *J* = 8.4 Hz, 1H), 8.02 (s, 1H), 7.99 (s, 1H), 7.84 (m, 2H), 7.36 (s, 1H), 7.20 (d, *J* = 8.4 Hz, 1H), 4.14 (s, 2H), 3.89 (s, 3H), 1.88 (s, 3H); ^{13}C NMR (100 MHz, DMSO-*d*$_6$) *δ* 158.0, 154.7, 151.7, 144.8, 141.3, 135.0, 130.0, 129.7, 128.2, 127.8, 127.1, 123.6, 119.0, 106.4, 55.3, 52.2, 19.8; HRMS(ESI) m/z $[M+H]^+$ calcd. for $C_{17}H_{19}N_6O_3$: 355.1513, found: 355.1516.

(*E*)-N-(6-methyl-3-oxo-2,3-dihydro-1,2,4-triazin-4(*5H*)-yl)-2-(pyridin-4-ylmethylene)hydrazinecarboxamide (**3v**). White solid, m.p. 289-290 °C, yield 80%; ^{1}H NMR (400 MHz, DMSO-*d*$_6$) *δ* 11.10 (s, 1H), 9.81 (s, 1H), 9.44 (s, 1H), 8.59 (m, 1H), 7.84 (s, 1H), 7.76 (m, 1H), 4.12 (s, 2H), 1.87 (s, 3H); ^{13}C NMR (100 MHz, DMSO-*d*$_6$) *δ* 154.4, 151.7, 150.0, 144.9, 141.6, 138.5, 120.9, 52.1, 19.9; HRMS(ESI) m/z $[M+H]^+$ calcd. for $C_{11}H_{14}N_7O_2$: 276.1203, found: 276.1205.

(*E*)-N-(6-methyl-3-oxo-2,3-dihydro-1,2,4-triazin-4(*5H*)-yl)-2-(thiophen-2-ylmethylene)hydrazinecarboxamide (**3w**). Yellow solid, m.p. 226-227 °C, yield 68%; ^{1}H NMR (400 MHz, DMSO-*d*$_6$) *δ* 10.73 (s, 1H), 9.76 (s, 1H), 8.89 (s, 1H), 8.09 (s, 1H), 7.62 (d, *J* = 4.8 Hz, 1H), 7.39 (d, *J* = 3.2 Hz, 1H), 7.10 (dd, *J* = 3.6, 4.8 Hz, 1H), 4.10 (s, 2H), 1.86 (s, 3H); ^{13}C NMR (100 MHz, DMSO-*d*$_6$) *δ* 154.3, 151.7, 144.9, 138.9, 136.8, 129.7,

128.2, 127.8, 52.1, 19.9; HRMS(ESI) m/z $[M+H]^+$ calcd. for $C_{10}H_{13}N_6O_2S$: 281.0815, found: 281.0815.

The general preparation process of 5a-5u. To a solution of compound **1** (0.74g, 4 mmol) and *p*-toluene sulfonic acid (0.14g, 0.80 mmol)in methanol (300 mL) was added the corresponding ketones **4** (4 mmol), and then the mixture was refluxed for 6 h. The solution was cooled to room temperature, and evaporated under reduced pressure to remove part of methanol until a large amount of solid precipitates. The resulting precipitate was collected by filtration in vacuo and was washed with methanol to provide compounds **5a-5u**.

(*E*)-2-(butan-2-ylidene)-N-(6-methyl-3-oxo-2,3-dihydro-1,2,4-triazin-4(*5H*)-yl)hydrazinecarboxamide (**5a**). White solid, m.p. 225-226 °C, yield 65%; ^{1}H NMR (400 MHz, DMSO-d_6) δ 9.70 (s, 1H), 9.41 (s, 1H), 8.62 (s, 1H), 4.07 (s, 2H), 2.20 (q, *J* = 7.6 Hz, 2H), 1.85 (s, 3H), 1.79 (s, 3H), 1.03 (t, *J* = 7.6 Hz, 3H); ^{13}C NMR (100 MHz, DMSO-d_6) δ 155.3, 152.5, 151.6, 144.8, 52.2, 31.4, 19.9, 15.5, 10.8; HRMS(ESI) m/z $[M+H]^+$ calcd. for $C_9H_{17}N_6O_2$: 241.1408, found: 241.1410.

2-cyclopentylidene-N-(6-methyl-3-oxo-2,3-dihydro-1,2,4-triazin-4(*5H*)-yl)hydrazinecarboxamide (**5b**). White solid, m.p. 227-229 °C, yield 68%; ^{1}H NMR (400 MHz, DMSO-d_6) δ 9.70 (s, 1H), 9.33 (s, 1H), 8.66 (s, 1H), 4.05 (s, 2H), 2.29 (t, *J* = 7.2 Hz, 2H), 2.22 (t, *J* = 7.2 Hz, 2H), 1.84 (s, 3H), 1.77–1.72 (m, 2H), 1.69–1.64 (m, 2H); ^{13}C NMR (100 MHz, DMSO-d_6) δ 160.7, 155.2, 151.6, 144.7, 52.2, 32.8, 28.0, 24.5, 19.9; HRMS(ESI) m/z $[M+H]^+$ calcd. for $C_{10}H_{17}N_6O_2$: 253.1408, found: 253.1413.

2-cyclohexylidene-N-(6-methyl-3-oxo-2,3-dihydro-1,2,4-triazin-4(*5H*)-yl)hydrazine

carboxamide (**5c**). White solid, m.p. 233-234 °C, yield 56%; ^{1}H NMR (400 MHz, DMSO-d_6) δ 9.70 (s, 1H), 9.60 (s, 1H), 8.69 (s, 1H), 4.07 (s, 2H), 2.31 (m, 2H), 2.21 (t, J = 6.4 Hz, 2H), 1.84 (s, 3H), 1.61 (brs, 2H), 1.54 (brs, 4H); ^{13}C NMR (100 MHz, DMSO-d_6) δ 155.5, 153.8, 151.6, 144.7, 52.2, 34.8, 26.8, 26.3, 25.6, 25.2, 19.9; HRMS(ESI) m/z [M+H]$^+$ calcd. for $C_{11}H_{19}N_6O_2$: 267.1564, found: 267.1567.

(*E*)-N-(6-methyl-3-oxo-2,3-dihydro-1,2,4-triazin-4(*5H*)-yl)-2-(1-phenylethylidene)hydrazinecarboxamide (**5d**). White solid, m.p. 249-250 °C, yield 66%; ^{1}H NMR (400 MHz, DMSO-d_6) δ 9.89 (s, 1H), 9.76 (s, 1H), 9.13 (s, 1H), 7.91 (d, J = 7.6 Hz, 2H), 7.39-7.37 (m, 3H), 4.13 (s, 2H), 2.21 (s, 3H), 1.87 (s, 3H); ^{13}C NMR (100 MHz, DMSO-d_6) δ 155.2, 151.7, 146.0, 144.8, 137.8, 128.8, 128.2, 126.3, 52.2, 19.9, 13.3; HRMS(ESI) m/z [M+H]$^+$ calcd. for $C_{13}H_{17}N_6O_2$: 289.1408, found: 289.1407.

(*E*)-N-(6-methyl-3-oxo-2,3-dihydro-1,2,4-triazin-4(*5H*)-yl)-2-(1-phenylpropylidene) hydrazinecarboxamide (**5e**). White solid, m.p. 232-233 °C, yield 68%; ^{1}H NMR (400 MHz, DMSO-d_6) δ 10.03 (s, 1H), 9.76 (s, 1H), 9.11 (s, 1H), 7.91 (d, J = 7.2 Hz, 2H), 7.39-7.37 (d, J = 6.8 Hz, 3H), 4.13 (s, 2H), 2.76 (q, J = 7.2 Hz, 2H), 1.87 (s, 3H), 1.01 (t, J = 7.2 Hz, 3H); ^{13}C NMR (100 MHz, DMSO-d_6) δ 155.2, 151.7, 150.0, 144.8, 136.6, 128.8, 128.3, 126.3, 52.2, 19.9, 18.7, 10.7; HRMS(ESI) m/z [M+H]$^+$ calcd. for $C_{14}H_{19}N_6O_2$: 303.1564, found: 303.1565.

(*E*)-N-(6-methyl-3-oxo-2,3-dihydro-1,2,4-triazin-4(*5H*)-yl)-2-(1-phenylbutylidene)hydrazinecarboxamide (**5f**). White solid, m.p. 221-222 °C, yield 60%; ^{1}H NMR (300 MHz, DMSO-d_6) δ 10.07 (s, 1H), 9.76 (s, 1H), 9.10 (s, 1H), 7.90 (m, 2H), 7.38 (m, 3H), 4.13 (s, 2H), 2.73(t, J = 10.0 Hz, 2H), 1.87 (s, 3H), 1.43 (m, 2H), 0.95 (t, J = 9.2 Hz, 3H); ^{13}C

NMR (100 MHz, DMSO-d_6) δ 155.3, 151.7, 148.9, 144.8, 136.9, 128.8, 128.3, 126.4, 52.2, 27.1, 19.9, 19.4, 13.8; HRMS(ESI) m/z $[M+H]^+$ calcd. for $C_{15}H_{21}N_6O_2$: 317.1721, found: 317.1724.

(*E*)-N-(6-methyl-3-oxo-2,3-dihydro-1,2,4-triazin-4(*5H*)-yl)-2-(1-p-tolylethylidene)hydrazinecarboxamide (**5g**). White solid, m.p. 271-273 °C, yield 87%; ^{1}H NMR (400 MHz, DMSO-d_6) δ 9.83 (s, 1H), 9.76 (s, 1H), 9.10 (s, 1H), 7.81 (d, *J* = 7.6 Hz, 2H), 7.19 (d, *J* = 7.6 Hz, 2H), 4.12 (s, 2H), 2.32 (s, 3H), 2.18 (s, 3H), 1.87 (s, 3H); ^{13}C NMR (100 MHz, DMSO-d_6) δ 155.3, 151.7, 146.0, 144.8, 138.3, 135.1, 128.8, 126.2, 52.2, 20.9, 19.9, 13.2; HRMS(ESI) m/z $[M+H]^+$ calcd. for $C_{14}H_{19}N_6O_2$: 303.1564, found: 303.1561.

(*E*)-2-(1-(3-methoxyphenyl)ethylidene)-N-(6-methyl-3-oxo-2,3-dihydro-1,2,4-triazin-4(*5H*)-yl)hydrazinecarboxamide (**5h**). White solid, m.p. 228-229 °C, yield 86%; ^{1}H NMR (400 MHz, DMSO-d_6) δ 9.85 (s, 1H), 9.75 (s, 1H), 9.13 (s, 1H), 7.47–7.43 (m, 2H), 7.30 (t, *J* = 8.0 Hz, 1H), 6.94 (d, *J* = 8.0 Hz,, 1H), 4.12 (s, 2H), 3.80 (s, 3H), 2.19 (s, 3H), 1.87 (s, 3H); ^{13}C NMR (100 MHz, DMSO-d_6) δ 159.3, 155.2, 151.7, 146.1, 144.9, 139.4, 129.3, 118.9, 114.3, 111.9, 55.3, 52.2, 19.9, 13.6; HRMS(ESI) m/z $[M+H]^+$ calcd. for $C_{14}H_{19}N_6O_3$: 319.1513, found: 319.1517.

(*E*)-2-(1-(4-methoxyphenyl)ethylidene)-N-(6-methyl-3-oxo-2,3-dihydro-1,2,4-triazin-4(*5H*)-yl)hydrazinecarboxamide (**5i**). White solid, m.p. 249-250 °C, yield 77%; ^{1}H NMR (400 MHz, DMSO-d_6) δ 9.76 (s, 2H), 9.09 (s, 1H), 7.86 (d, *J* = 8.4 Hz, 2H), 6.92 (d, *J* = 8.4 Hz, 2H), 4.12 (s, 2H), 3.78 (s, 3H), 2.17 (s, 3H), 1.87 (s, 3H); ^{13}C NMR (100 MHz, DMSO-d_6) δ 159.9, 155.3, 151.7, 145.9, 144.8, 130.4, 127.8, 113.5, 55.2, 52.2, 19.9, 13.2; HRMS(ESI) m/z $[M+H]^+$ calcd. for $C_{14}H_{19}N_6O_3$: 319.1513, found: 319.1518.

(*E*)-2-(1-(3,4-dimethoxyphenyl)ethylidene)-N-(6-methyl-3-oxo-2,3-dihydro-1,2,4-triazin-4(*5H*)-yl)hydrazinecarboxamide (**5j**). White solid, m.p. 226-227 °C, yield 86%; ^{1}H NMR (400 MHz, DMSO-*d6*) *δ* 9.76 (s, 1H), 9.73(s, 1H), 9.12 (s, 1H), 7.51 (s, 1H), 7.33 (d, *J* = 8.0 Hz, 1H), 6.93 (d, *J* = 8.0 Hz, 1H), 4.14 (s, 2H), 3.83(s, 3H), 3.78 (s, 3H), 2.18 (s, 3H), 1.87 (s, 3H); ^{13}C NMR (100 MHz, DMSO-*d6*) *δ* 155.3, 151.8, 149.9, 148.6, 146.3, 144.8, 130.6, 119.6, 110.9, 109.7, 55.9, 55.5, 52.2, 19.9, 13.5; HRMS(ESI) m/z $[M+H]^+$ calcd. for $C_{15}H_{21}N_6O_4$: 349.1619, found: 349.1622.

(*E*)-N-(6-methyl-3-oxo-2,3-dihydro-1,2,4-triazin-4(*5H*)-yl)-2-(1-(3,4,5-trimethoxyphenyl)ethylidene)hydrazinecarboxamide (**5k**). White solid, m.p. 260-261 °C, yield 85%; ^{1}H NMR (400 MHz, DMSO-*d6*) *δ* 9.79 (s, 1H), 9.77(s, 1H), 9.15 (s, 1H), 7.10 (s, 2H), 4.14 (s, 2H), 3.84 (s, 6H), 3.67 (s, 3H), 2.21 (s, 3H), 1.87 (s, 3H); ^{13}C NMR (100 MHz, DMSO-*d6*) *δ* 155.2, 152.7, 151.8, 146.4, 144.8, 138.5, 133.6, 104.2, 60.1, 56.2, 52.2, 19.9, 13.8; HRMS(ESI) m/z $[M+H]^+$ calcd. for $C_{16}H_{23}N_6O_5$: 379.1724, found: 379.1724.

(*E*)-2-(1-(4-hydroxyphenyl)ethylidene)-N-(6-methyl-3-oxo-2,3-dihydro-1,2,4-triazin-4(*5H*)-yl)hydrazinecarboxamide (**5l**). White solid, m.p. 237-238 °C, yield 75%; ^{1}H NMR (300 MHz, DMSO-*d6*) *δ* 9.74 (s, 1H), 9.70 (s, 1H), 9.66 (s, 1H), 9.02 (s, 1H), 7.75 (d, *J* = 11.6 Hz, 2H), 6.75 (d, *J* = 11.6 Hz, 2H), 4.12 (s, 2H), 2.14 (s, 3H), 1.87 (s, 3H); ^{13}C NMR (100 MHz, DMSO-*d6*) *δ* 158.4, 155.4, 151.8, 146.4, 144.9, 128.9, 127.9, 115.0, 52.3, 19.9, 13.2; HRMS(ESI) m/z $[M+H]^+$ calcd. for $C_{13}H_{17}N_6O_3$: 305.1357, found: 305.1357.

(*E*)-2-(1-(4-fluorophenyl)ethylidene)-N-(6-methyl-3-oxo-2,3-dihydro-1,2,4-triazin-4(*5H*)-yl)hydrazinecarboxamide (**5m**). White solid, m.p. 272-273 °C, yield 88%; ^{1}H NMR

(400 MHz, DMSO-d_6) δ 9.90 (s, 1H), 9.77 (s, 1H), 9.18 (s, 1H), 7.98 (m, 2H), 7.20 (m, 2H), 4.12 (s, 2H), 2.20 (s, 3H), 1.87 (s, 3H); ^{13}C NMR (100 MHz, DMSO-d_6) δ 162.6 (d, J = 245.0 Hz), 155.2, 151.7, 145.1, 144.8, 134.3 (d, J = 2.0 Hz), 128.5 (d, J = 8.0 Hz), 115.0 (d, J = 21.0 Hz), 52.2, 19.9, 13.3; HRMS(ESI) m/z [M+H]$^+$ calcd. for $C_{13}H_{16}FN_6O_2$: 307.1313, found: 307.1316.

(*E*)-2-(1-(3-chlorophenyl)ethylidene)-N-(6-methyl-3-oxo-2,3-dihydro-1,2,4-triazin-4(*5H*)-yl)hydrazinecarboxamide (**5n**). White solid, m.p. 274-275 °C, yield 86%; ^{1}H NMR (400 MHz, DMSO-d_6) δ 9.96 (s, 1H), 9.76 (s, 1H), 9.29 (s, 1H), 8.07 (s, 1H), 7.80 (s, 1H), 7.41 (m, 2H), 4.13 (s, 2H), 2.20 (s, 3H), 1.88 (s, 3H); ^{13}C NMR (100 MHz, DMSO-d_6) δ 155.1, 151.7, 144.8, 144.7, 139.9, 133.5, 130.0, 128.6, 125.8, 125.0, 52.1, 19.9, 13.3; HRMS(ESI) m/z [M+H]$^+$ calcd. for $C_{13}H_{16}ClN_6O_2$: 323.1018, found: 323.1020.

(*E*)-2-(1-(4-chlorophenyl)ethylidene)-N-(6-methyl-3-oxo-2,3-dihydro-1,2,4-triazin-4(5*H*)-yl)hydrazinecarboxamide (**5o**). White solid, m.p. 268-269 °C, yield 89%; ^{1}H NMR (400 MHz, DMSO-d_6) δ 9.96 (s, 1H), 9.77 (s, 1H), 9.21 (s, 1H), 7.96 (d, J = 8.0 Hz, 2H), 7.43 (d, J = 8.0 Hz, 2H), 4.12 (s, 2H), 2.19 (s, 3H), 1.87 (s, 3H); ^{13}C NMR (100 MHz, DMSO-d_6) δ 155.1, 151.7, 144.8, 136.7, 133.5, 128.1, 52.1, 19.9, 13.2; HRMS(ESI) m/z [M+H]$^+$ calcd. for $C_{13}H_{16}ClN_6O_2$: 323.1018, found: 323.1022.

(*E*)-2-(1-(2,4-dichlorophenyl)ethylidene)-N-(6-methyl-3-oxo-2,3-dihydro-1,2,4-triaz in-4(5*H*)-yl)hydrazinecarboxamide (**5p**). White solid, m.p. 252-253 °C, yield, 80%; ^{1}H NMR (400 MHz, DMSO-d_6) δ 10.02 (s, 1H), 9.74 (s, 1H), 8.92 (s, 1H), 7.68 (d, J = 1.6 Hz, 1H), 7.52–7.46 (m, 2H), 4.07 (s, 2H), 2.17 (s, 3H), 1.84 (s, 3H); ^{13}C NMR (100 MHz, DMSO-d_6) δ 155.0, 151.5, 146.1, 144.8, 137.8, 133.8, 132.4, 132.1, 129.1, 127.4, 52.1,

19.9, 17.9; HRMS(ESI) m/z $[M+H]^+$ calcd. for $C_{13}H_{15}Cl_2N_6O_2$: 357.0628, found: 357.0630.

(*E*)-N-(6-methyl-3-oxo-2,3-dihydro-1,2,4-triazin-4(5*H*)-yl)-2-(1-(2,3,4-trichlorophenyl)ethylidene)hydrazinecarboxamide (**5q**). White solid, m.p. 258-259 °C, yield 97%; ^1H NMR (400 MHz, DMSO-d_6) δ 10.05 (s, 1H), 9.71 (s, 1H), 8.90 (s, 1H), 7.70 (d, J = 8.4 Hz, 1H), 7.47 (d, J = 8.4 Hz, 1H), 4.07 (s, 2H), 2.18 (s, 3H), 1.84 (s, 3H); ^{13}C NMR (100 MHz, DMSO-d_6) δ 154.9, 151.5, 145.9, 144.7, 140.0, 132.8, 131.5, 130.5, 129.6, 128.9, 52.1, 19.9, 17.8; HRMS(ESI) m/z $[M+H]^+$ calcd. for $C_{13}H_{14}Cl_3N_6O_2$: 391.0238, found: 391.0236.

(*E*)-2-(1-(4-bromophenyl)ethylidene)-N-(6-methyl-3-oxo-2,3-dihydro-1,2,4-triazin-4(5*H*)-yl)hydrazinecarboxamide (**5r**). White solid, m.p. 265-266 °C, yield 90%; ^1H NMR (400 MHz, DMSO-d_6) δ 9.96 (s, 1H), 9.77 (s, 1H), 9.21 (s, 1H), 7.89 (d, J = 7.2 Hz, 2H), 7.56 (d, J = 7.2 Hz, 2H), 4.12 (s, 2H), 2.19 (s, 3H), 1.87 (s, 3H); ^{13}C NMR (100 MHz, DMSO-d_6) δ 155.1, 151.6, 144.9, 144.8, 137.0, 131.0, 128.4, 122.2, 52.1, 19.9, 13.1; HRMS(ESI) m/z $[M+H]^+$ calcd. for $C_{13}H_{16}BrN_6O_2$: 367.0513, found: 367.0516.

(*E*)-2-(1-(4-iodophenyl)ethylidene)-N-(6-methyl-3-oxo-2,3-dihydro-1,2,4-triazin-4(5*H*)-yl)hydrazinecarboxamide (**5s**). Yellow solid, m.p. 279-280 °C, yield 79%; ^1H NMR (400 MHz, DMSO-d_6) δ 9.93 (s, 1H), 9.76 (s, 1H), 9.17 (s, 1H), 7.73 (brs, 4H), 4.12 (s, 2H), 2.17 (s, 3H), 1.87 (s, 3H); ^{13}C NMR (100 MHz, DMSO-d_6) δ 155.1, 151.6, 144.1, 144.8, 137.4, 136.9, 128.4, 95.5, 52.1, 19.9, 13.0; HRMS(ESI) m/z $[M+H]^+$ calcd. for $C_{13}H_{16}IN_6O_2$: 415.0374, found: 415.0372.

(*E*)-N-(6-methyl-3-oxo-2,3-dihydro-1,2,4-triazin-4(5*H*)-yl)-2-(1-(4-(trifluoromethox

y)phenyl)ethylidene)hydrazinecarboxamide (**5t**). White solid, m.p. 280-281 °C, yield 86%; ^{1}H NMR (400 MHz, DMSO-d_6) δ 9.99 (s, 1H), 9.78 (s, 1H), 9.22 (s, 1H), 8.04 (brs, 2H), 7.35 (brs, 2H), 4.13 (s, 2H), 2.22 (s, 3H), 1.88 (s, 3H); ^{13}C NMR (100 MHz, DMSO-d_6) δ 155.2, 151.7, 148.6, 144.8, 144.7, 137.1, 128.3, 120.6, 120.1(q, J = 255.0 Hz), 52.1, 19.9, 13.3; HRMS(ESI) m/z $[M+H]^+$ calcd. for $C_{14}H_{16}F_3N_6O_3$: 373.1230, found: 357.1234.

(*E*)-N-(6-methyl-3-oxo-2,3-dihydro-1,2,4-triazin-4(5*H*)-yl)-2-(1-(4-nitrophenyl)ethylidene)hydrazinecarboxamide (**5u**). Yellow solid, m.p. 229-230 °C, yield 89%; ^{1}H NMR (300 MHz, DMSO-d_6) δ 10.20 (s, 1H), 9.80 (s, 1H), 9.35 (s, 1H), 8.21 (brs, 4H), 4.14 (s, 2H), 2.26 (s, 3H), 1.88 (s, 3H); ^{13}C NMR (100 MHz, DMSO-d_6) δ 154.9, 151.6, 147.2, 144.8, 144.0, 143.9, 127.4, 123.3, 52.1, 19.9, 13.2; HRMS(ESI) m/z $[M+H]^+$ calcd. for $C_{13}H_{16}N_7O_4$: 334.1258, found: 334.1258.

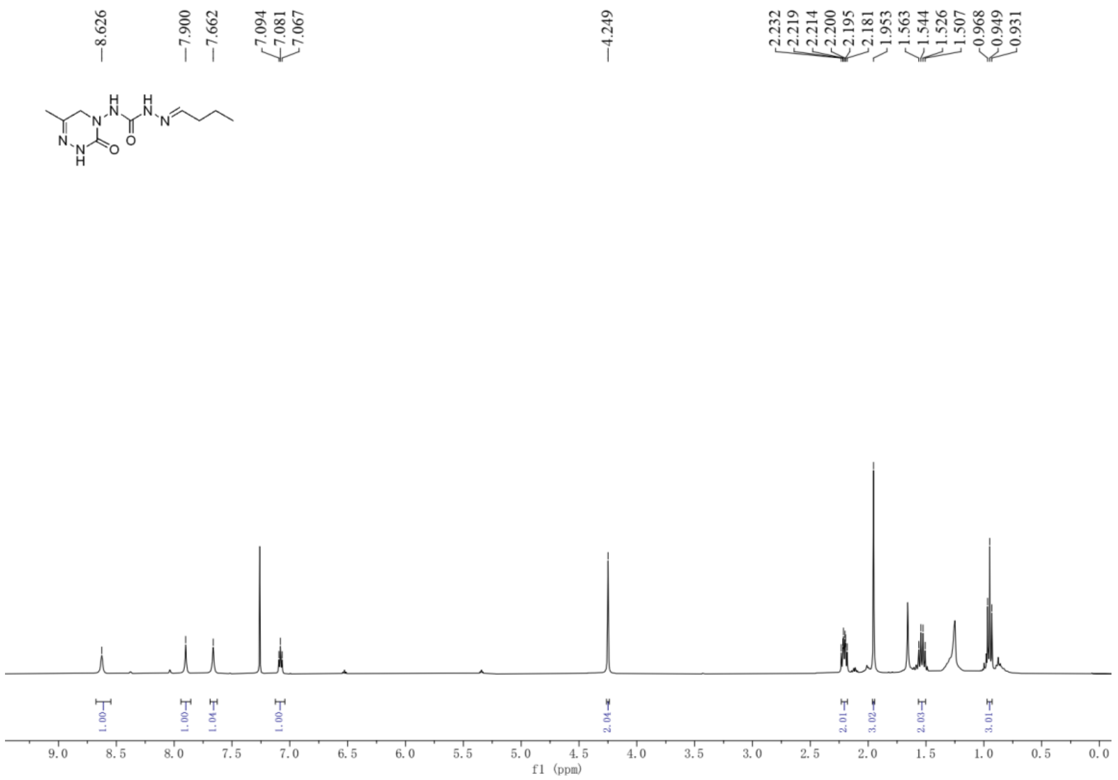

Fig. S1 ^{1}H NMR spectrum of **3a**

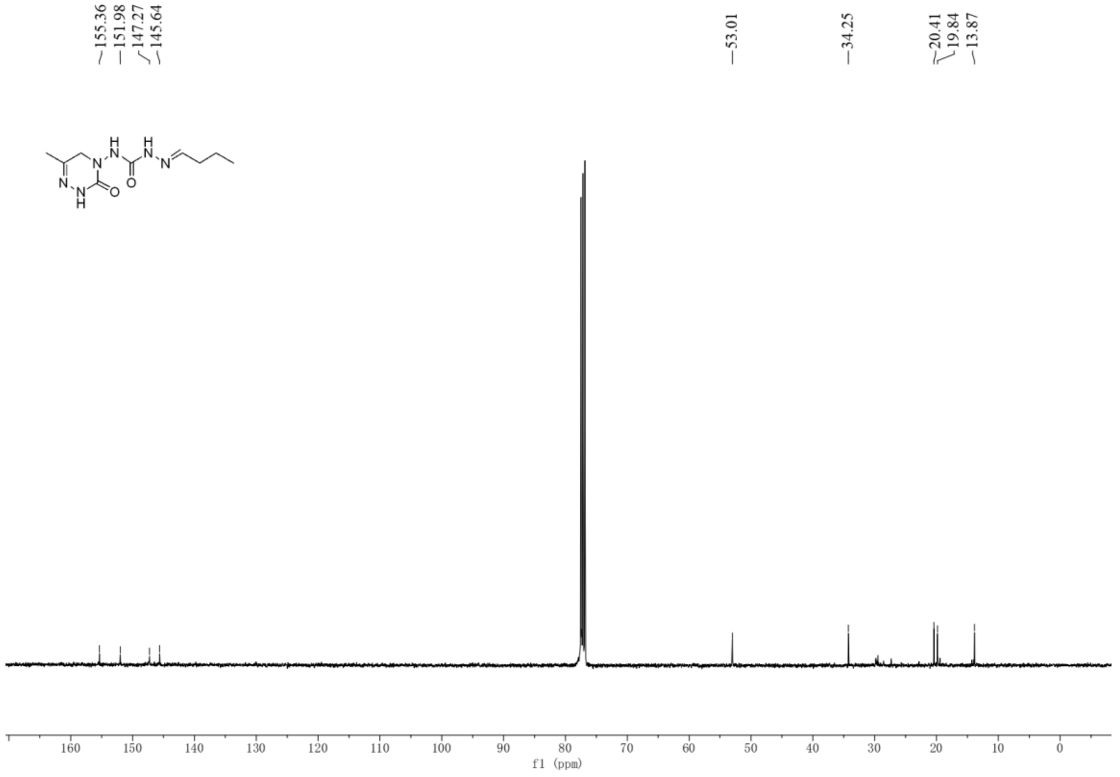

Fig. S2 ^{13}C NMR spectrum of **3a**

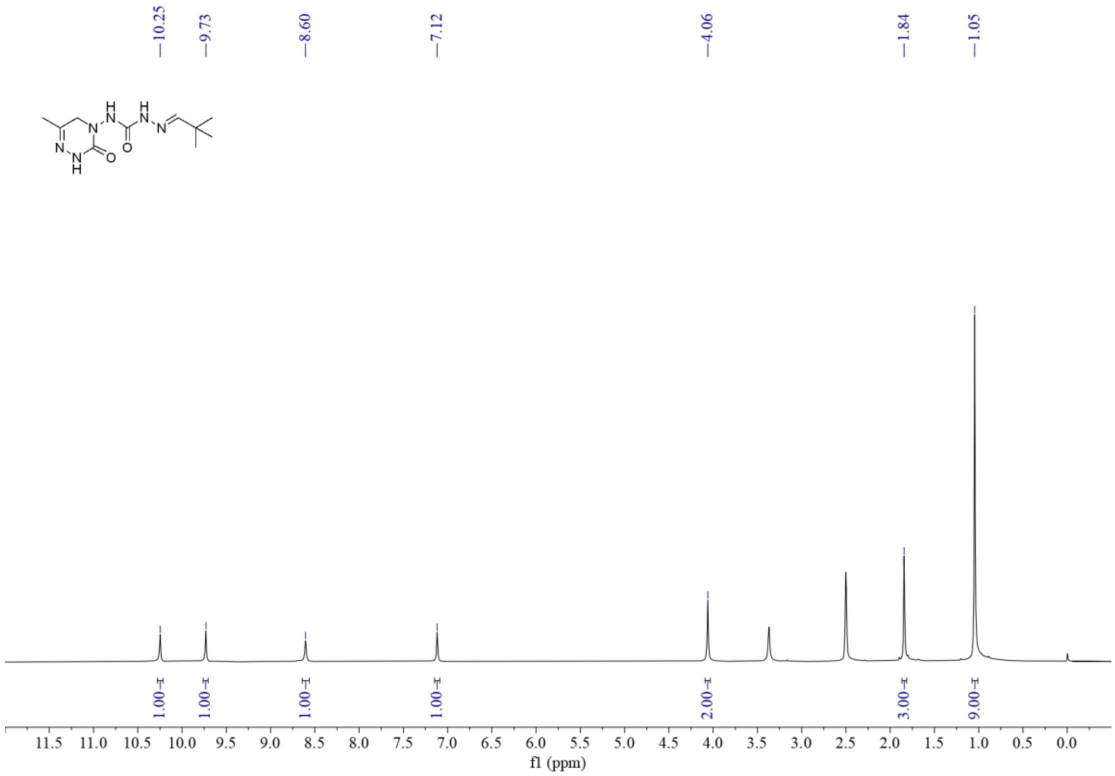

Fig. S3 ^{1}H NMR spectrum of **3b**

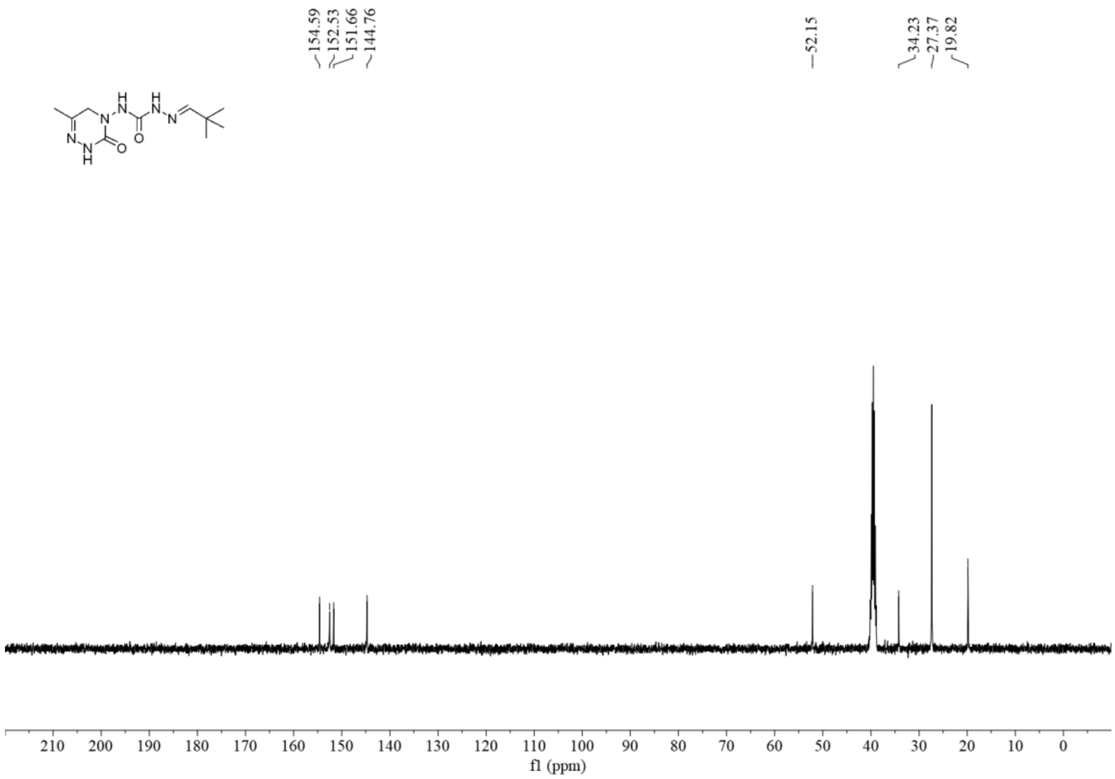

Fig. S4 ^{13}C NMR spectrum of **3b**

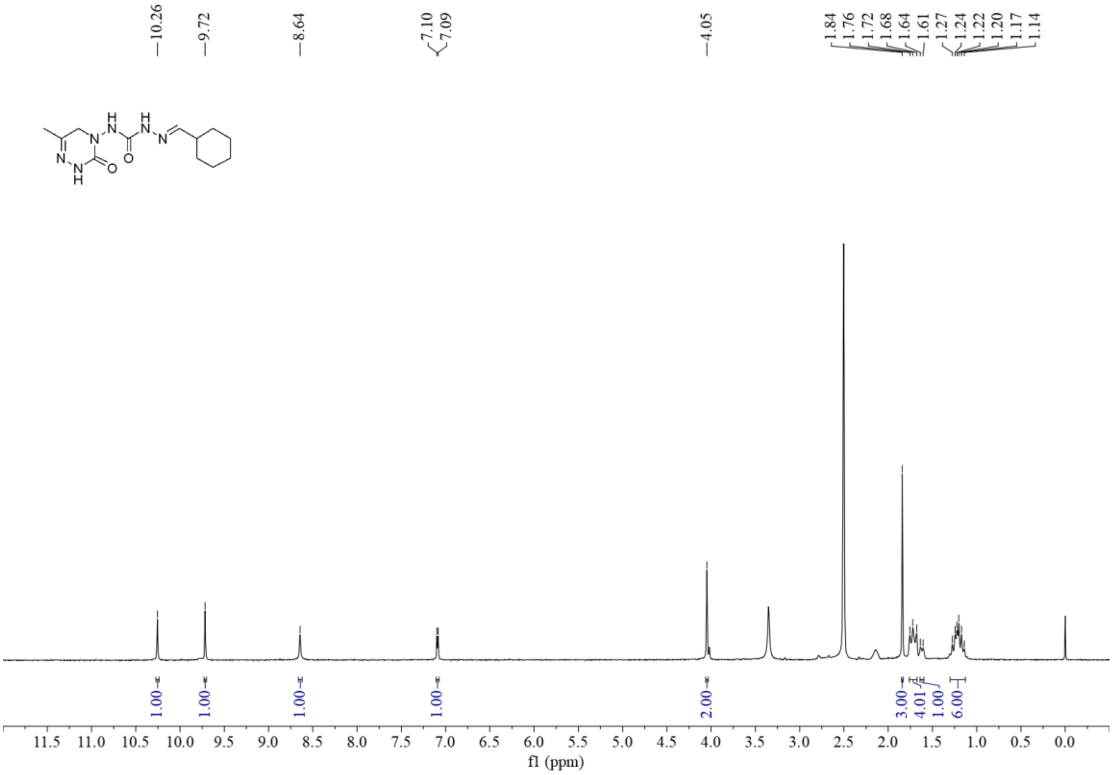

Fig. S5 ^{1}H NMR spectrum of **3c**

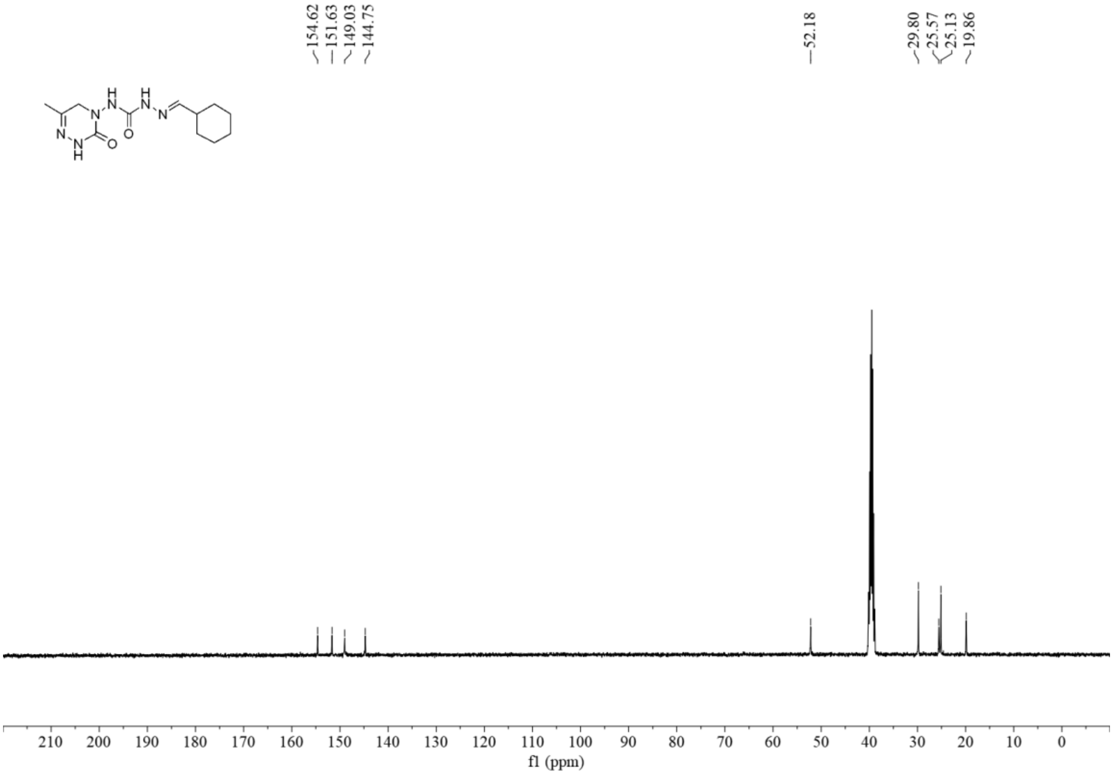

Fig. S6 ^{13}C NMR spectrum of **3c**

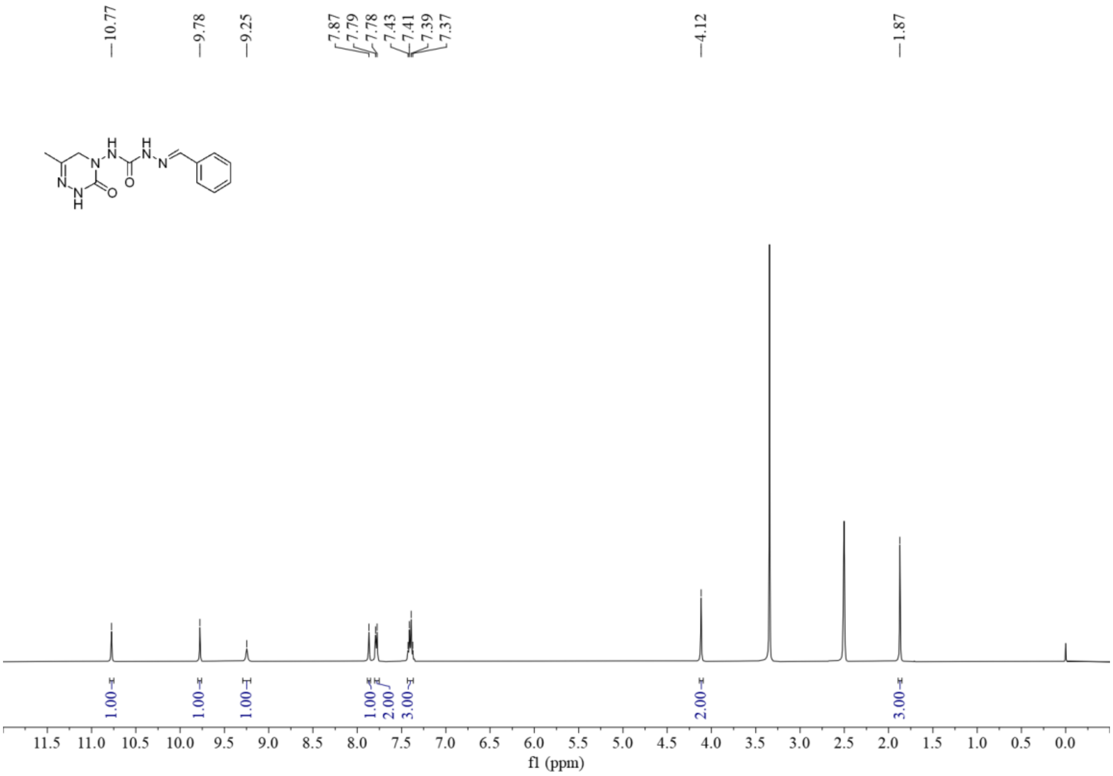

Fig. S7 ^{1}H NMR spectrum of **3d**

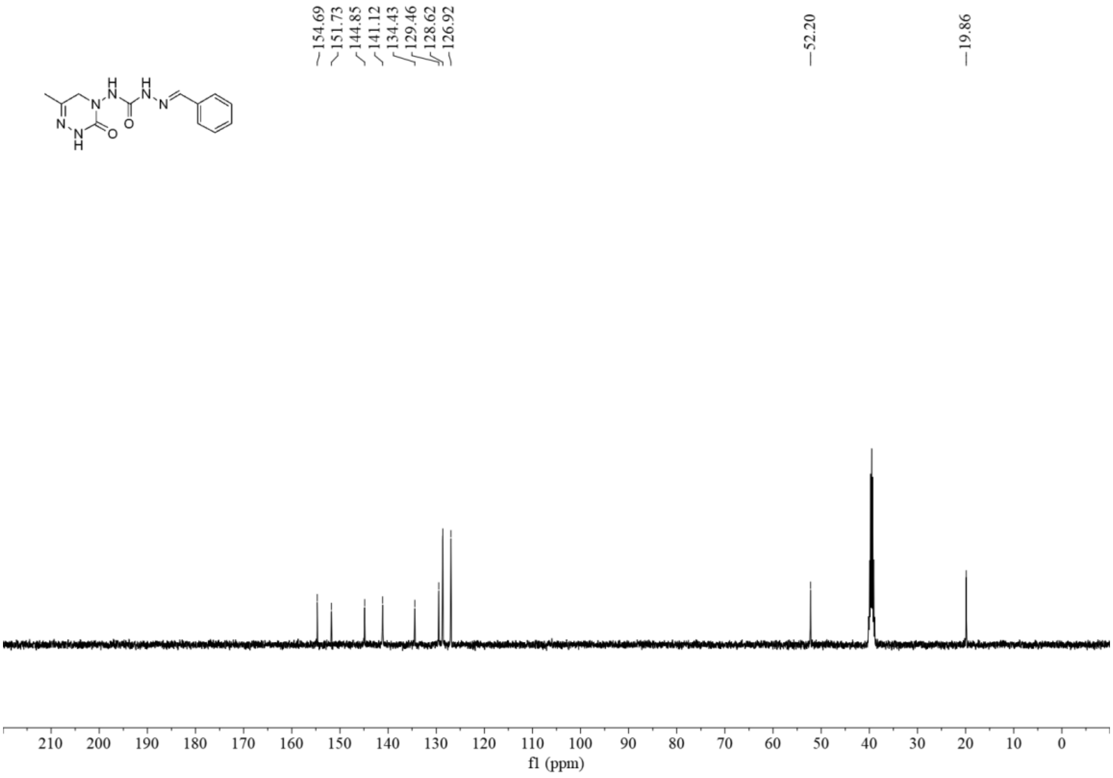

Fig. S8 ^{13}C NMR spectrum of **3d**

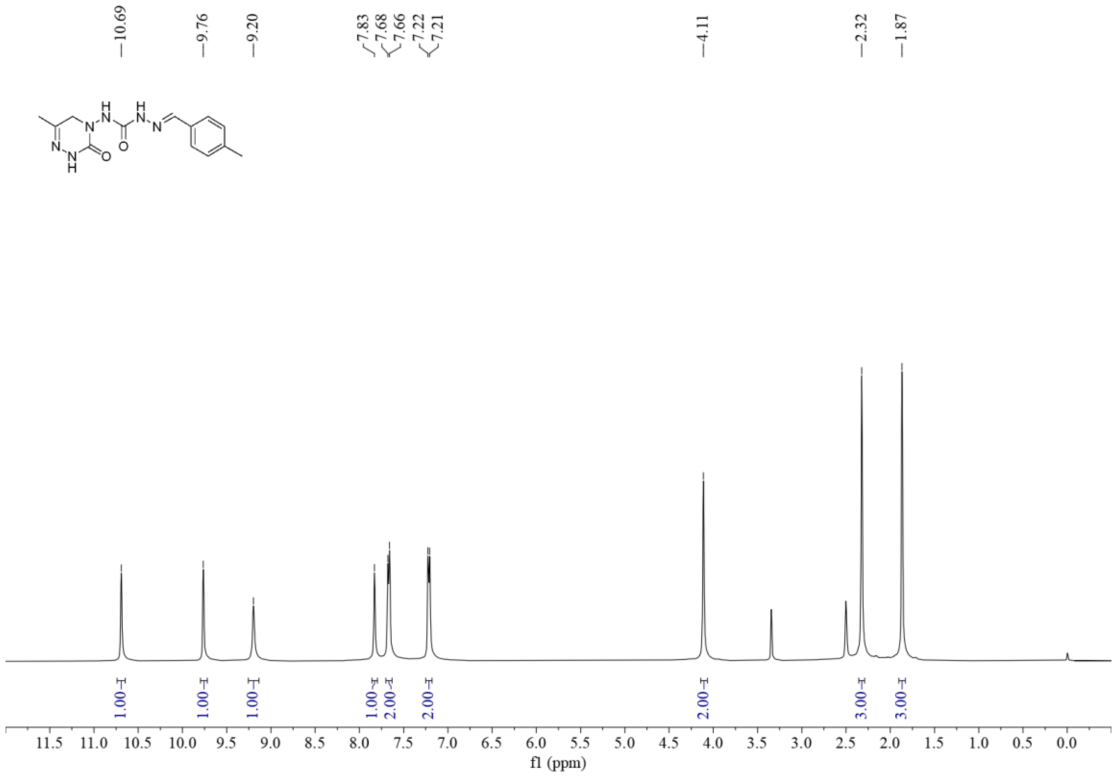

Fig. S9 ^{1}H NMR spectrum of **3e**

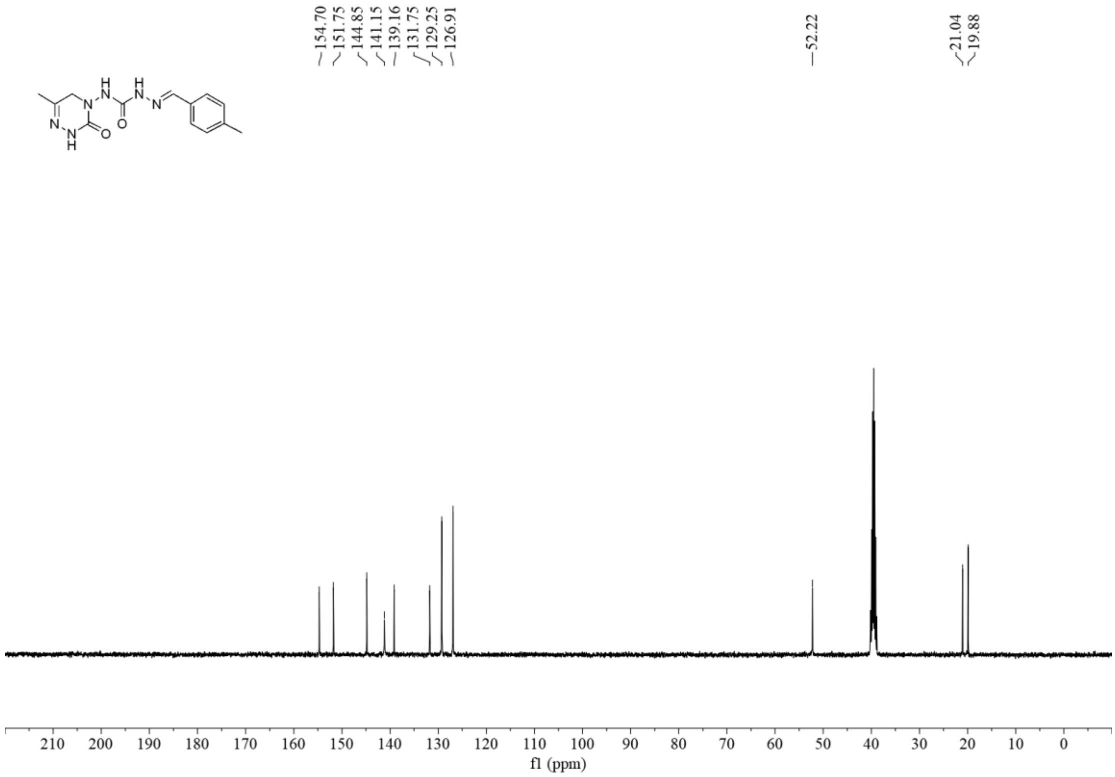

Fig. S10 ^{13}C NMR spectrum of **3e**

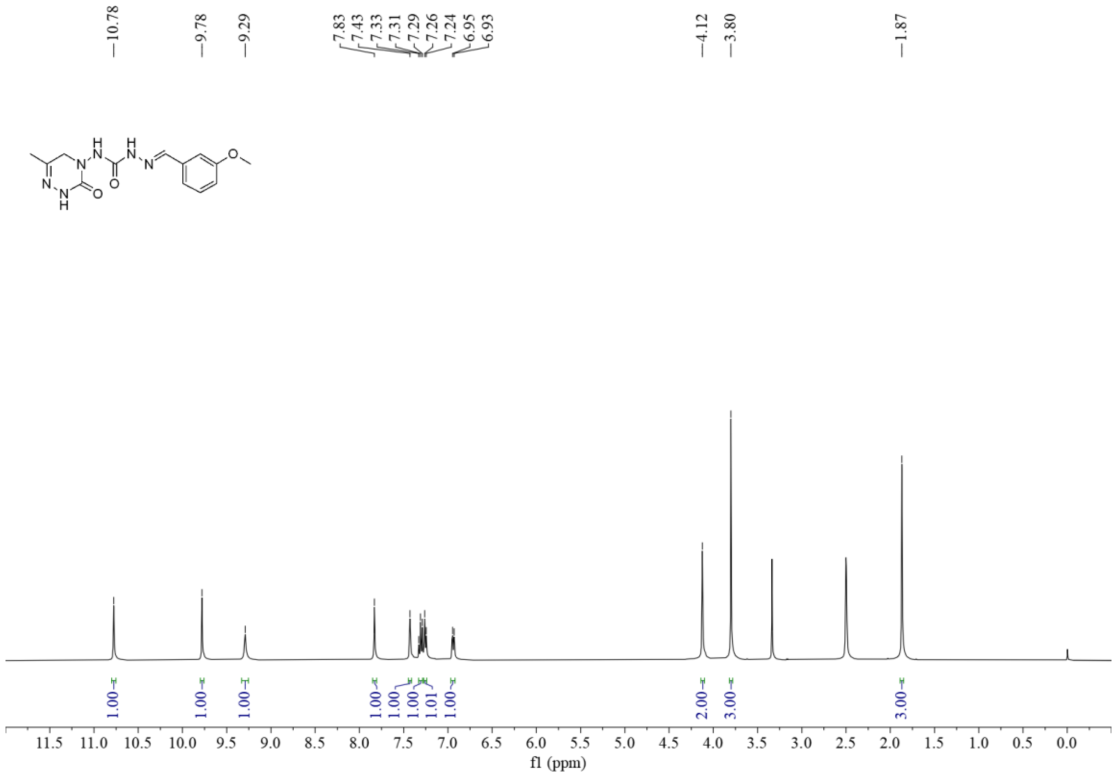

Fig. S11 ^{1}H NMR spectrum of **3f**

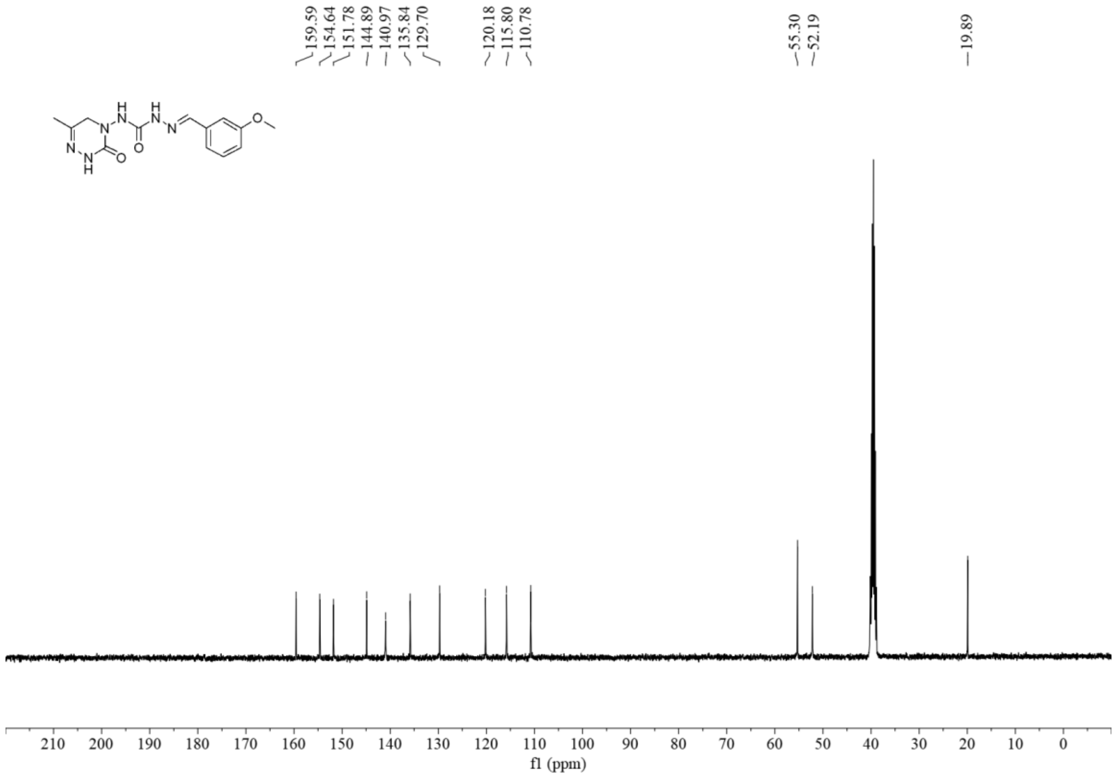

Fig. S12 ^{13}C NMR spectrum of **3f**

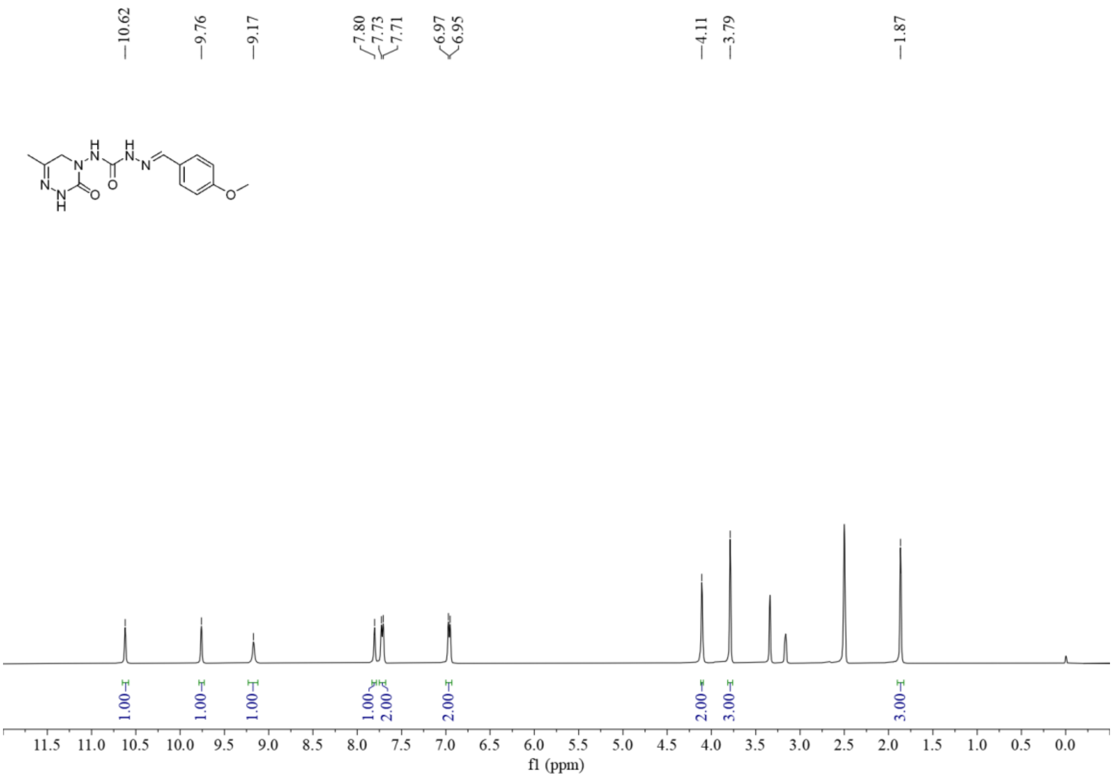

Fig. S13 ^{1}H NMR spectrum of **3g**

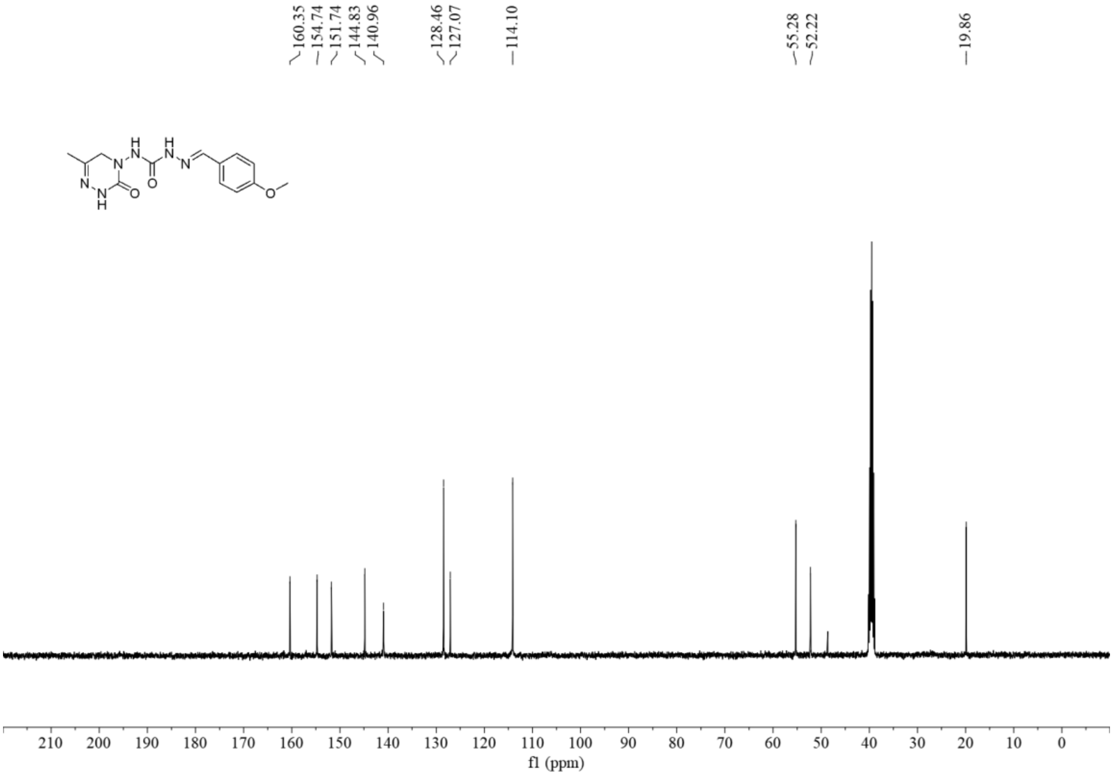

Fig. S14 ^{13}C NMR spectrum of **3g**

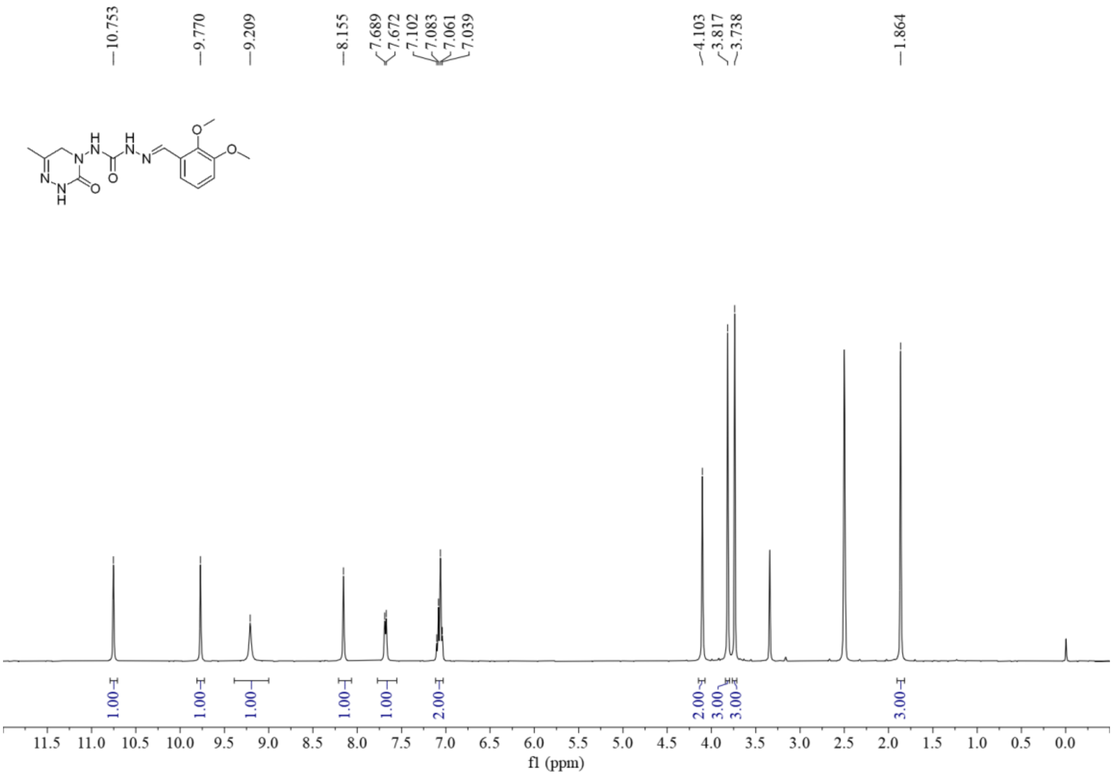

Fig. S15 ^{1}H NMR spectrum of **3h**

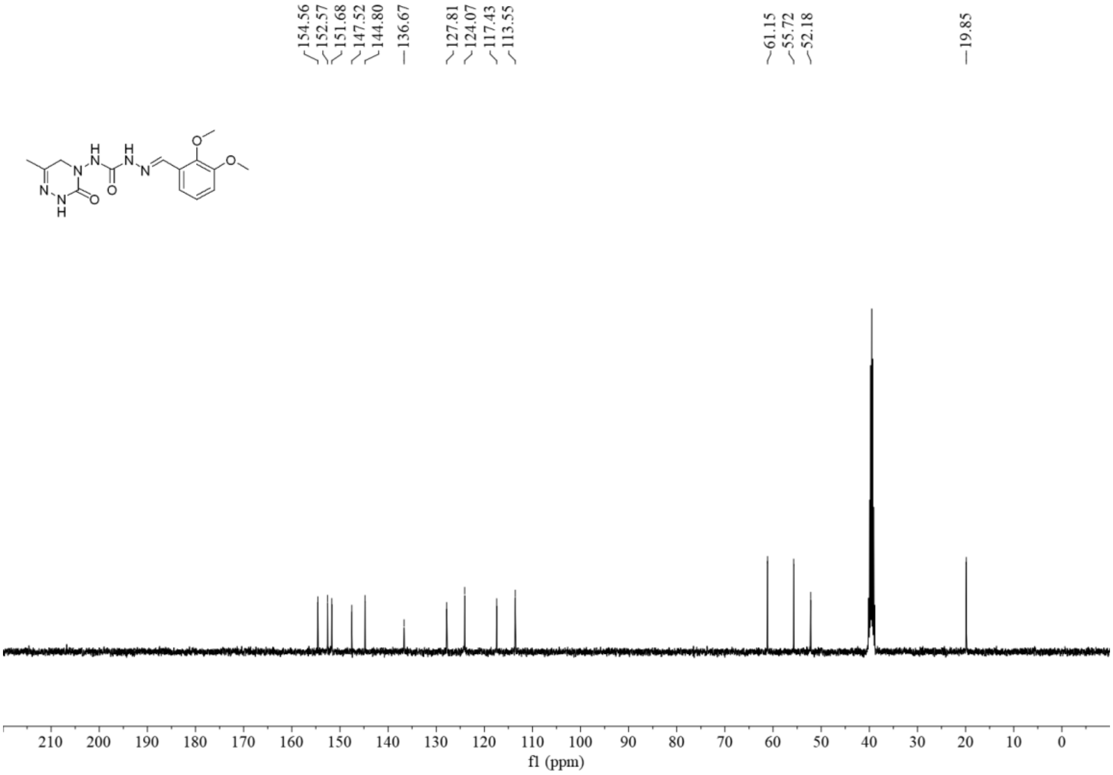

Fig. S16 ^{13}C NMR spectrum of **3h**

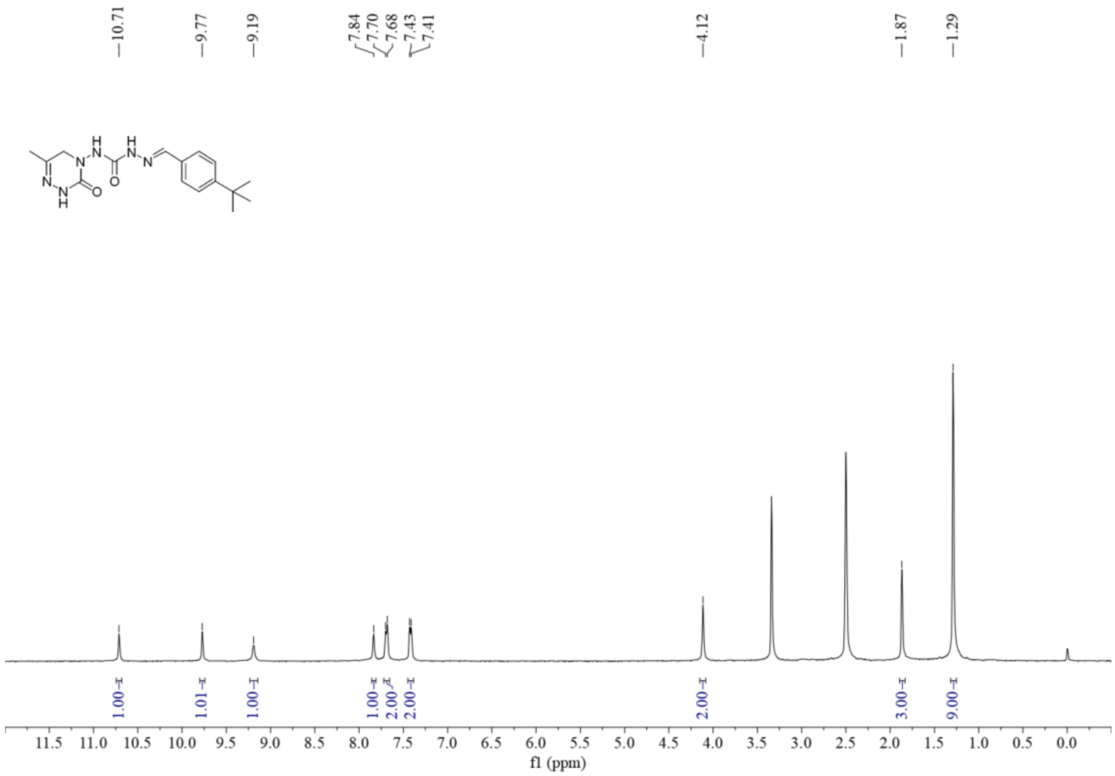

Fig. S17 ^{1}H NMR spectrum of **3i**

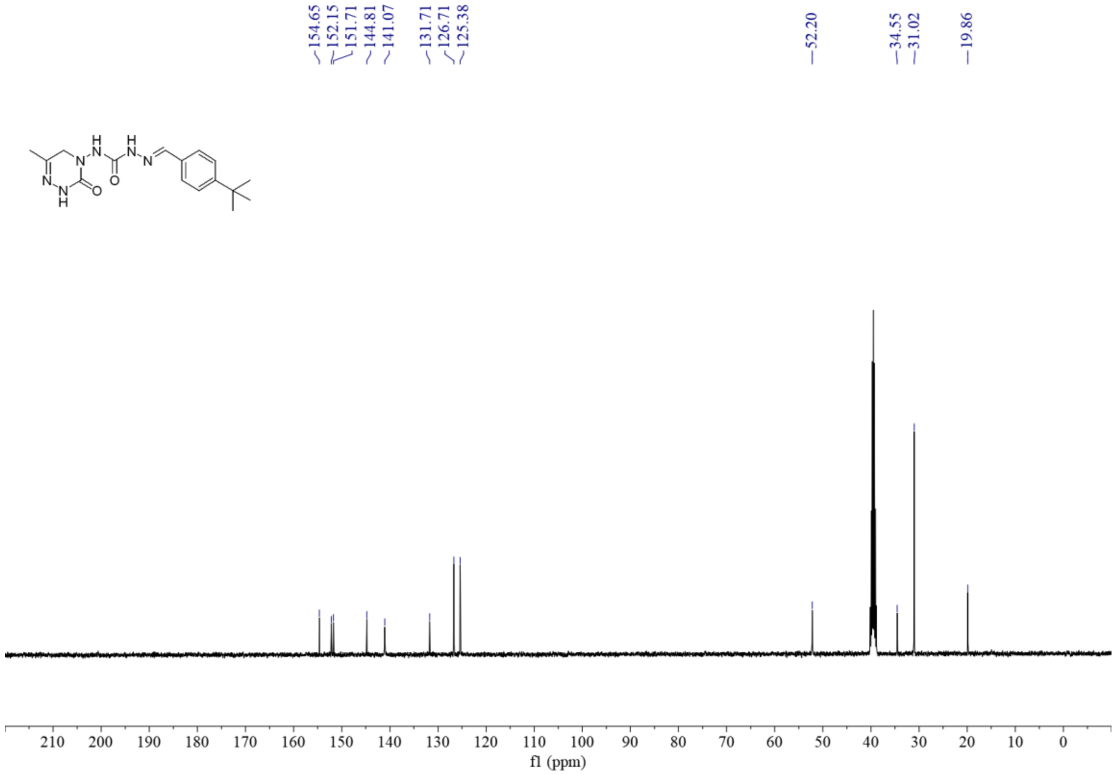

Fig. S18 ^{13}C NMR spectrum of **3i**

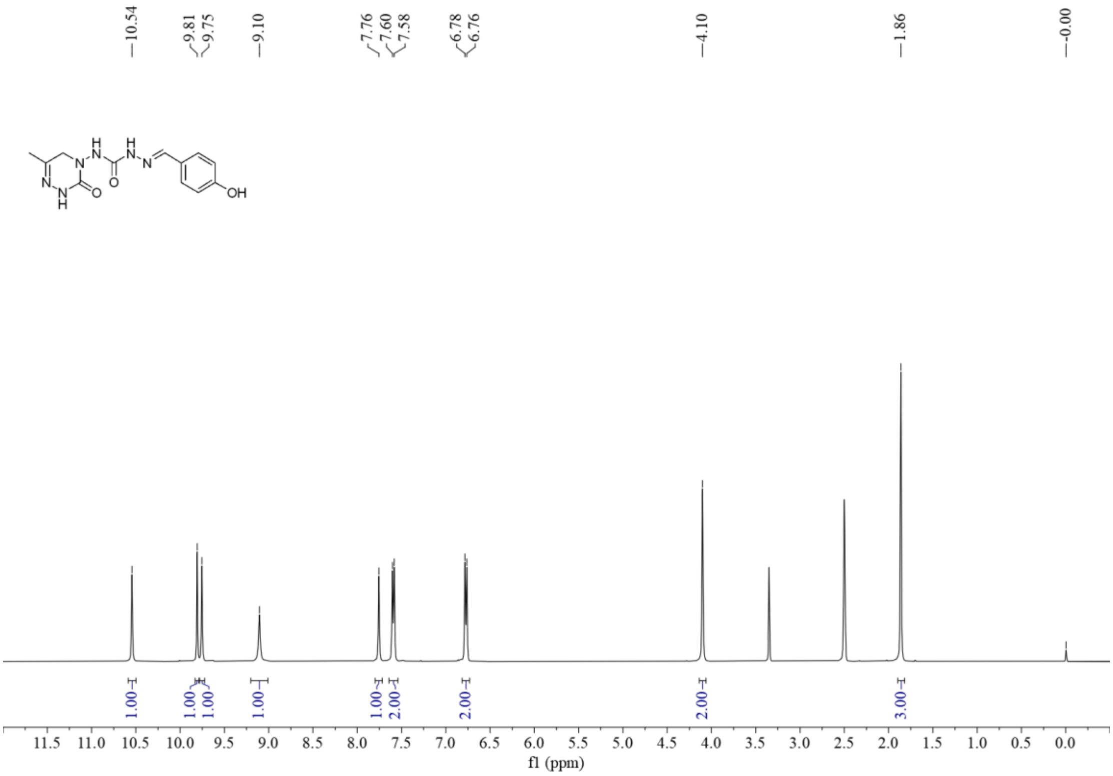

Fig. S19 ^{1}H NMR spectrum of **3j**

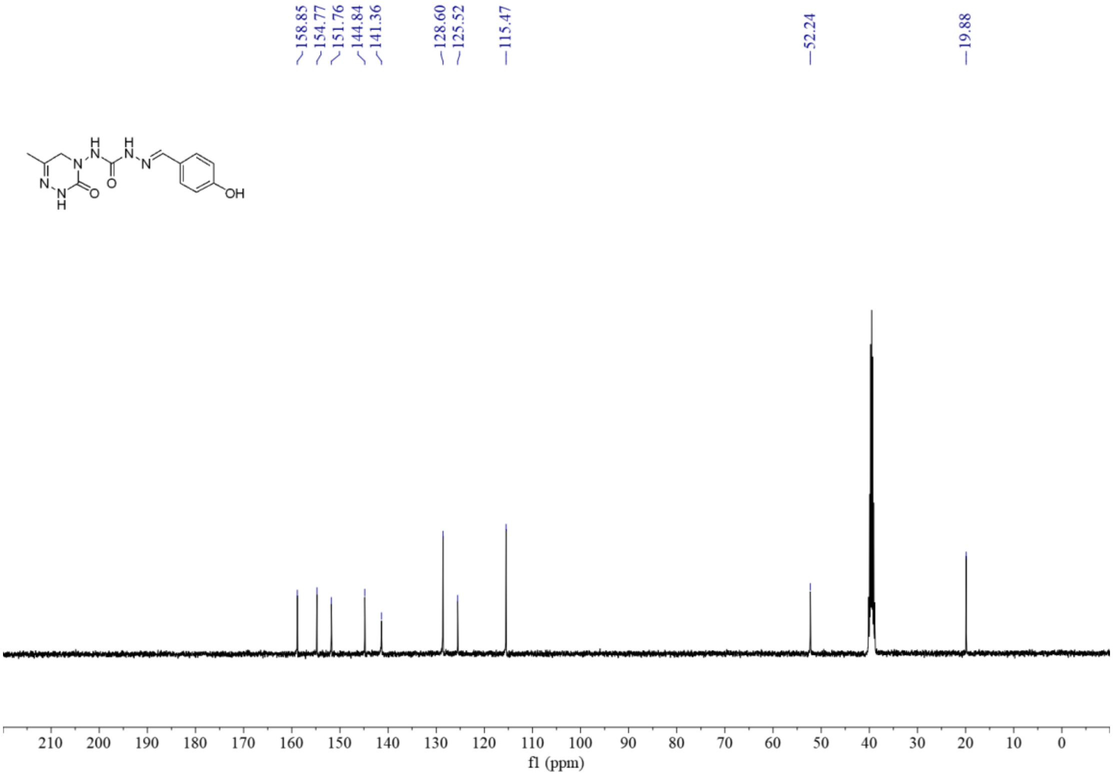

Fig. S20 ^{13}C NMR spectrum of **3j**

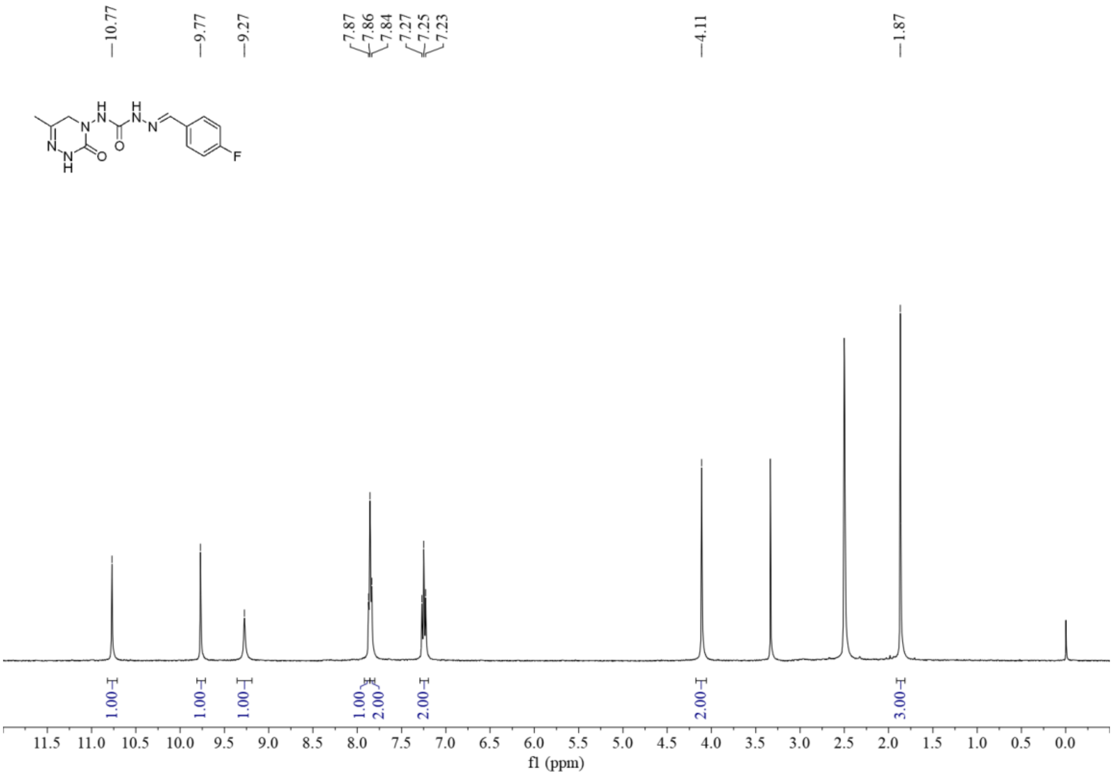

Fig. S21 ^{1}H NMR spectrum of **3k**

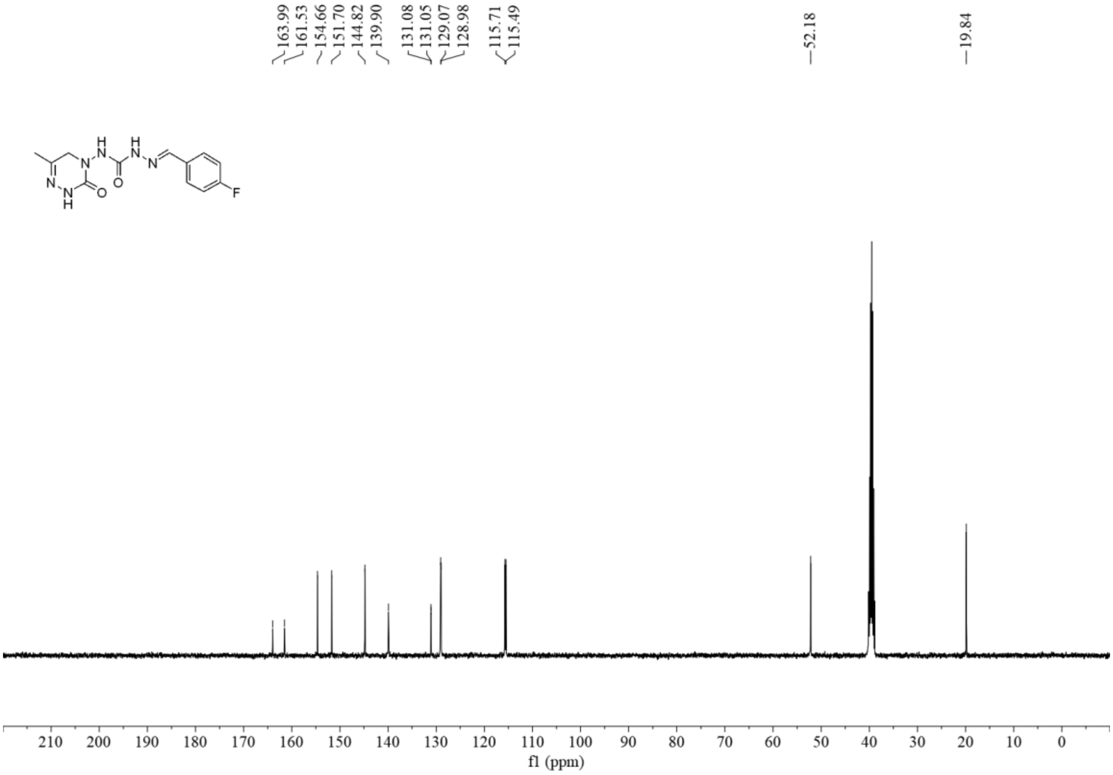

Fig. S22 ^{13}C NMR spectrum of **3k**

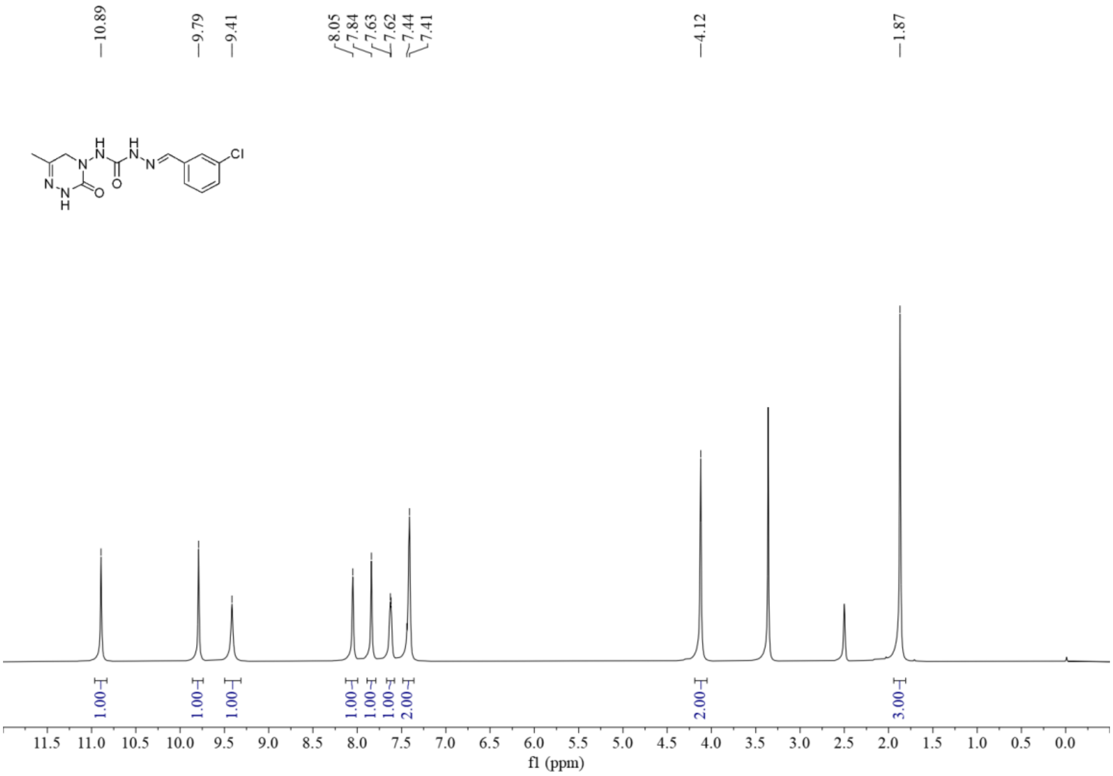

Fig. S23 ^{1}H NMR spectrum of **3l**

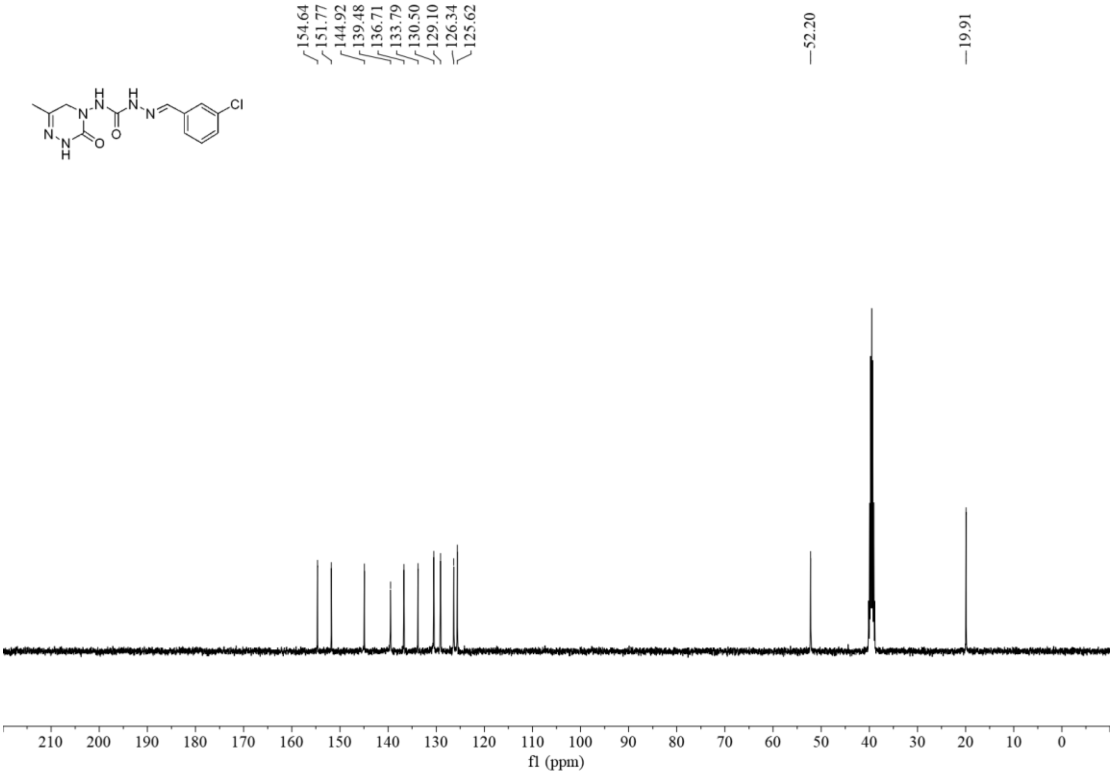

Fig. S24 ^{13}C NMR spectrum of **3l**

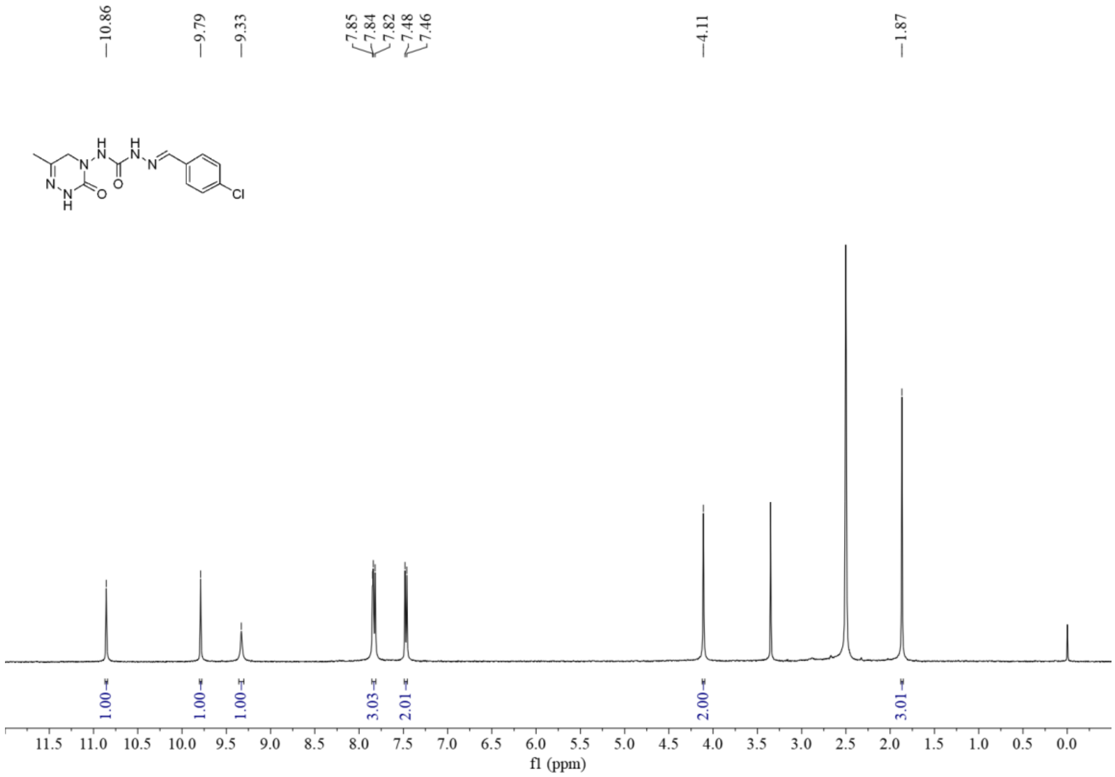

Fig. S25 ^{1}H NMR spectrum of **3m**

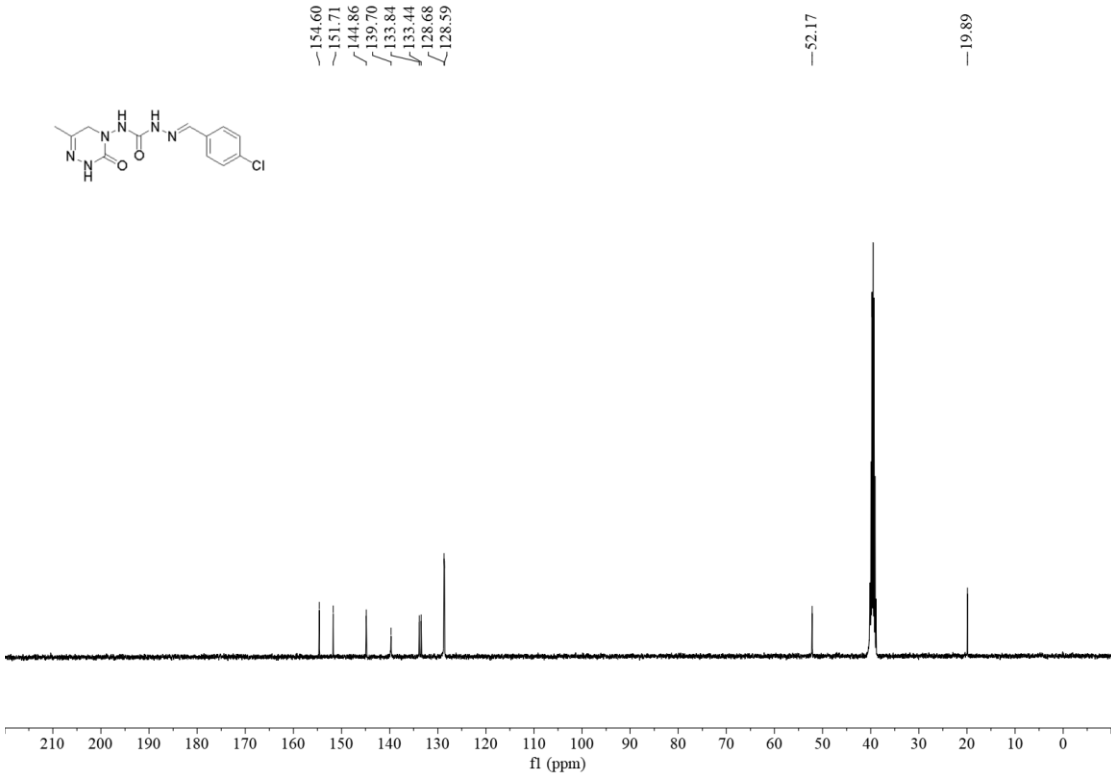

Fig. S26 ^{1}H NMR spectrum of **3m**

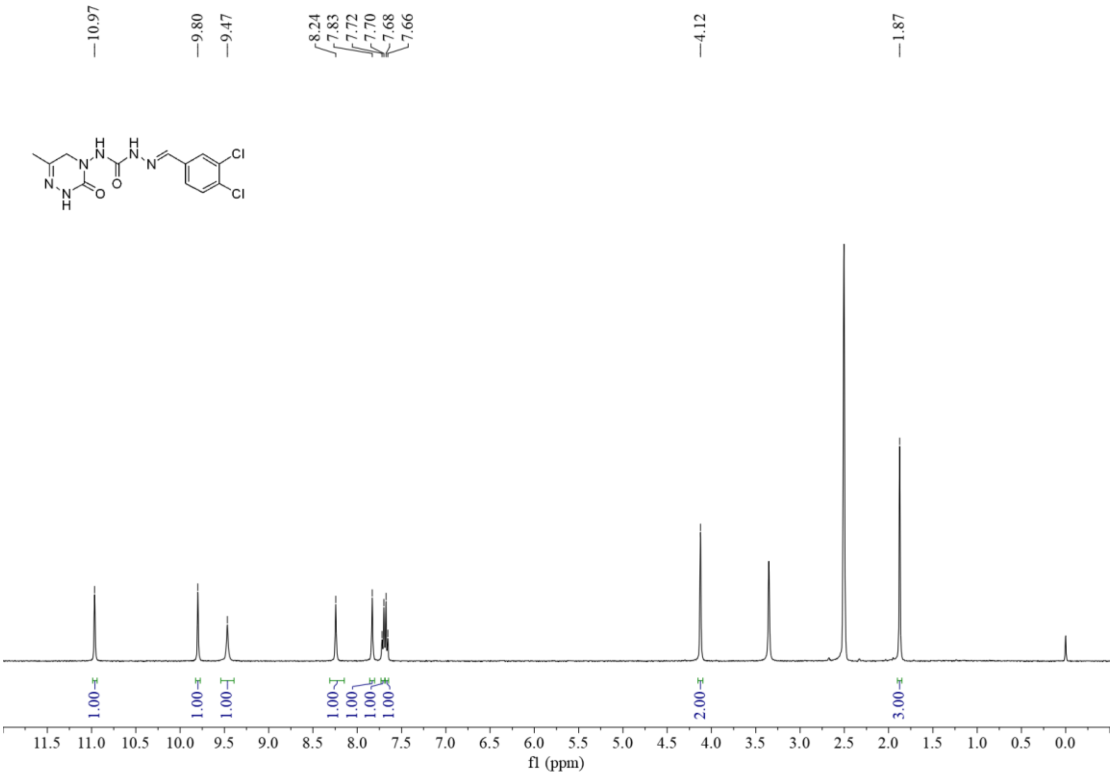

Fig. S27 ^{1}H NMR spectrum of **3n**

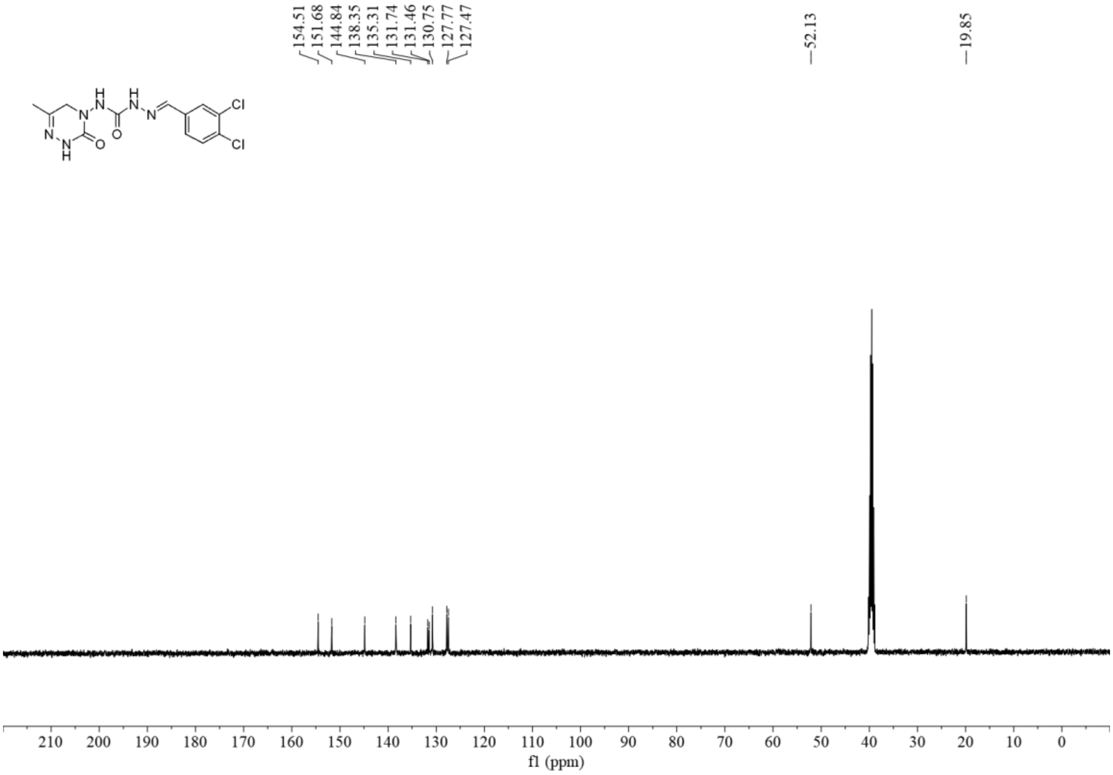

Fig. S28 ^{13}C NMR spectrum of **3n**

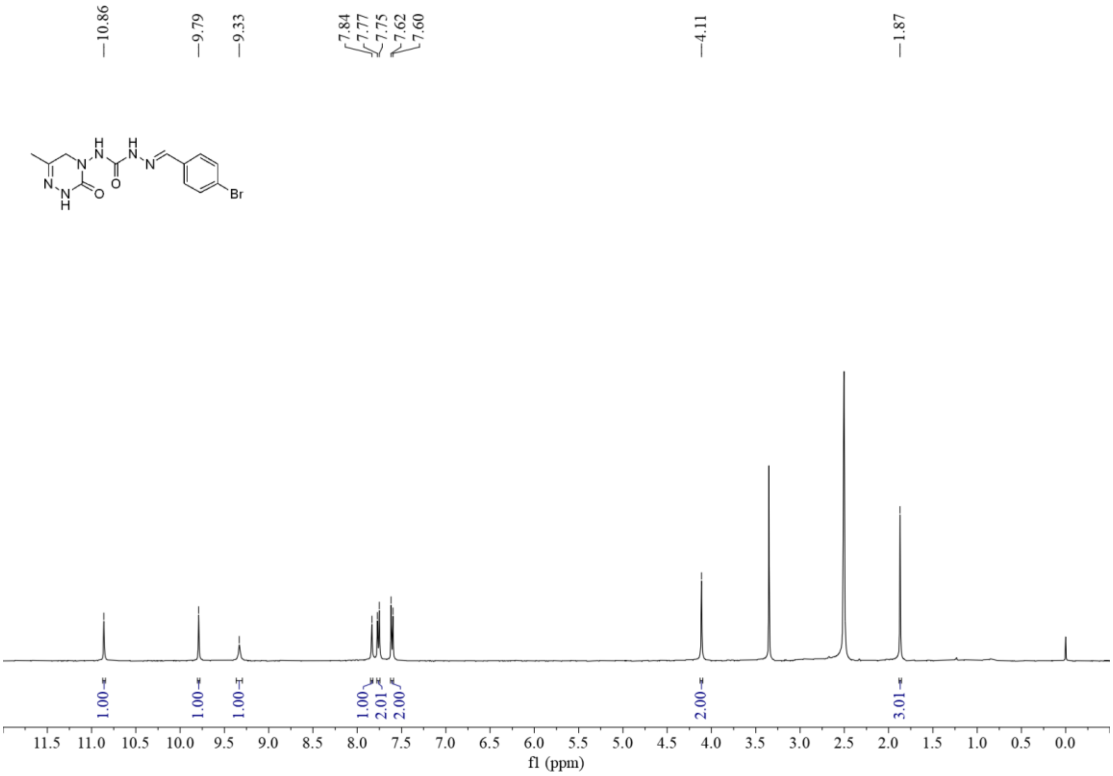

Fig. S29 ^{1}H NMR spectrum of **3o**

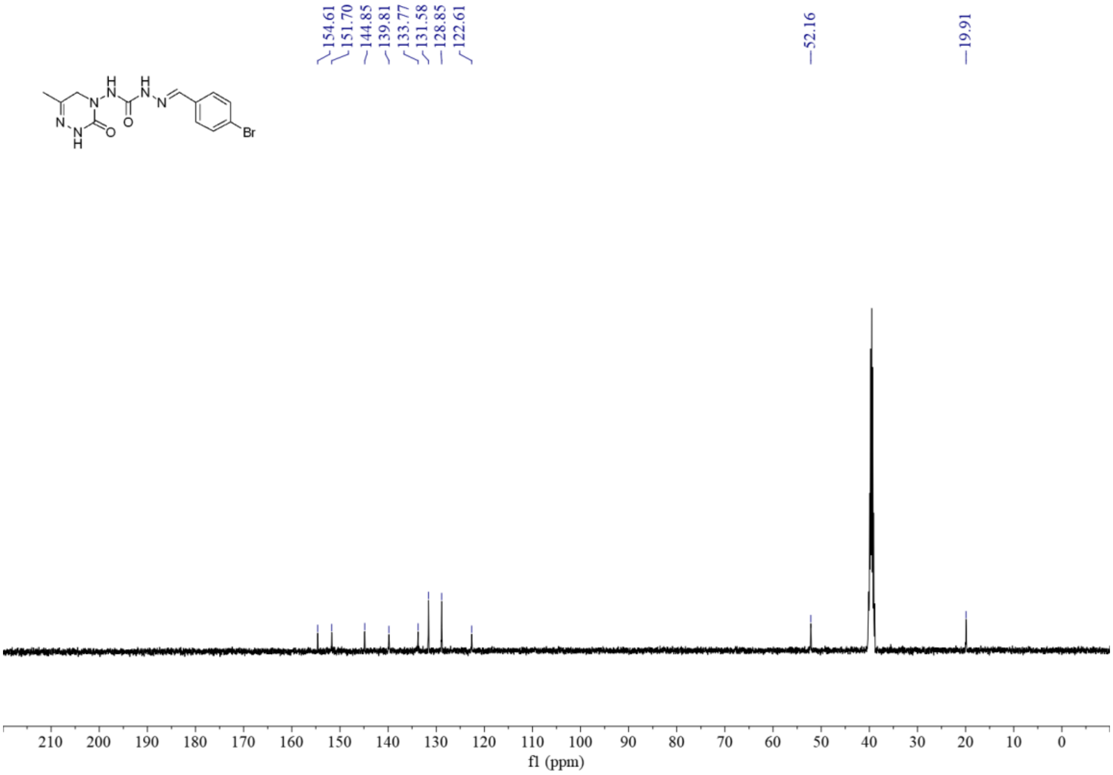

Fig. S30 ^{13}C NMR spectrum of **3o**

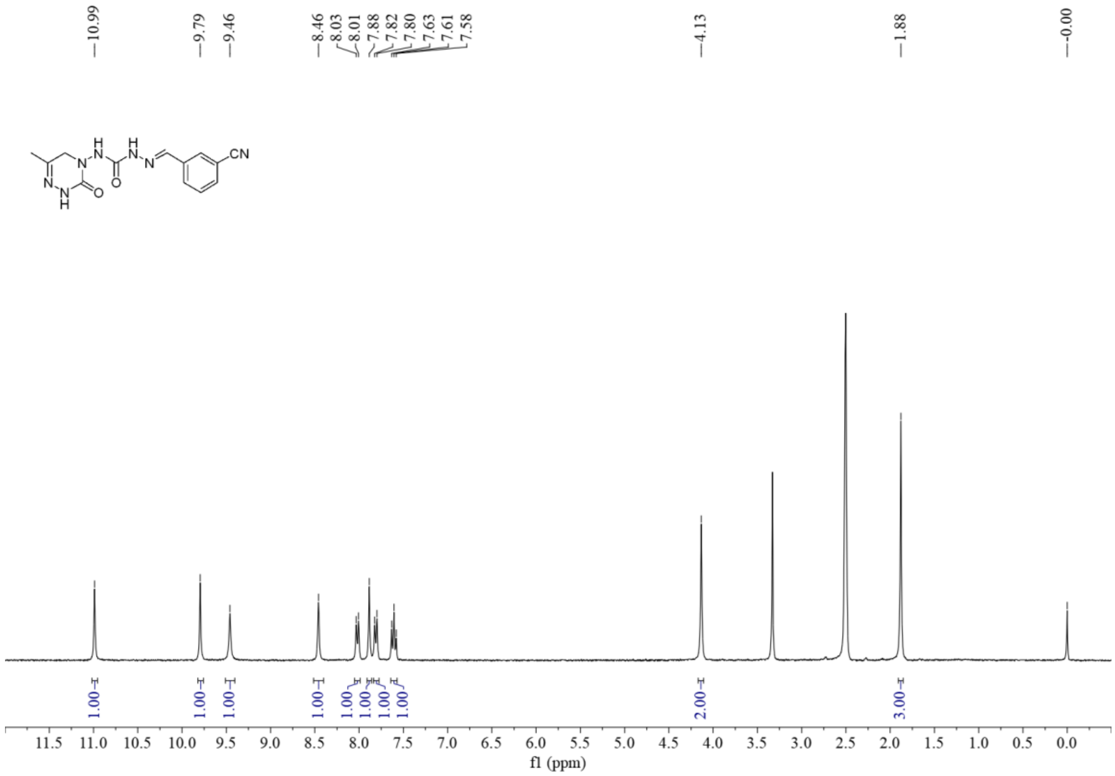

Fig. S31 ^{1}H NMR spectrum of **3p**

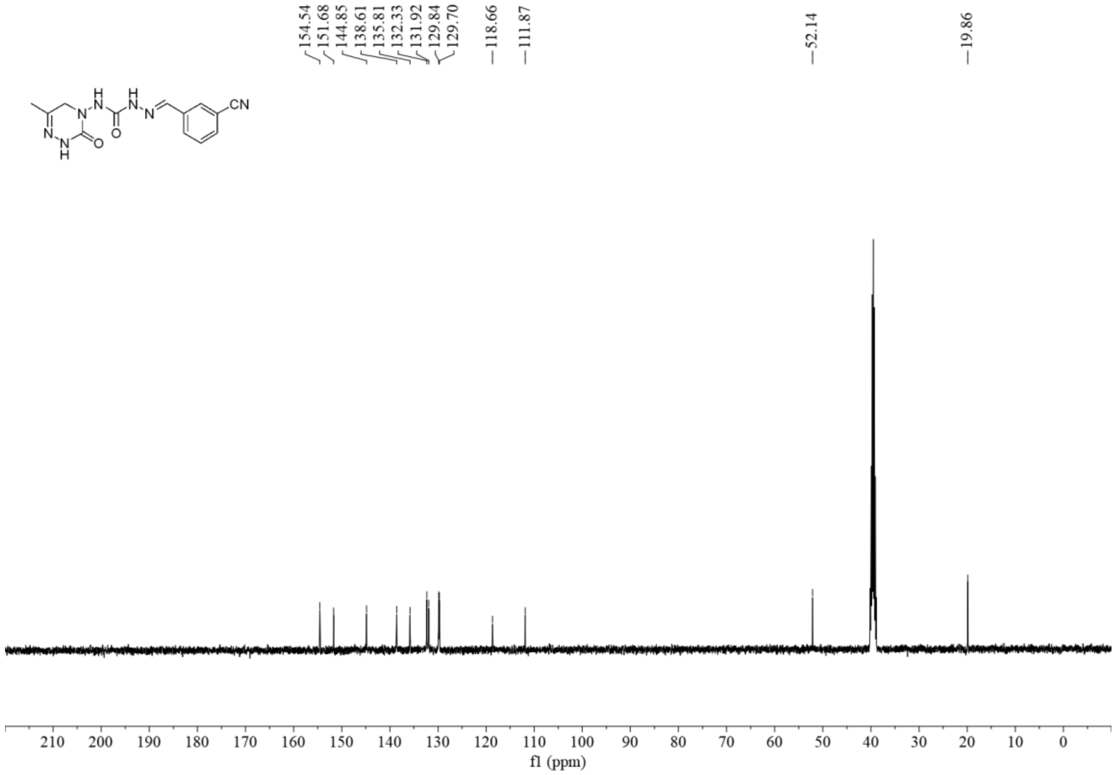

Fig. S32 ^{1}H NMR spectrum of **3p**

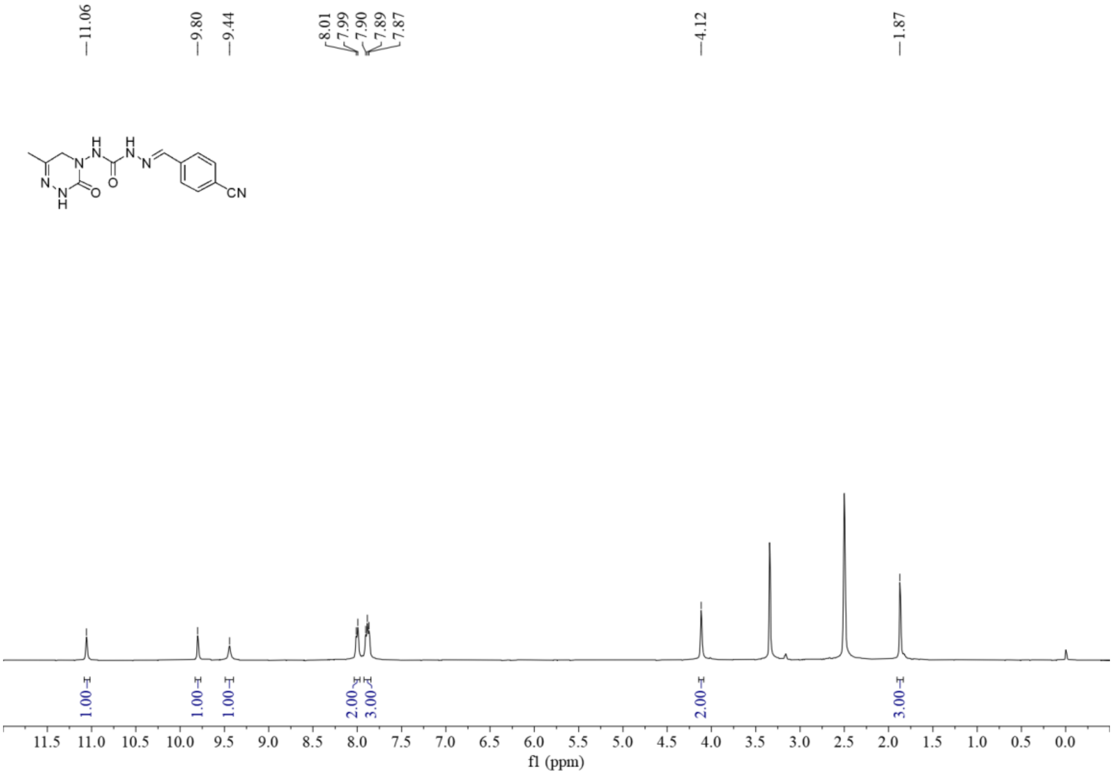

Fig. S33 ^{1}H NMR spectrum of **3q**

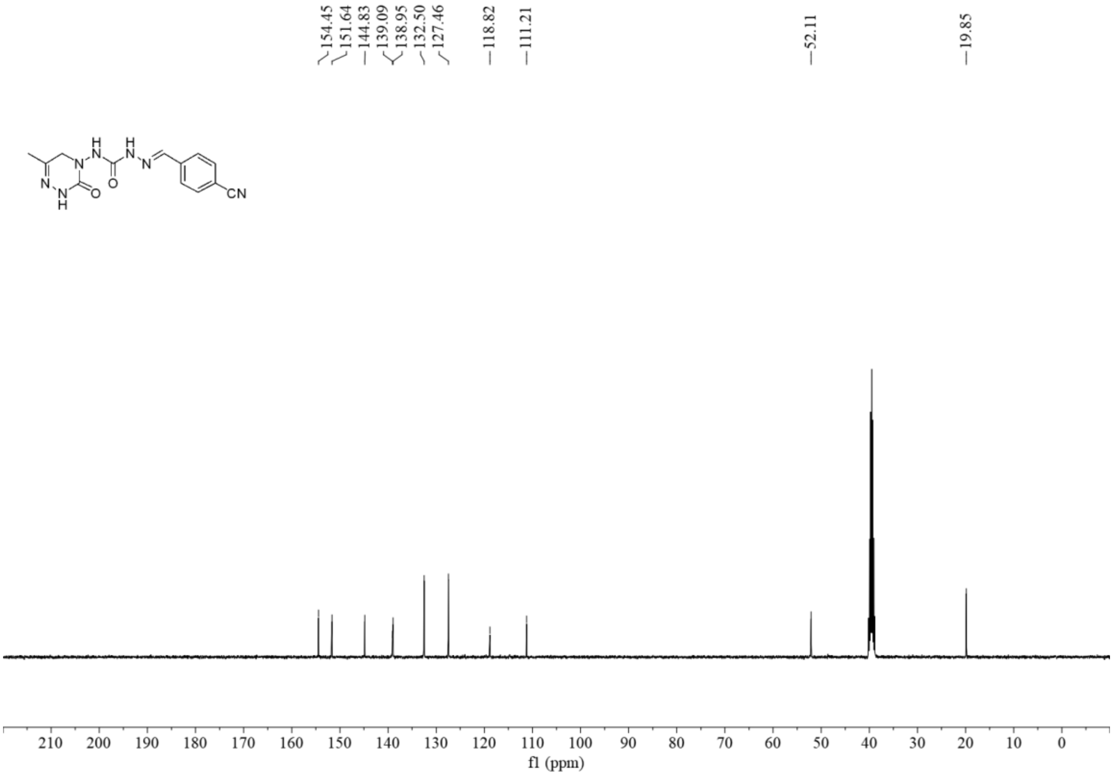

Fig. S34 ^{1}H NMR spectrum of **3q**

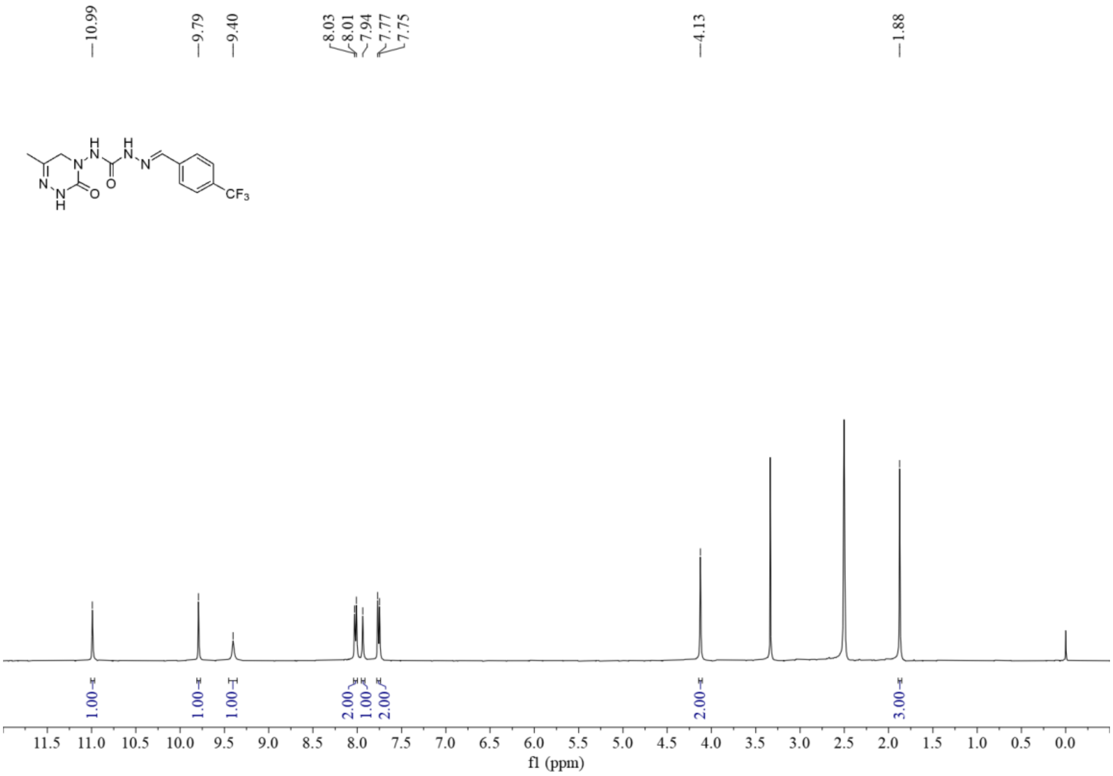

Fig. S35 ^{1}H NMR spectrum of **3r**

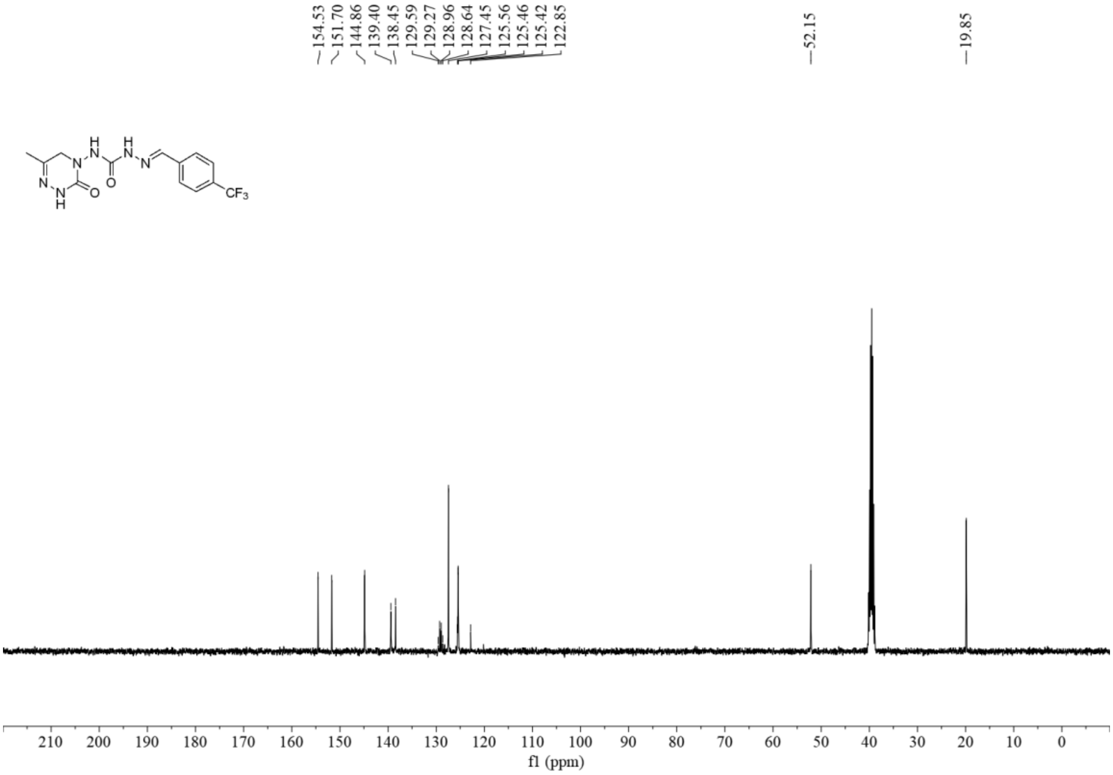

Fig. S36 ^{1}H NMR spectrum of **3r**

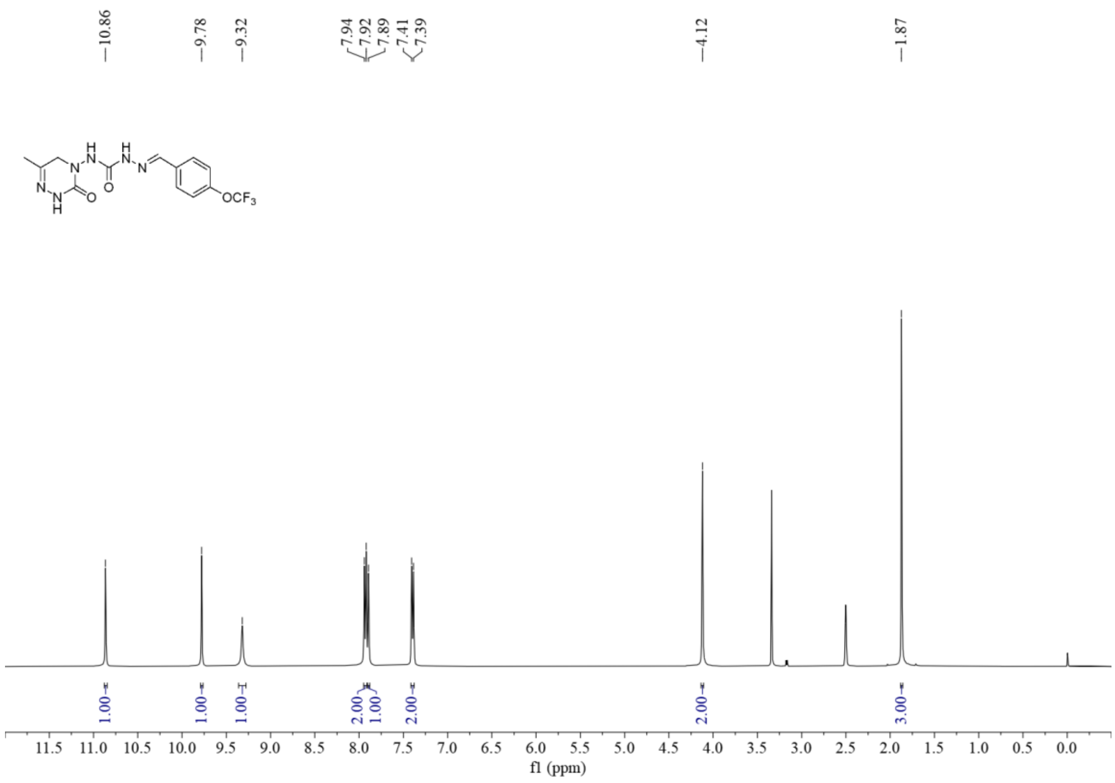

Fig. S37 ^{1}H NMR spectrum of **3s**

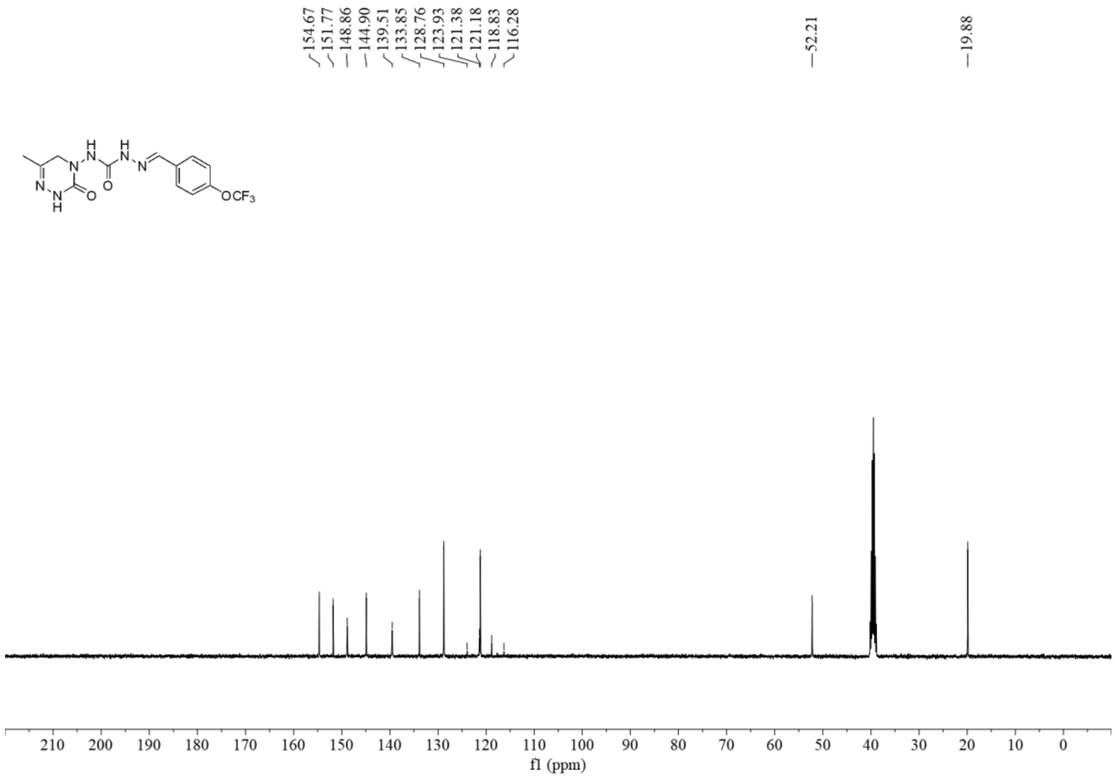

Fig. S38 ^{13}C NMR spectrum of **3s**

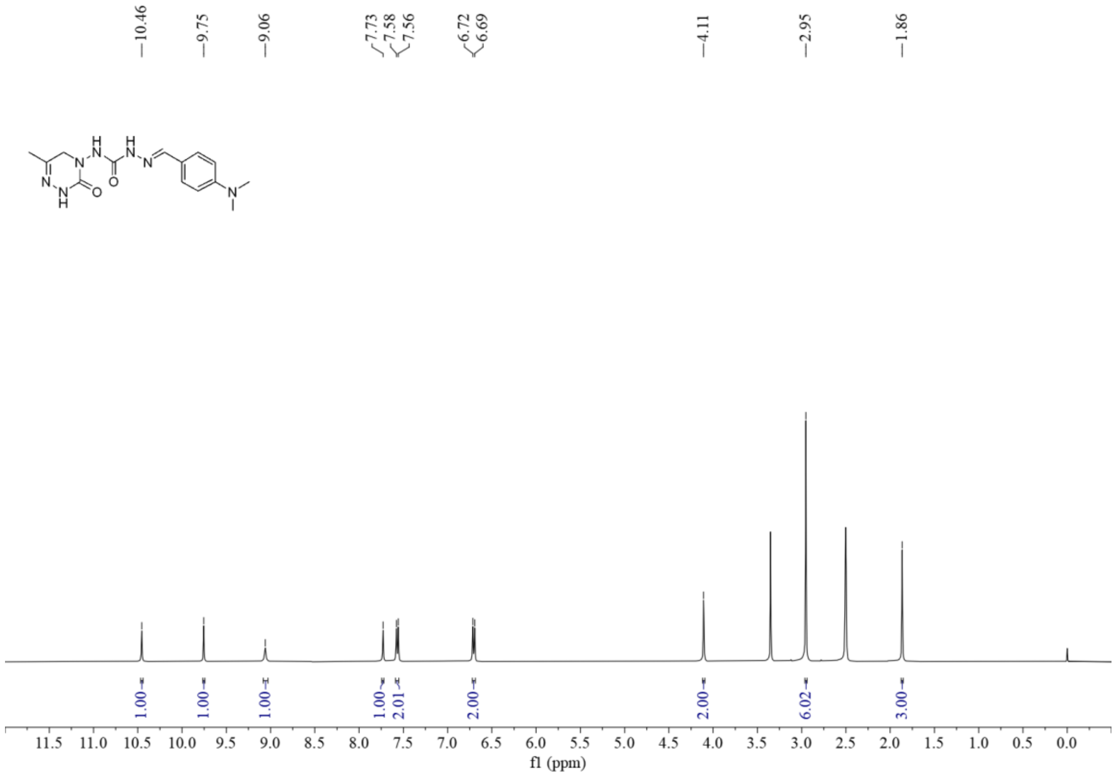

Fig. S39 ^{1}H NMR spectrum of **3t**

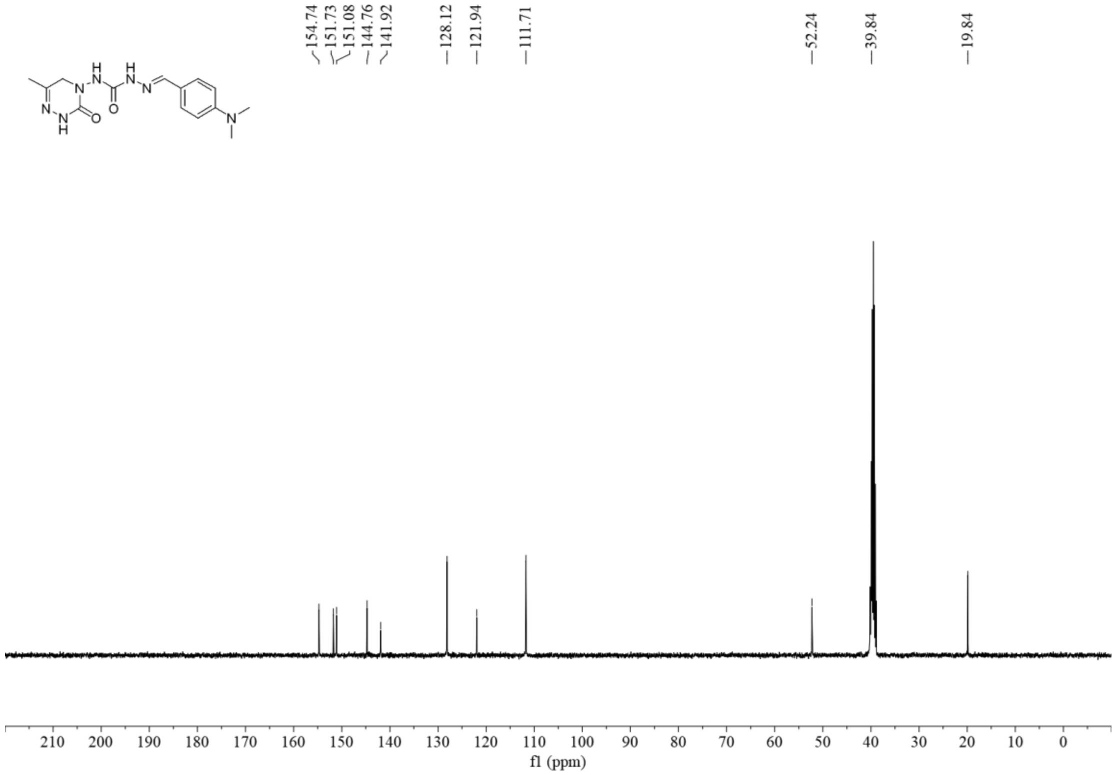

Fig. S40 ^{13}C NMR spectrum of **3t**

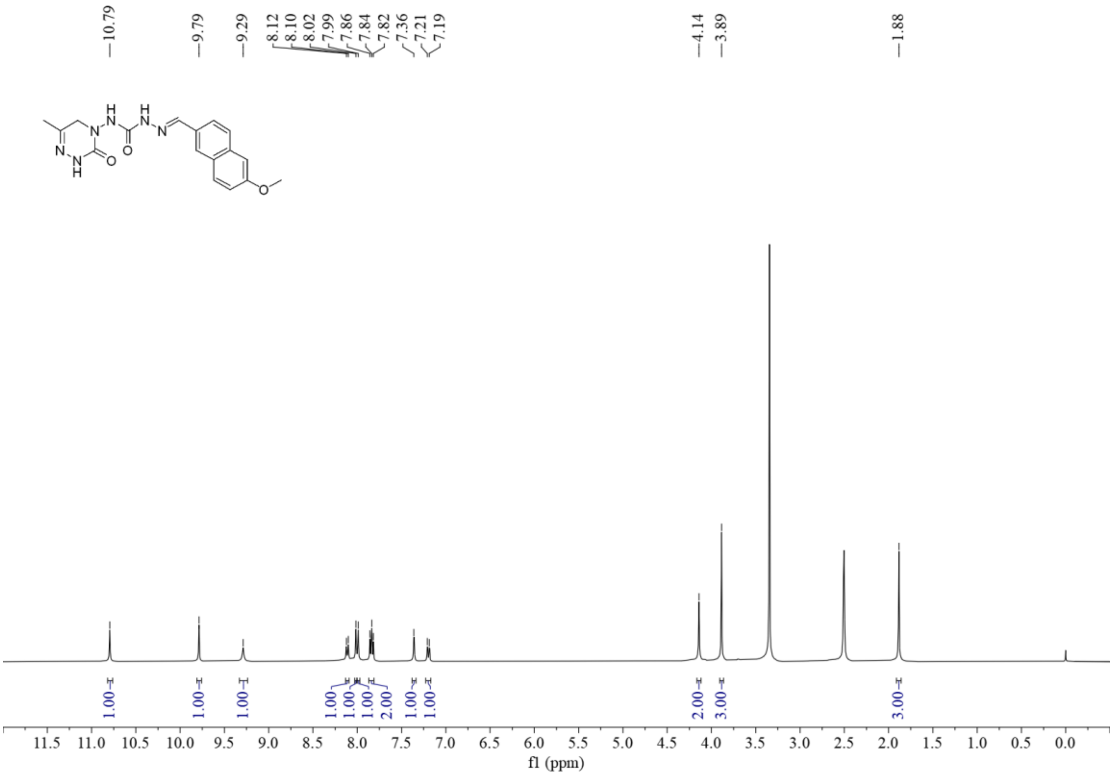

Fig. S41 ^{1}H NMR spectrum of **3u**

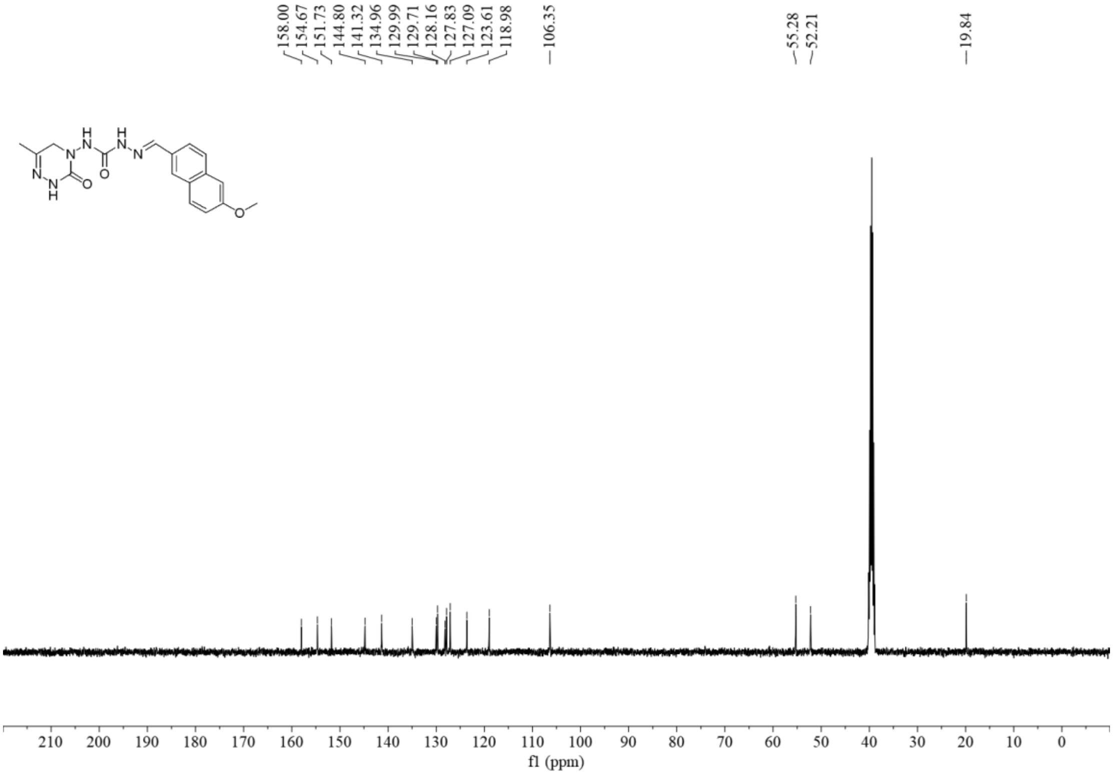

Fig. S42 ^{13}C NMR spectrum of **3u**

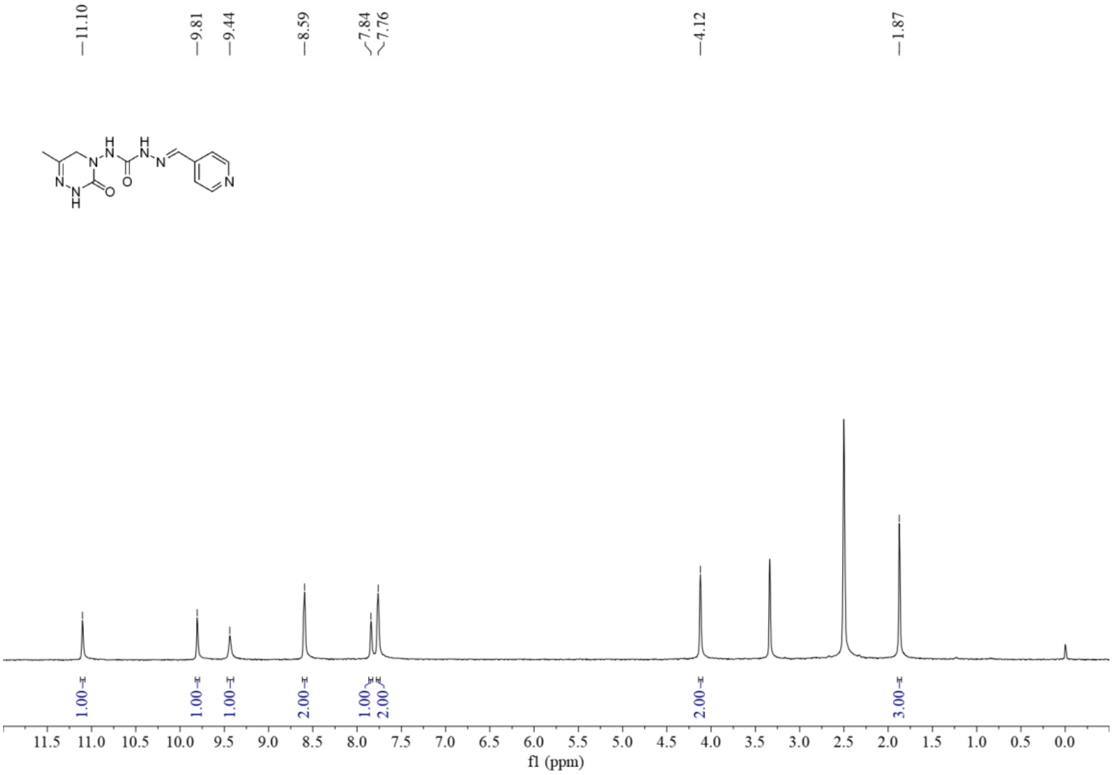

Fig. S43 ^{1}H NMR spectrum of **3v**

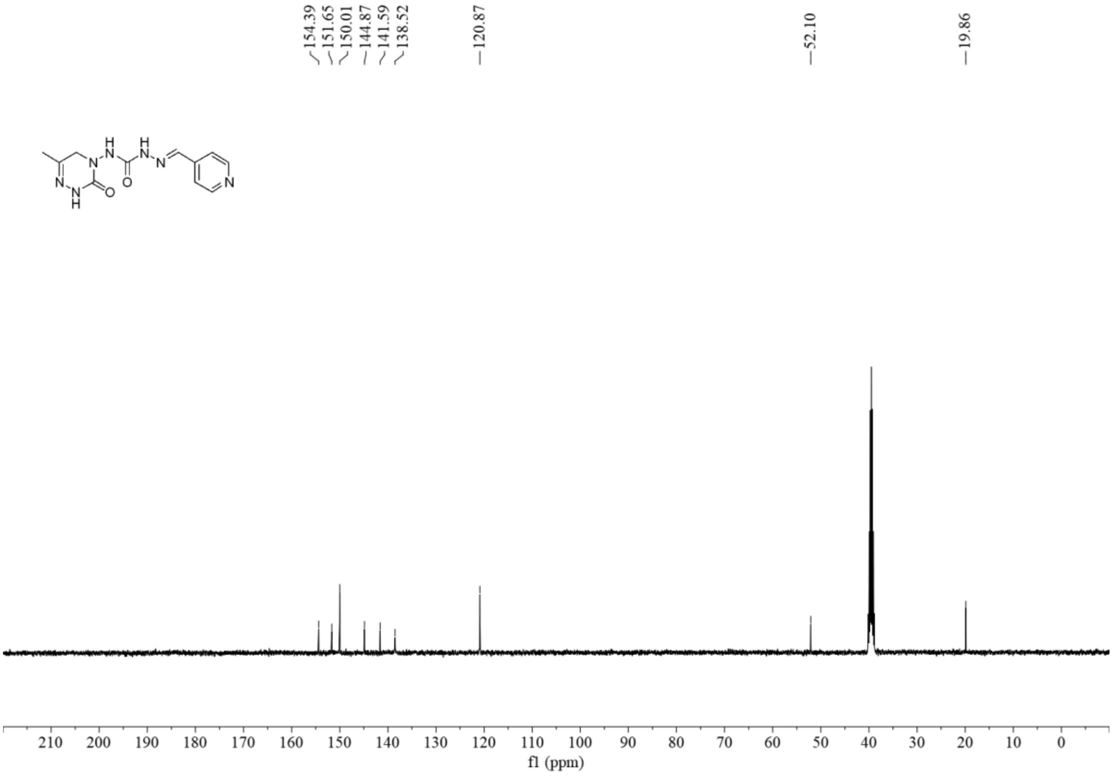

Fig. S44 ^{13}C NMR spectrum of **3v**

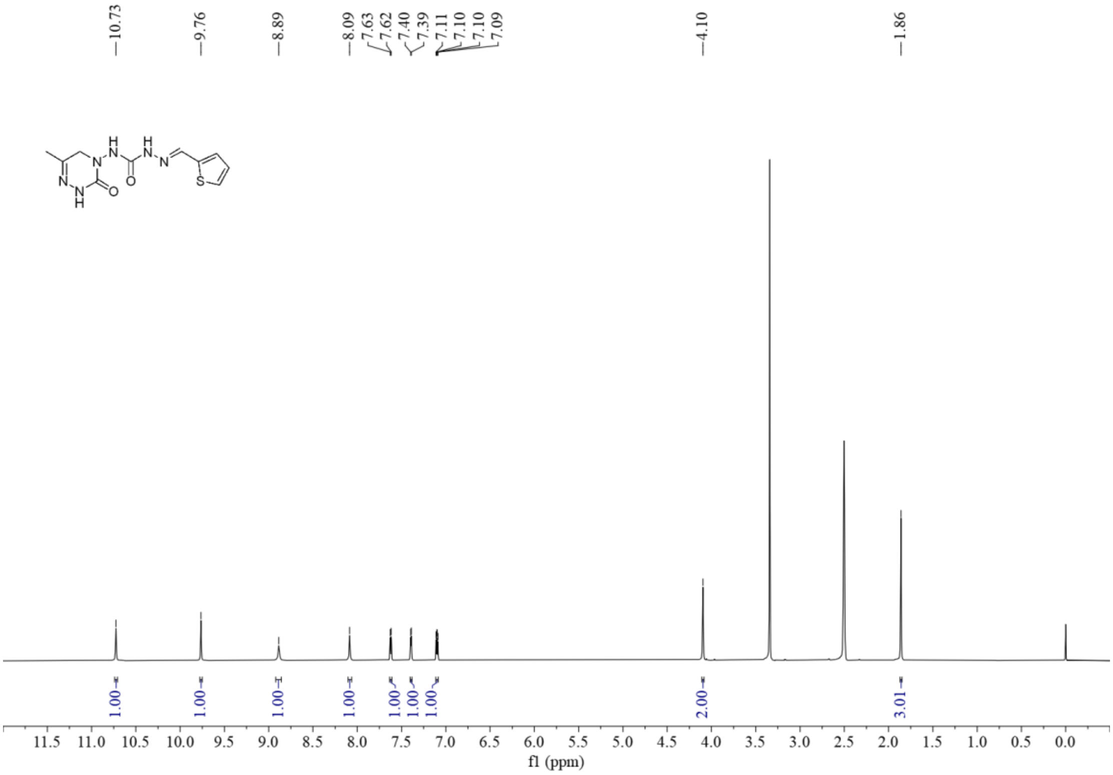

Fig. S45 ^{1}H NMR spectrum of **3w**

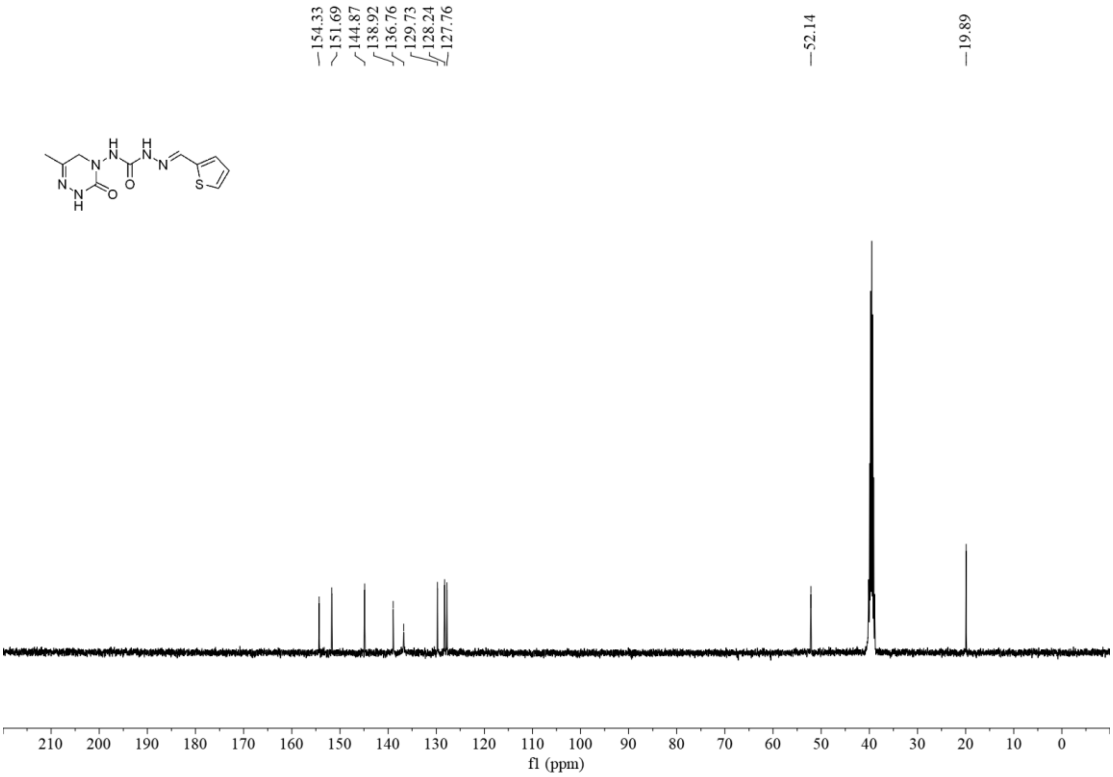

Fig. S46 ^{13}C NMR spectrum of **3w**

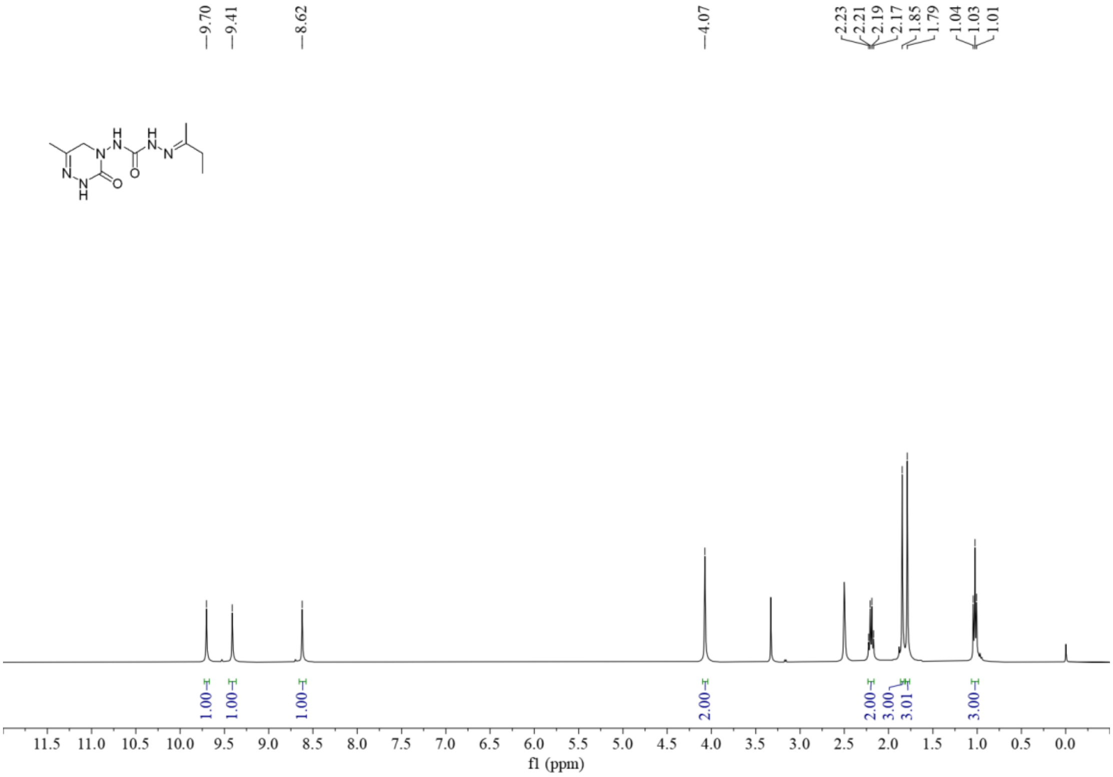

Fig. S47 ^{1}H NMR spectrum of **5a**

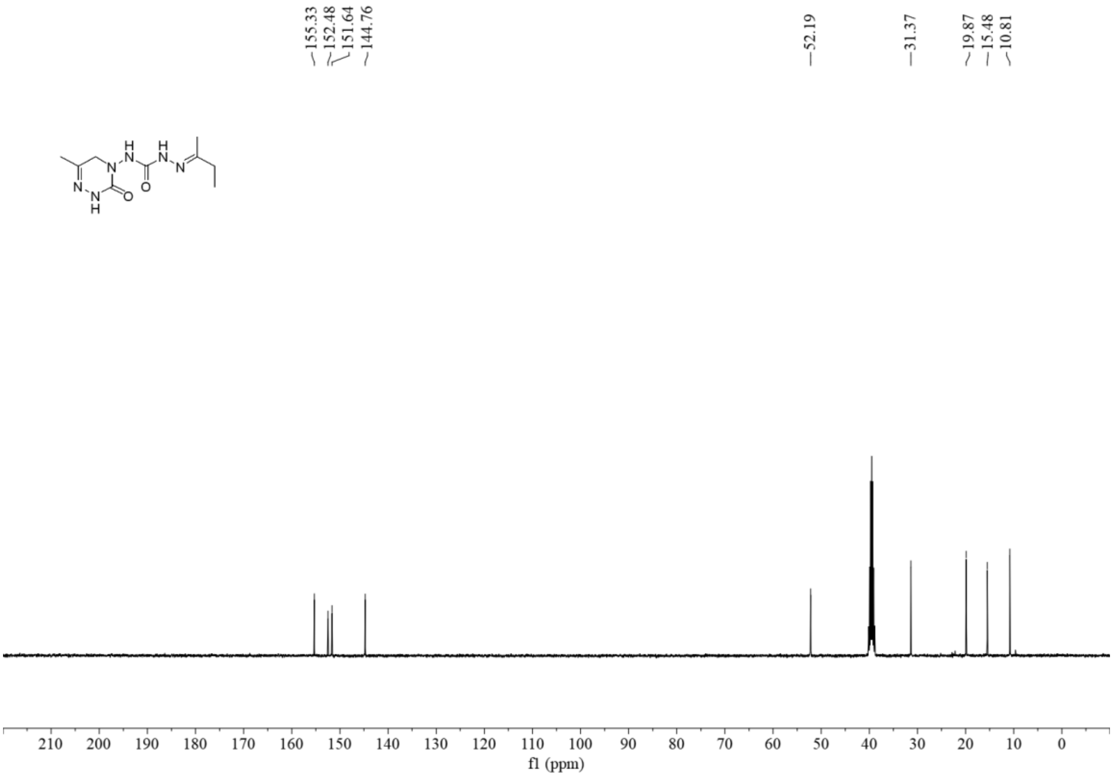

Fig. S48 ^{13}C NMR spectrum of **5a**

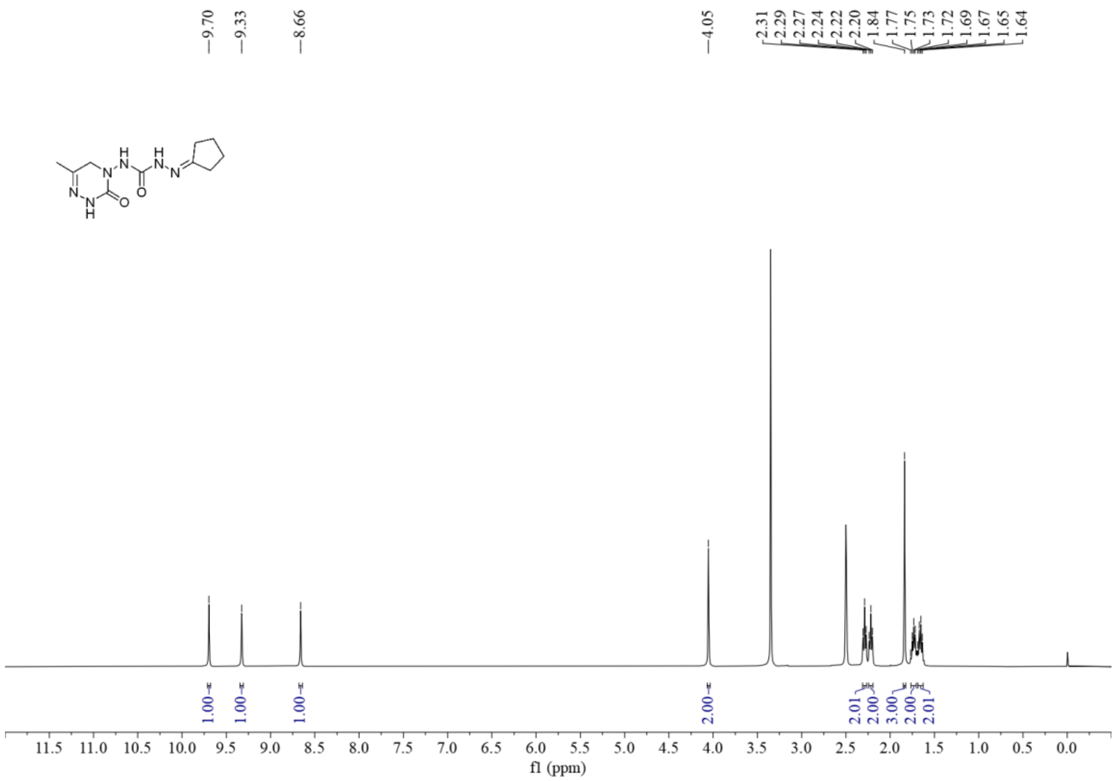

Fig. S49 ^{1}H NMR spectrum of **5b**

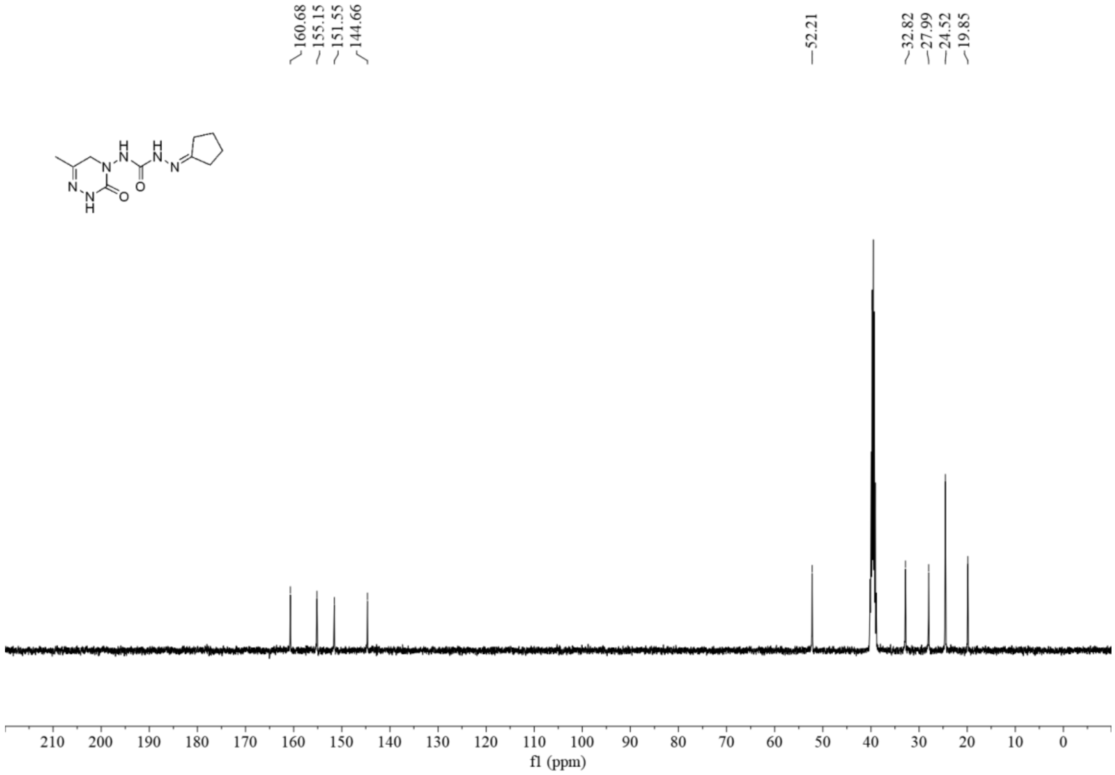

Fig. S50 ^{13}C NMR spectrum of **5b**

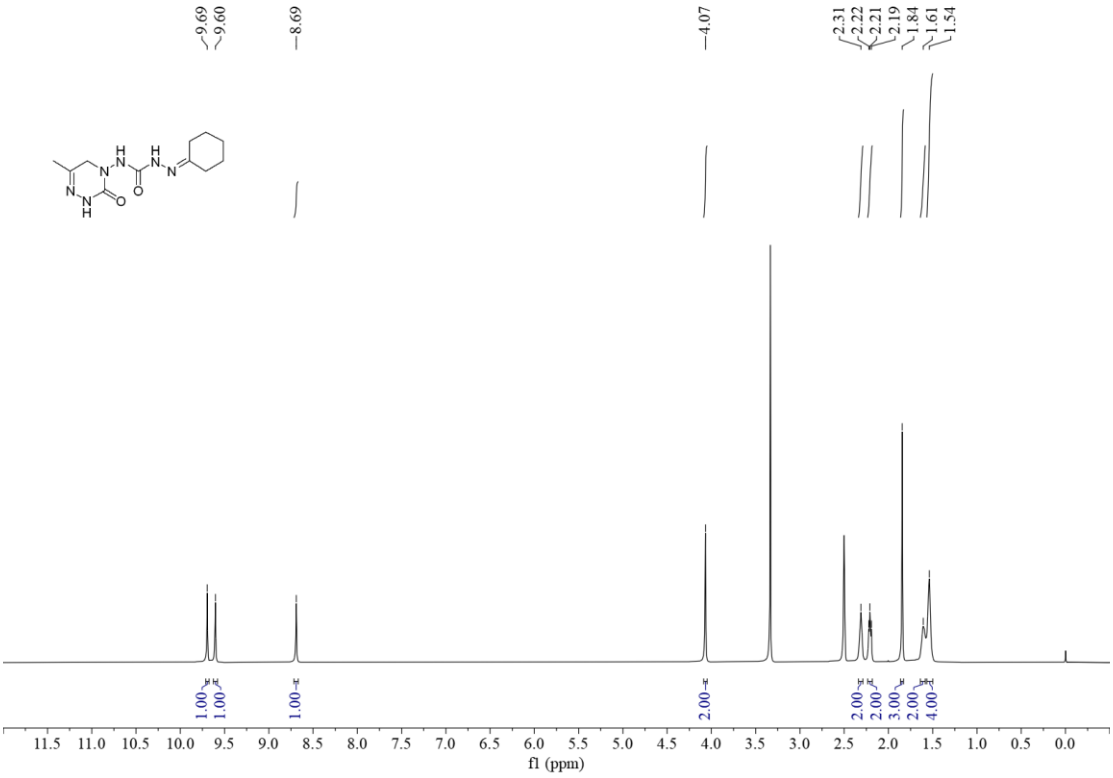

Fig. S51 ^{1}H NMR spectrum of **5c**

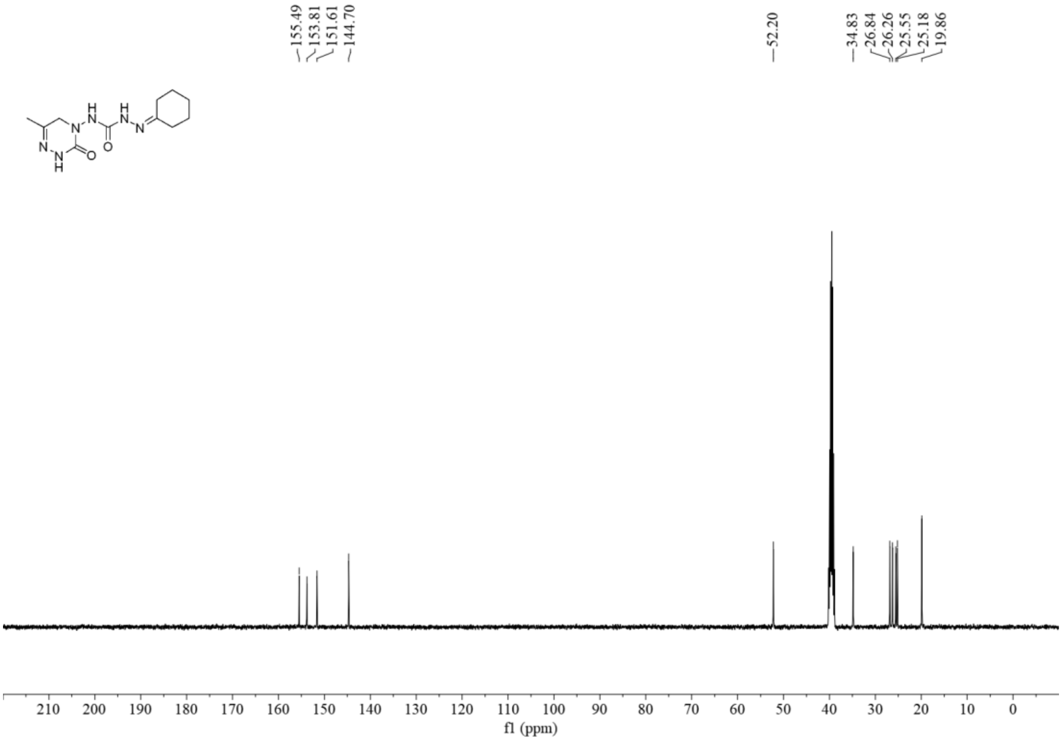

Fig. S52 ^{13}C NMR spectrum of **5c**

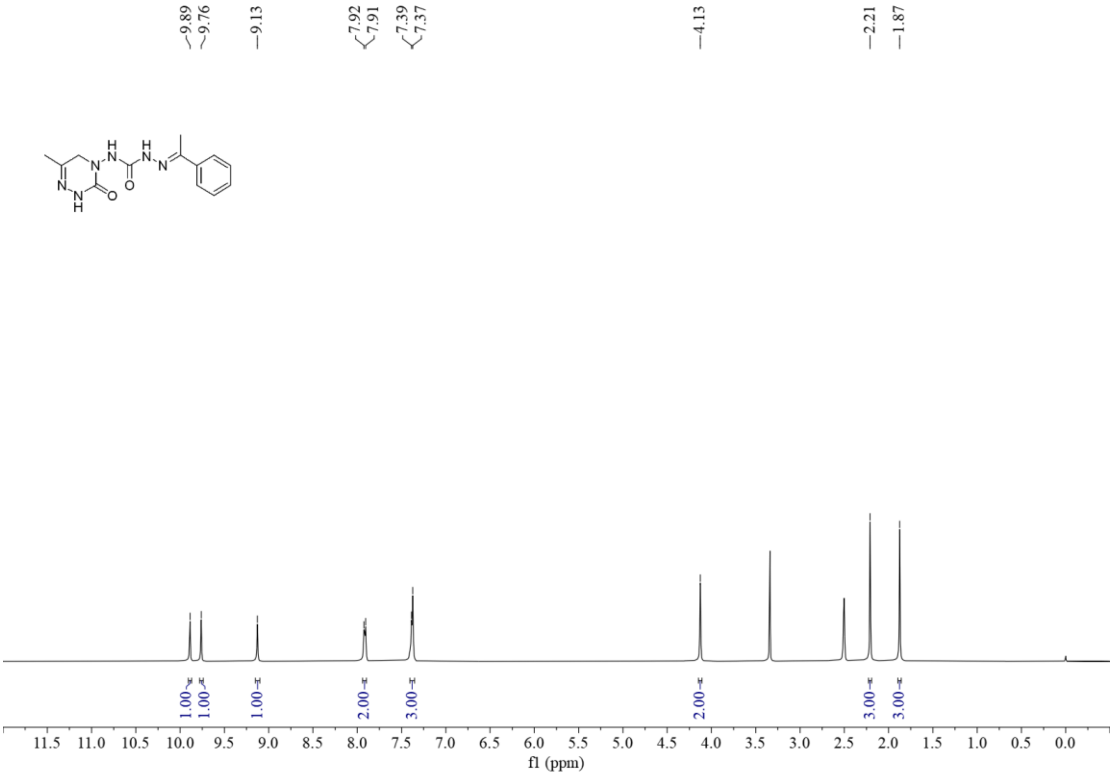

Fig. S53 ^{1}H NMR spectrum of **5d**

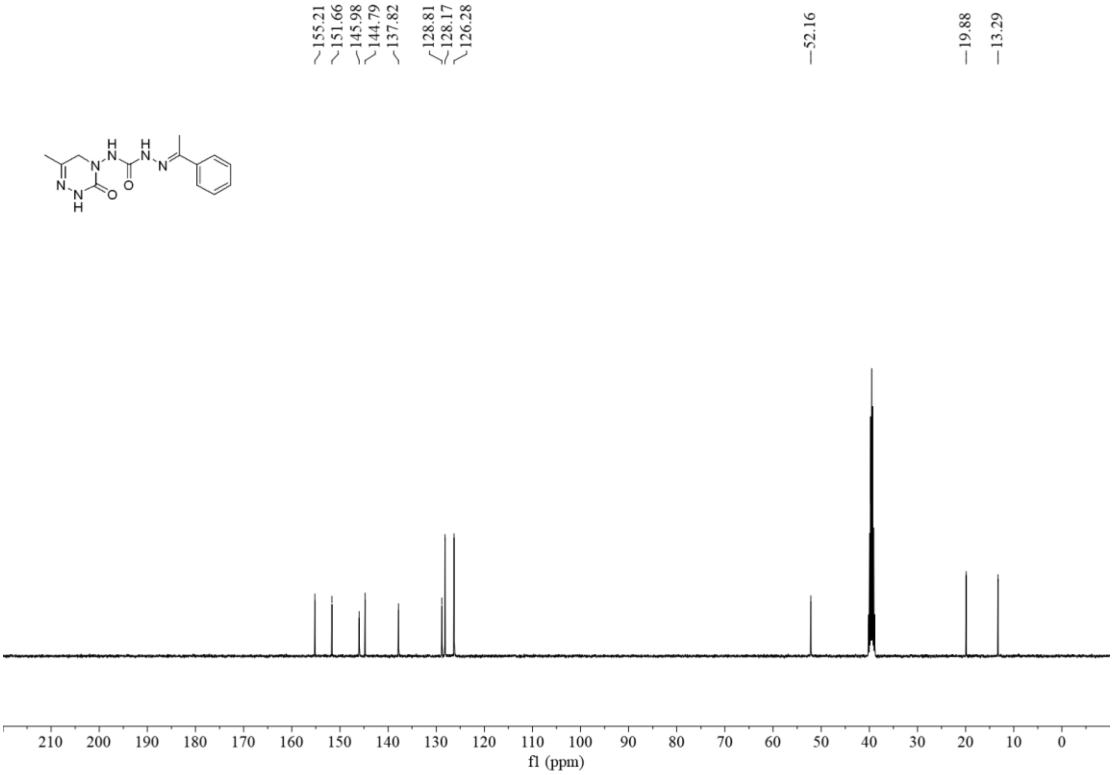

Fig. S54 ^{13}C NMR spectrum of **5d**

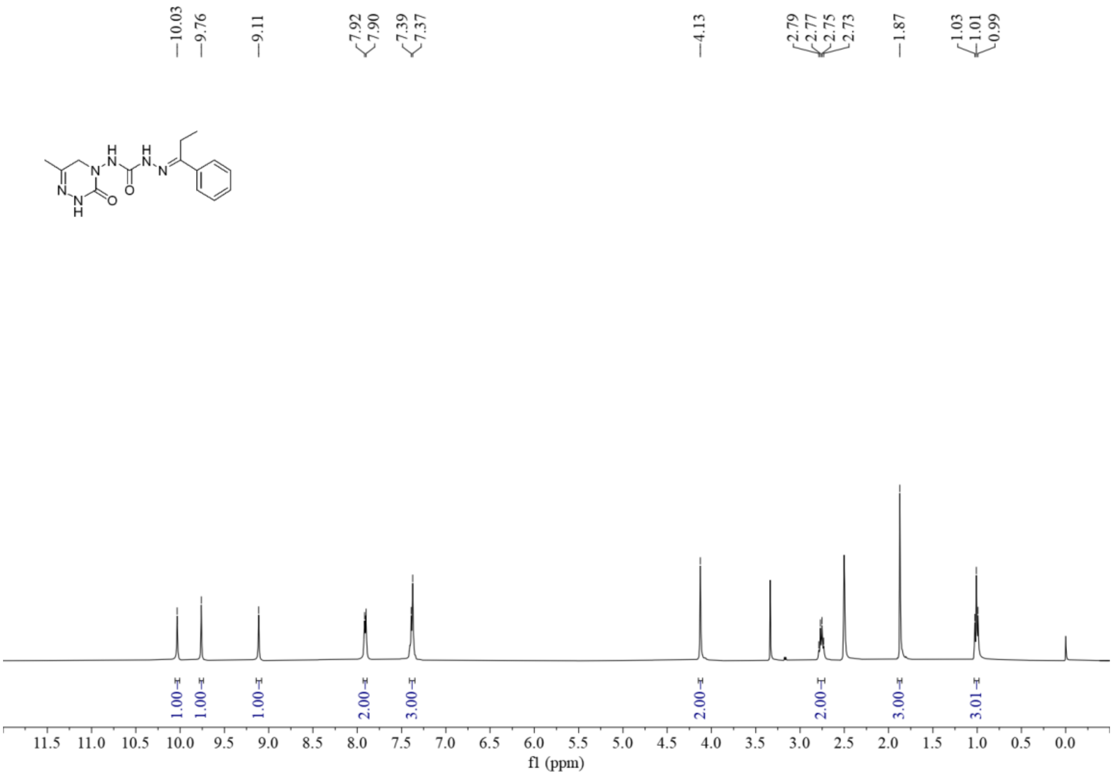

Fig. S55 ^{1}H NMR spectrum of **5e**

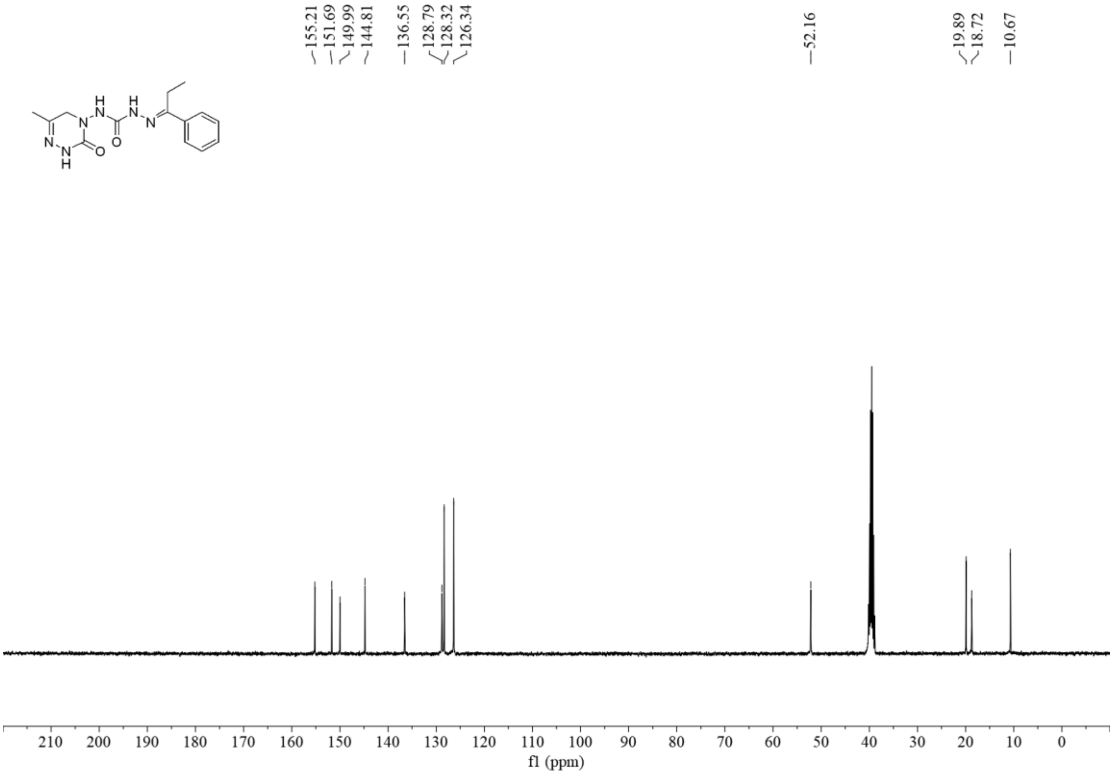

Fig. S56 ^{13}C NMR spectrum of **5e**

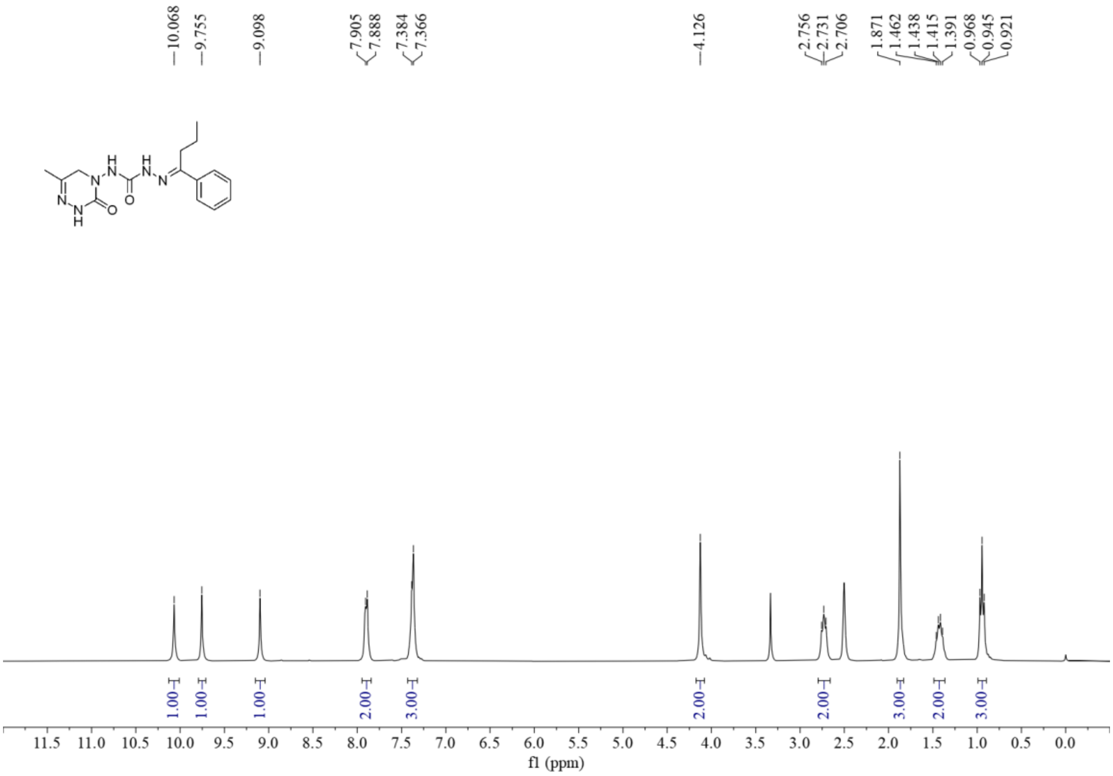

Fig. S57 ^{1}H NMR spectrum of **5f**

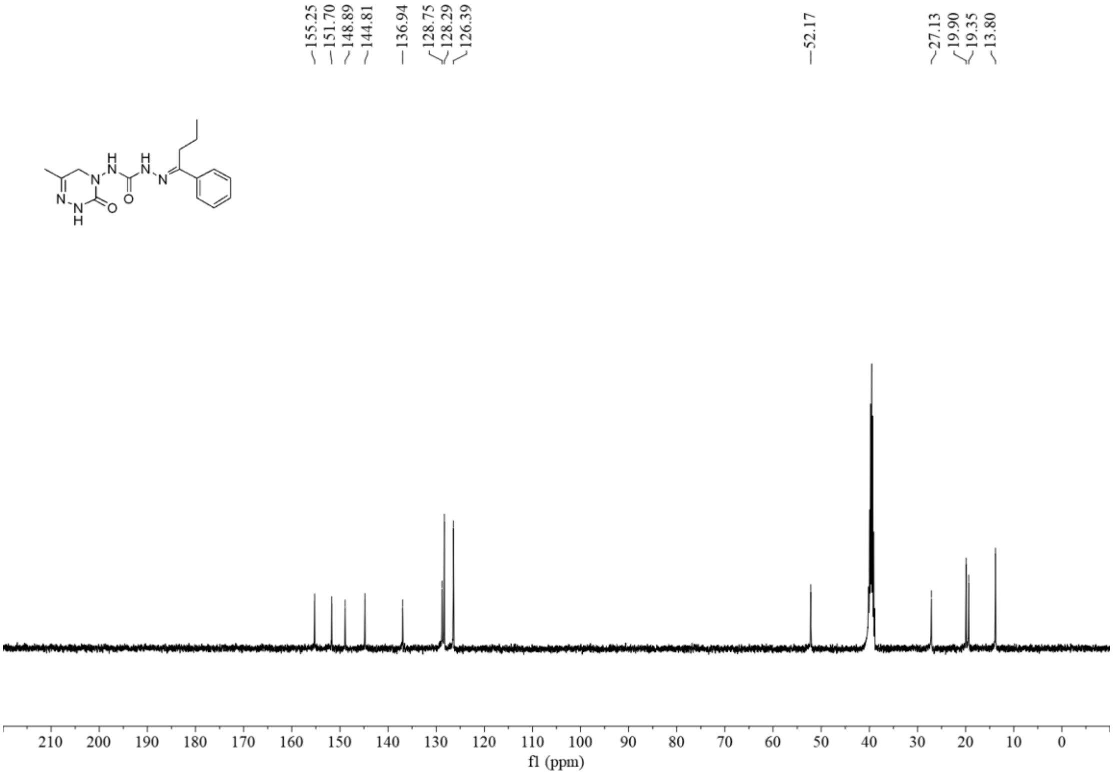

Fig. S58 ^{13}C NMR spectrum of **5f**

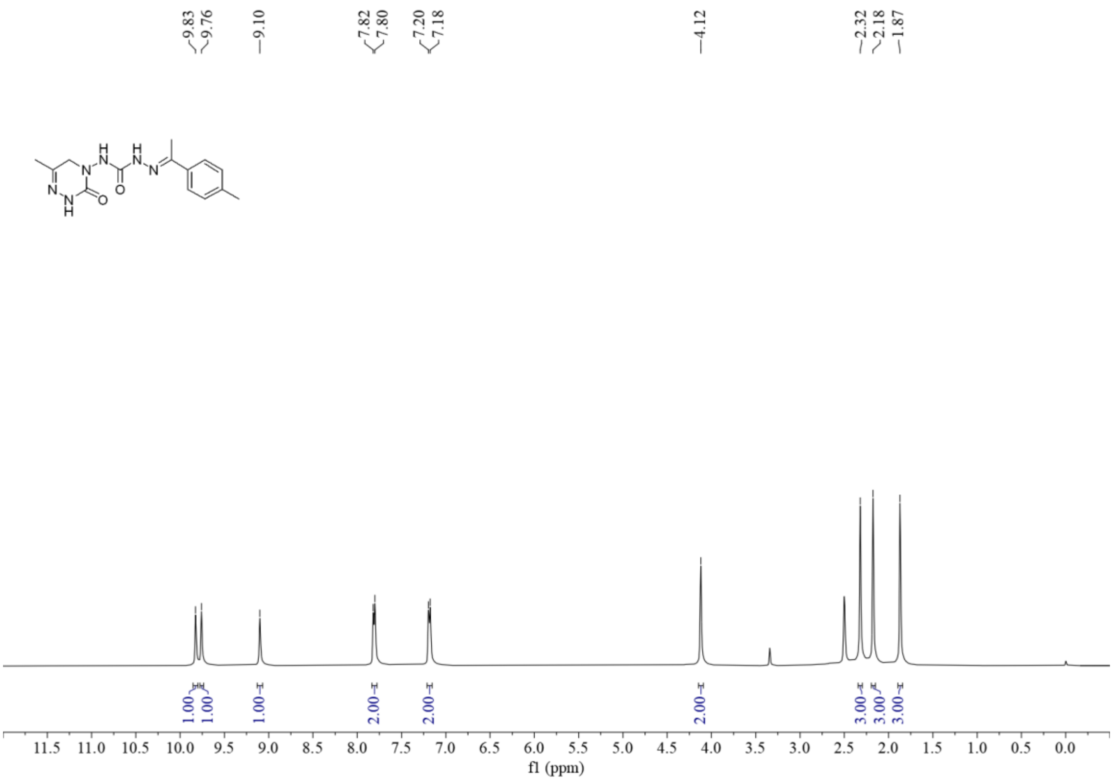

Fig. S59 ^{1}H NMR spectrum of **5g**

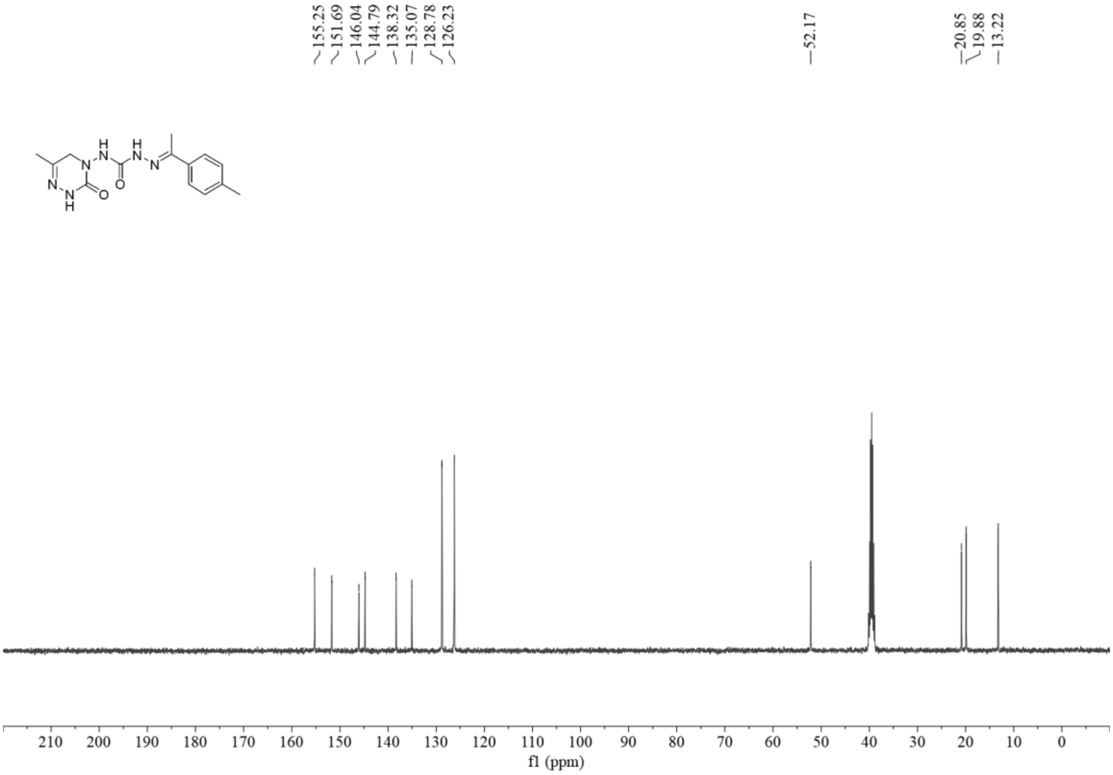

Fig. S60 ^{13}C NMR spectrum of **5g**

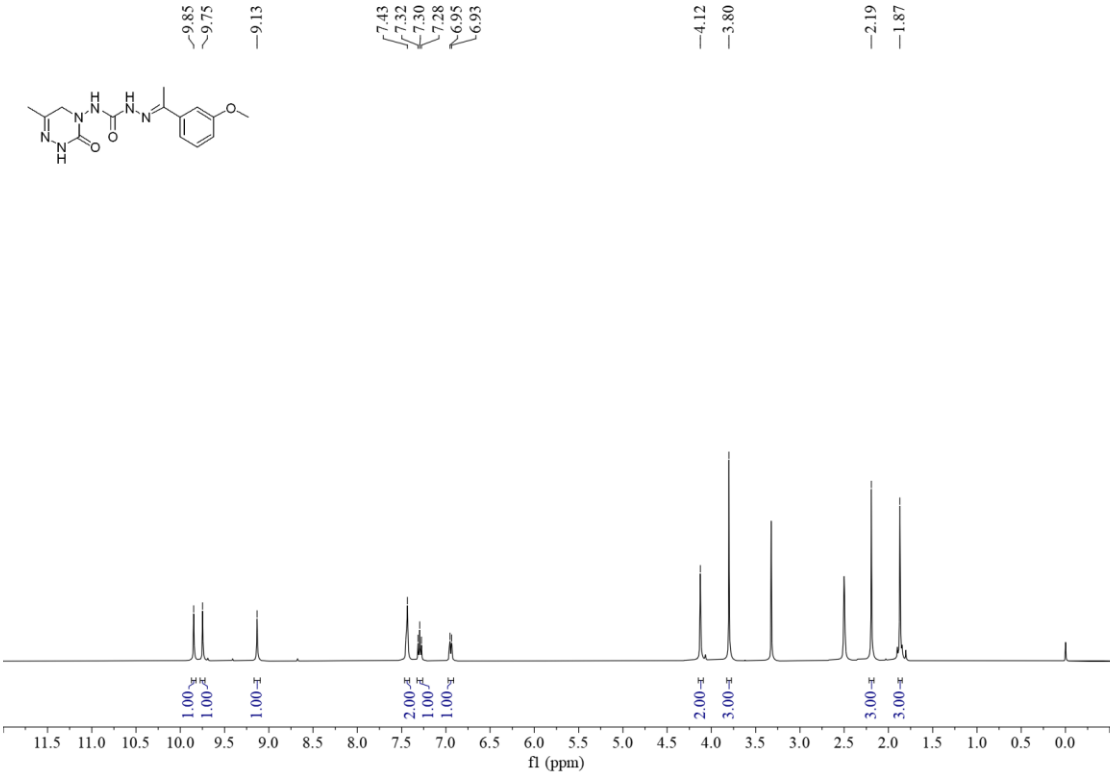

Fig. S61 ^{1}H NMR spectrum of **5h**

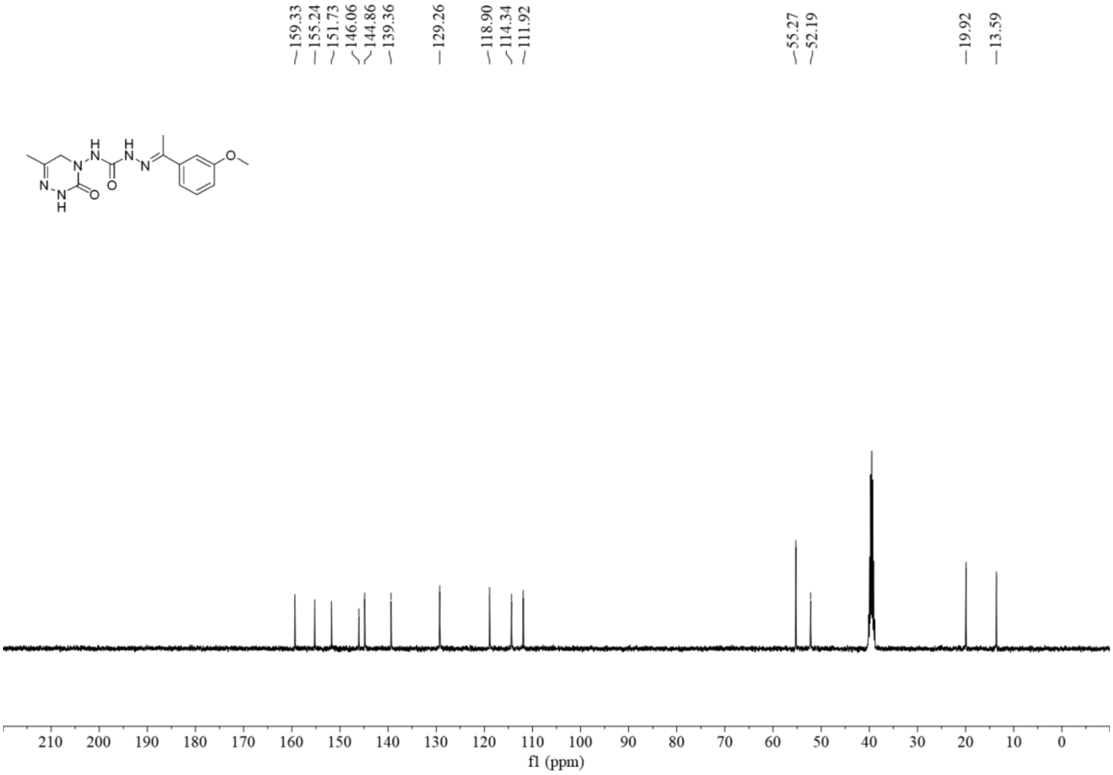

Fig. S62 ^{13}C NMR spectrum of **5h**

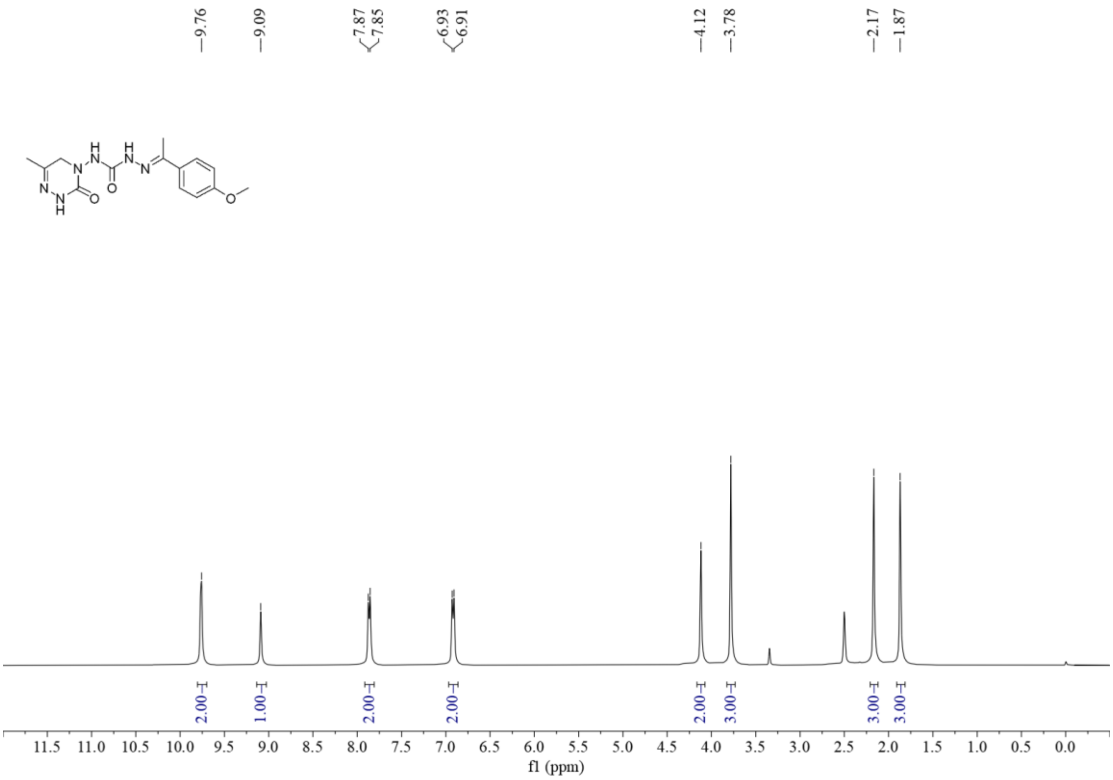

Fig. S63 ^{1}H NMR spectrum of **5i**

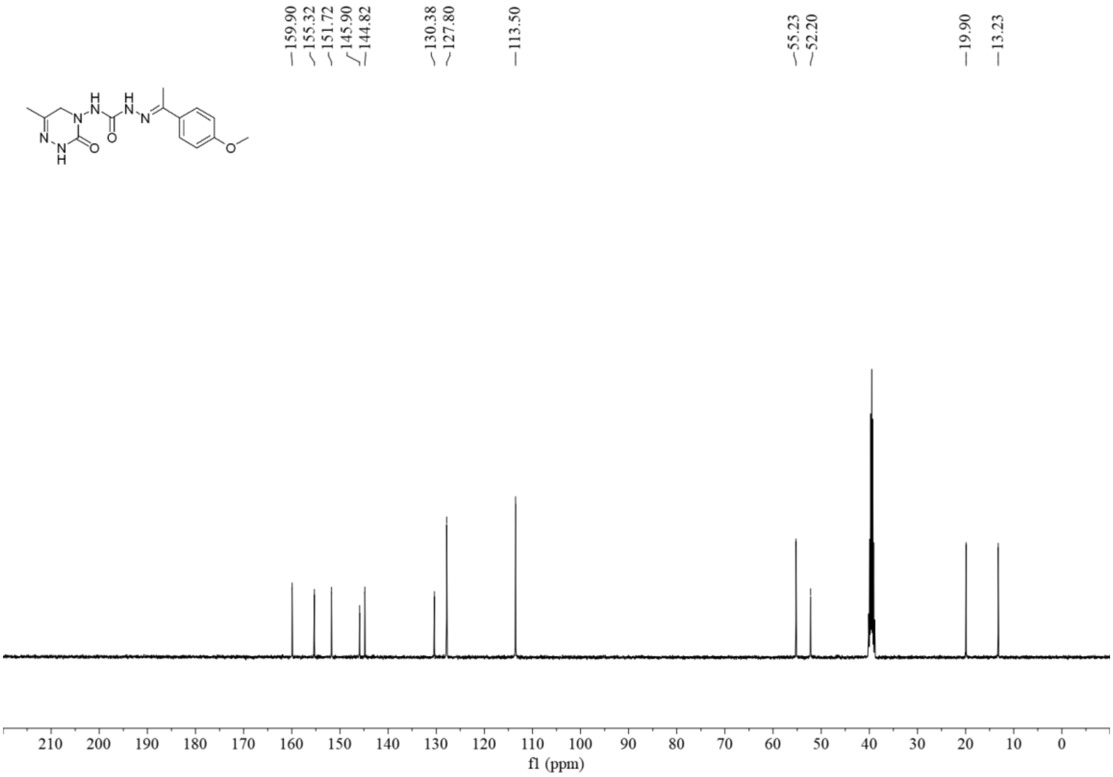

Fig. S64 ^{13}C NMR spectrum of **5i**

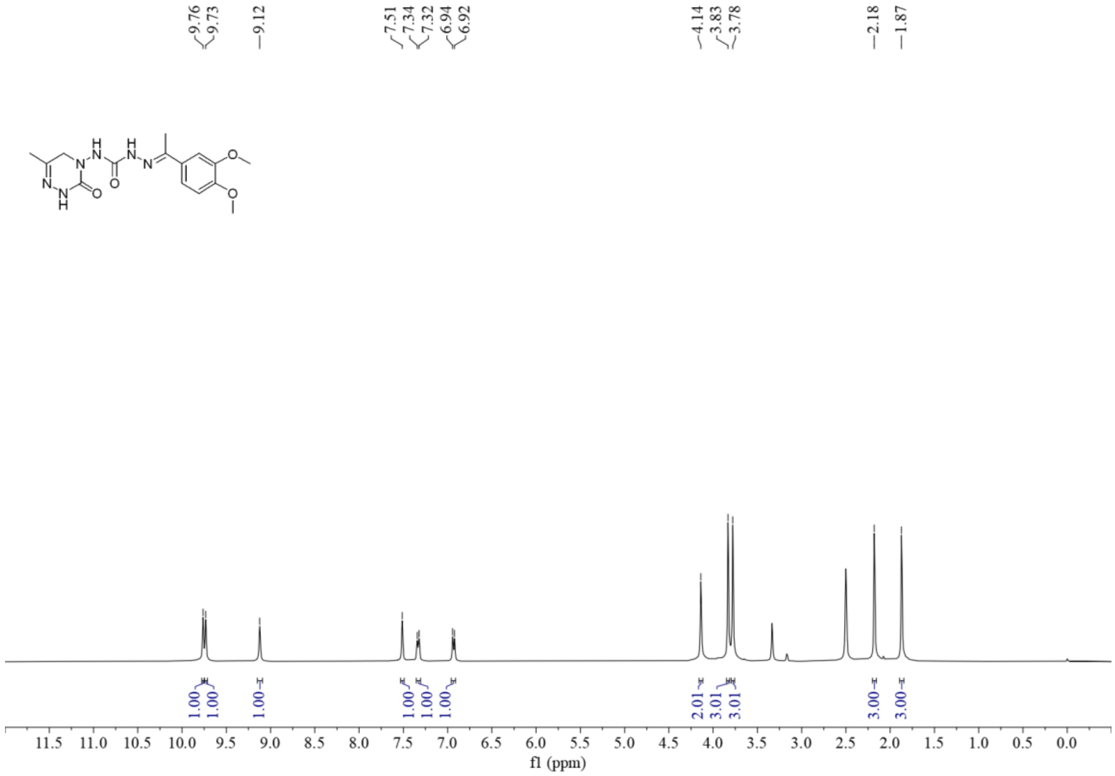

Fig. S65 ^{1}H NMR spectrum of **5j**

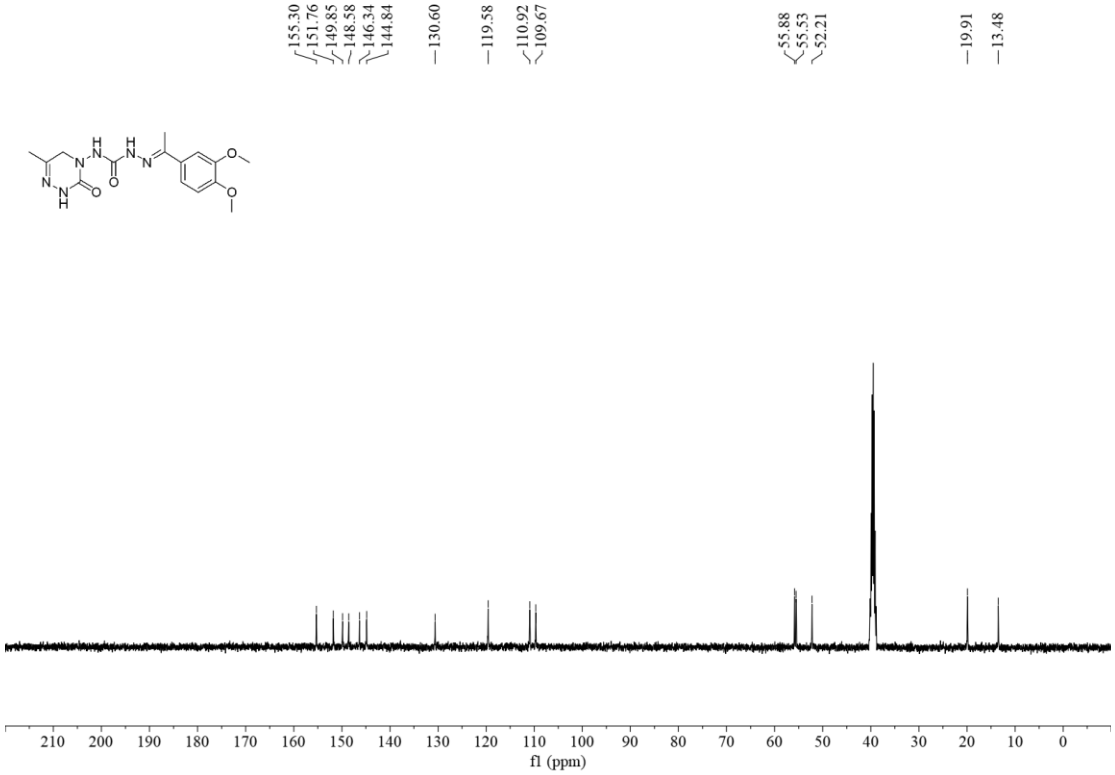

Fig. S66 ^{13}C NMR spectrum of **5j**

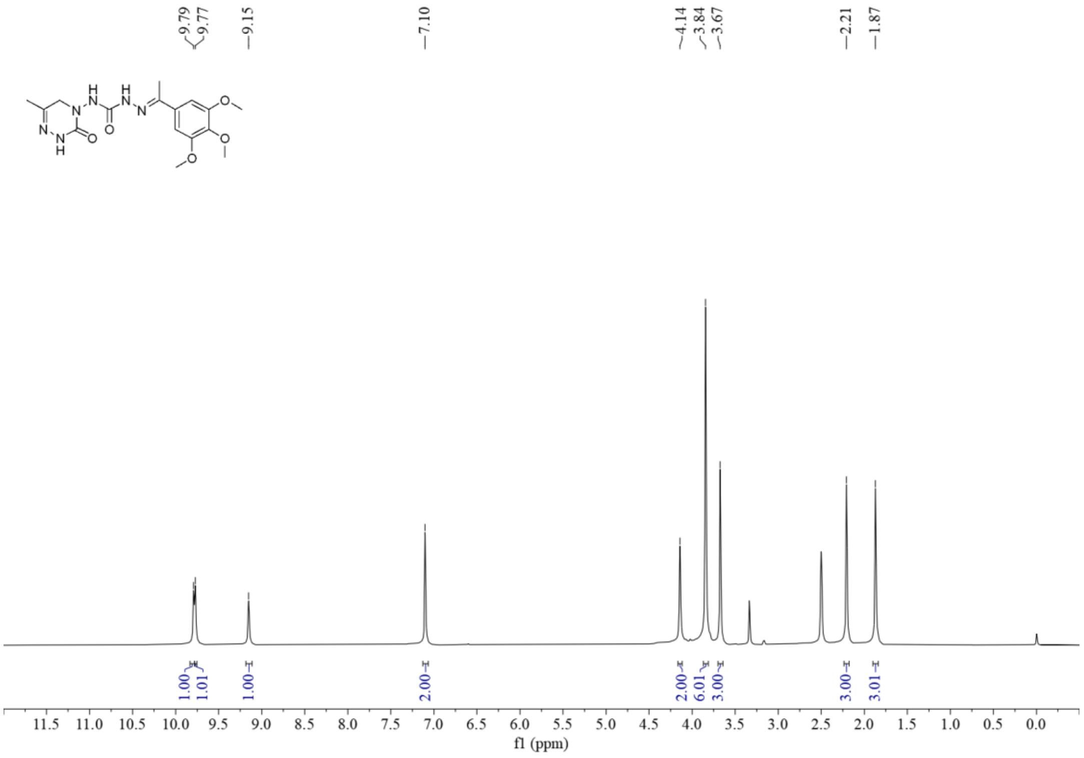

Fig. S67 ^{1}H NMR spectrum of **5k**

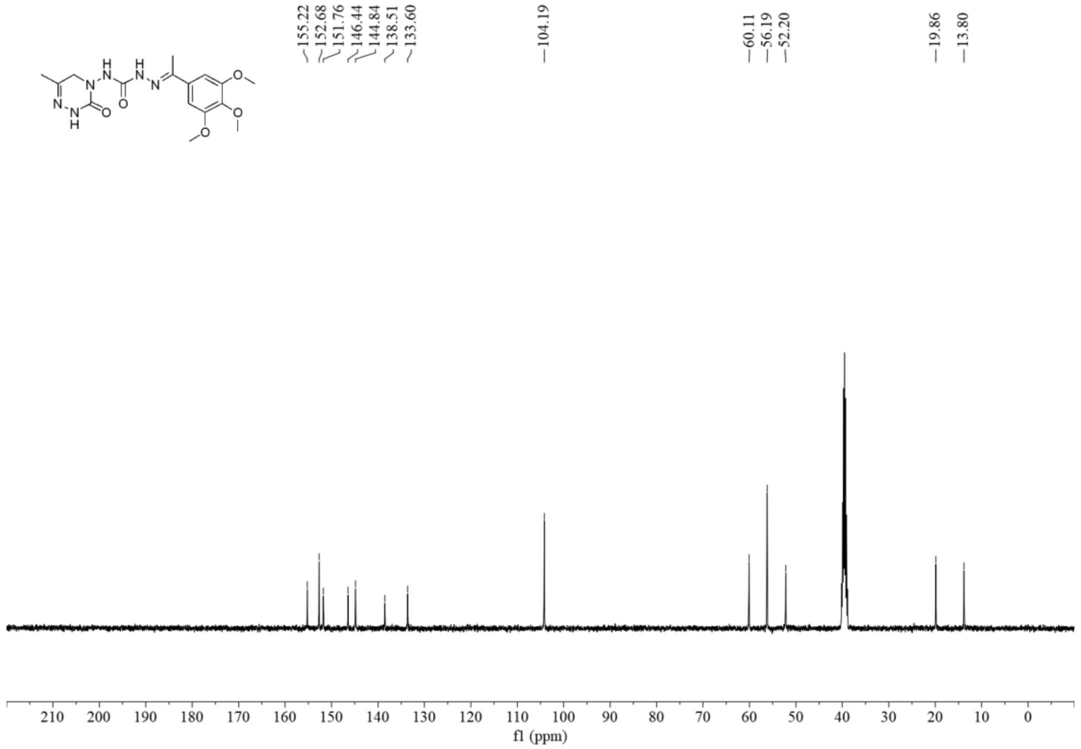

Fig. S68 ^{13}C NMR spectrum of **5k**

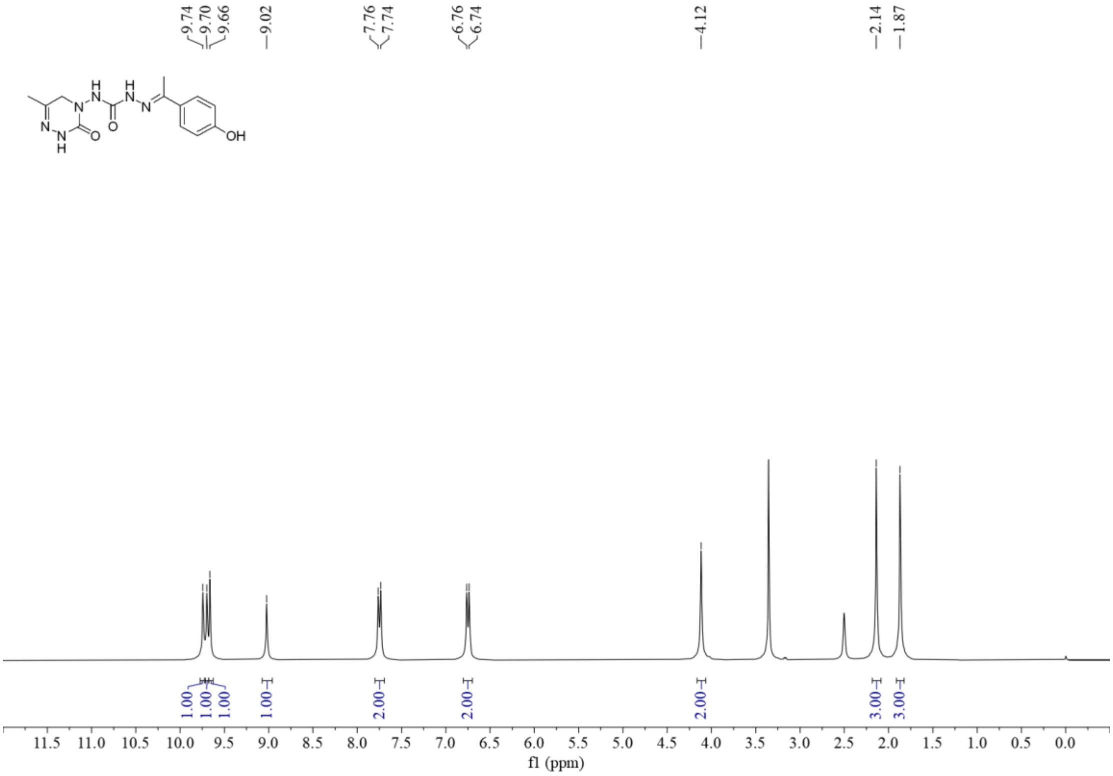

Fig. S69 ^{1}H NMR spectrum of **5l**

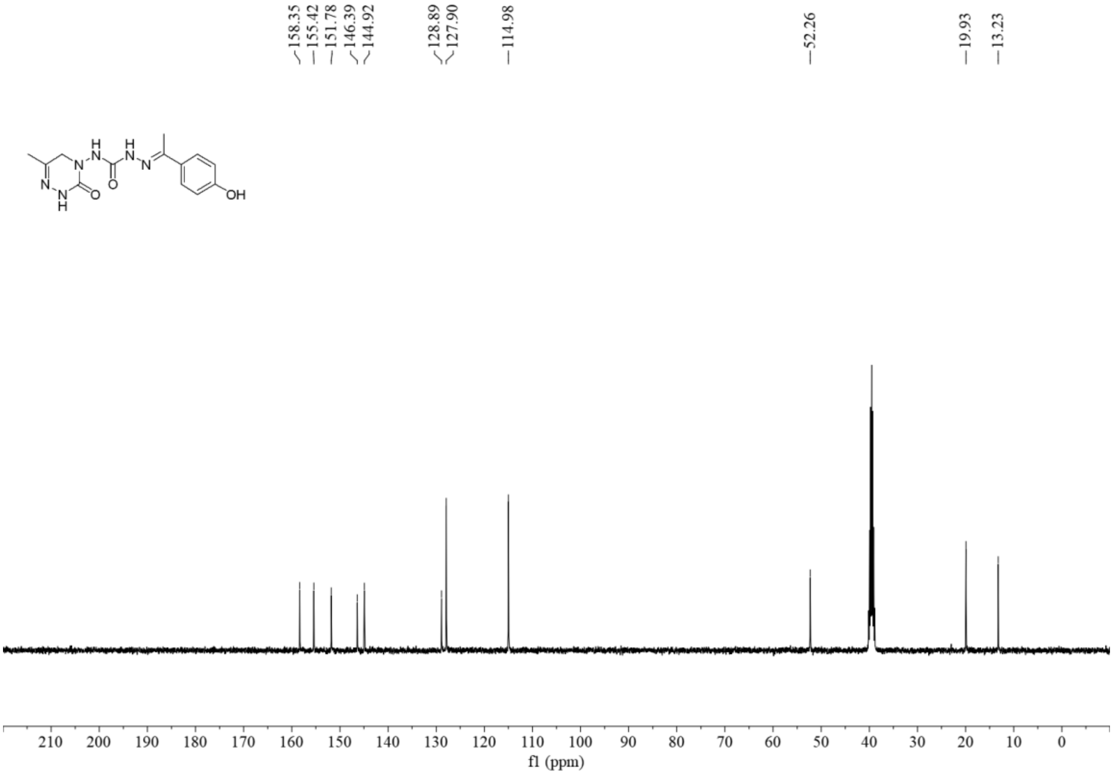

Fig. S70 ^{13}C NMR spectrum of **5l**

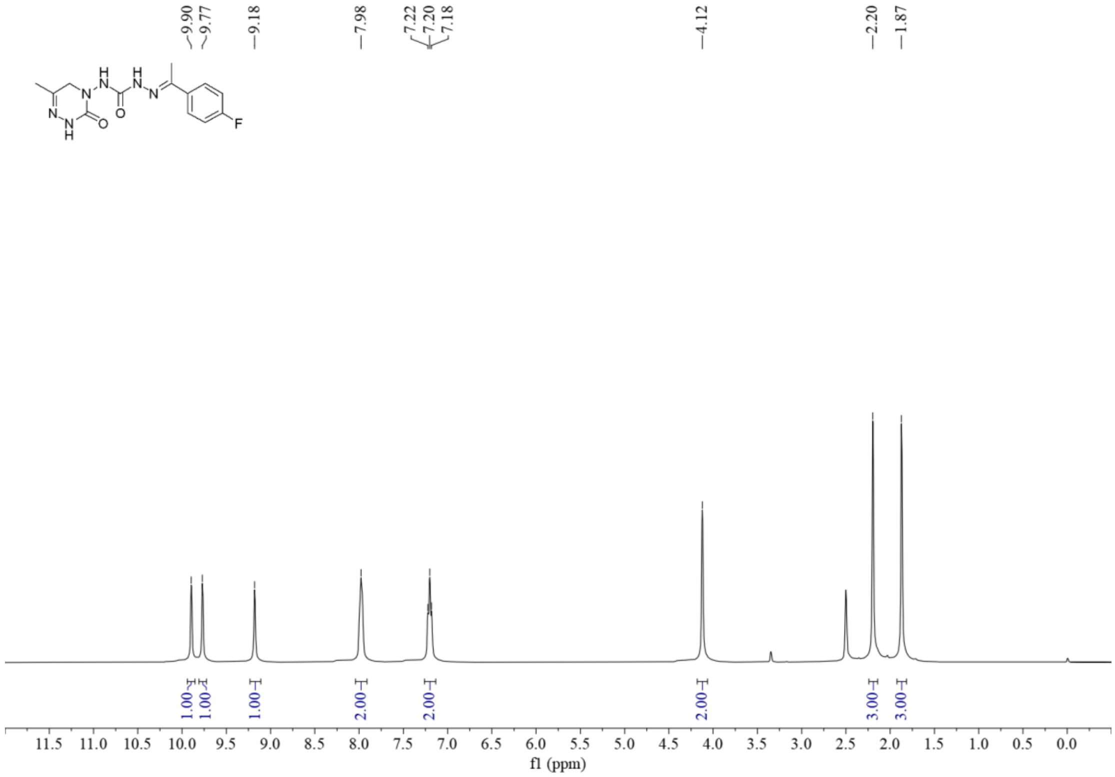

Fig. S71 ^{1}H NMR spectrum of **5m**

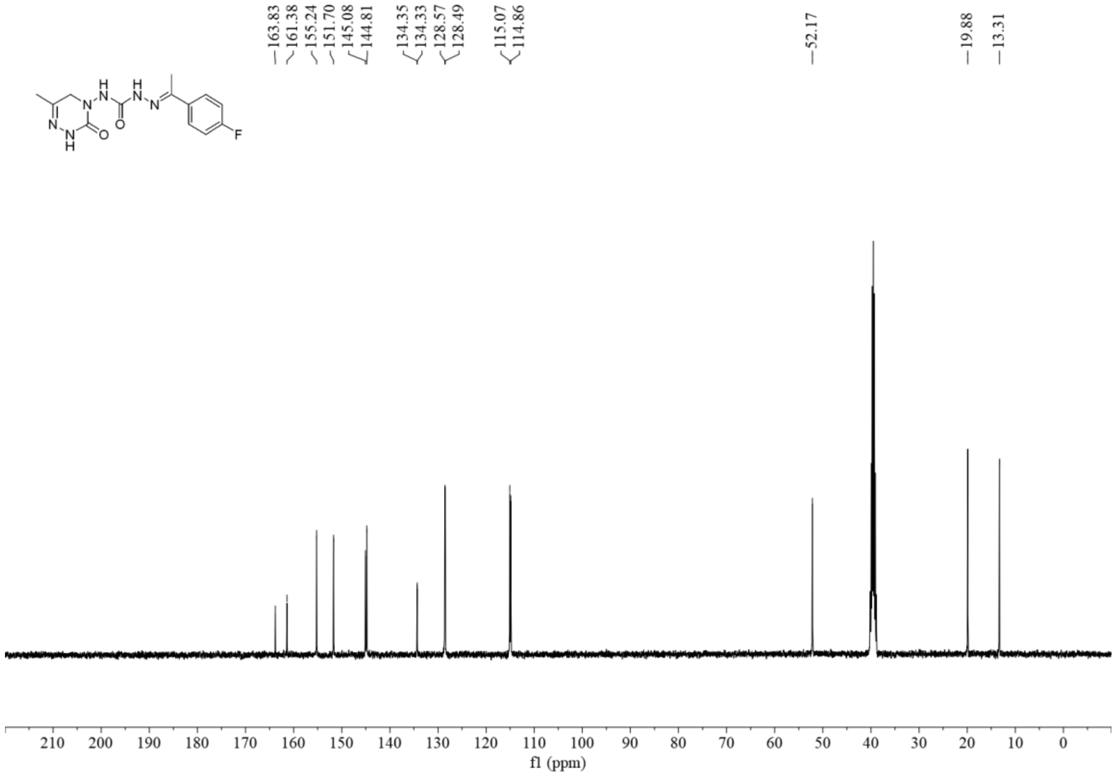

Fig. S72 ^{13}C NMR spectrum of **5m**

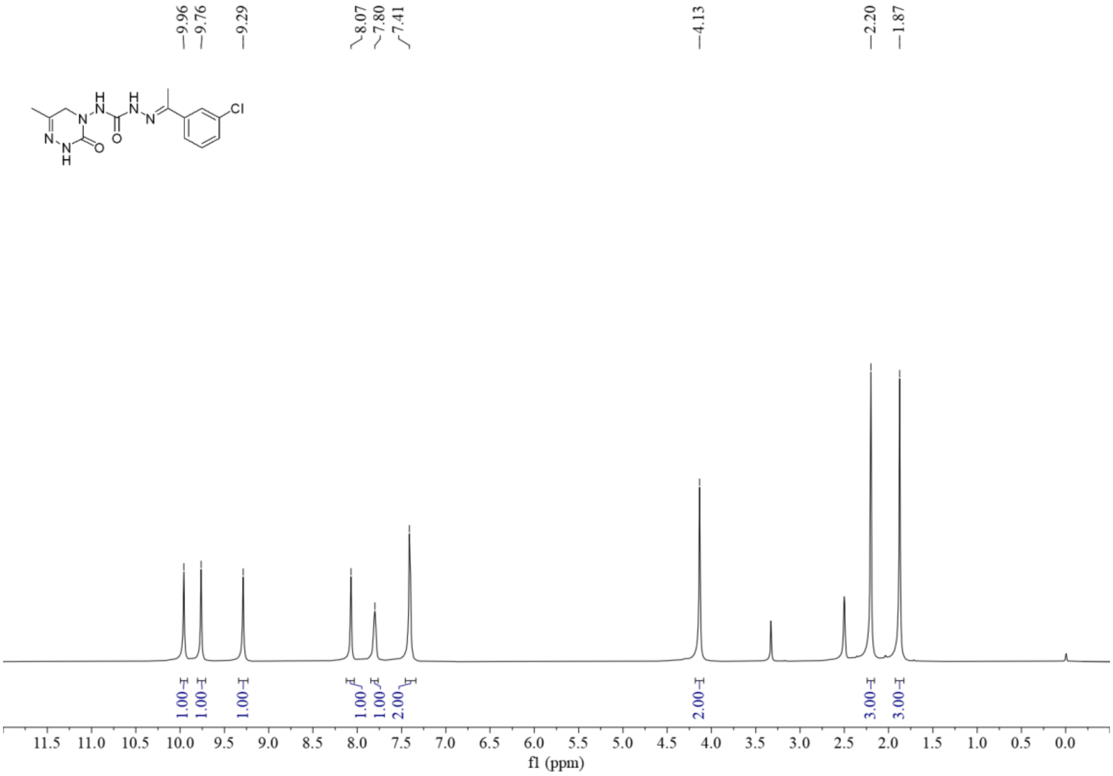

Fig. S73 ^{1}H NMR spectrum of **5n**

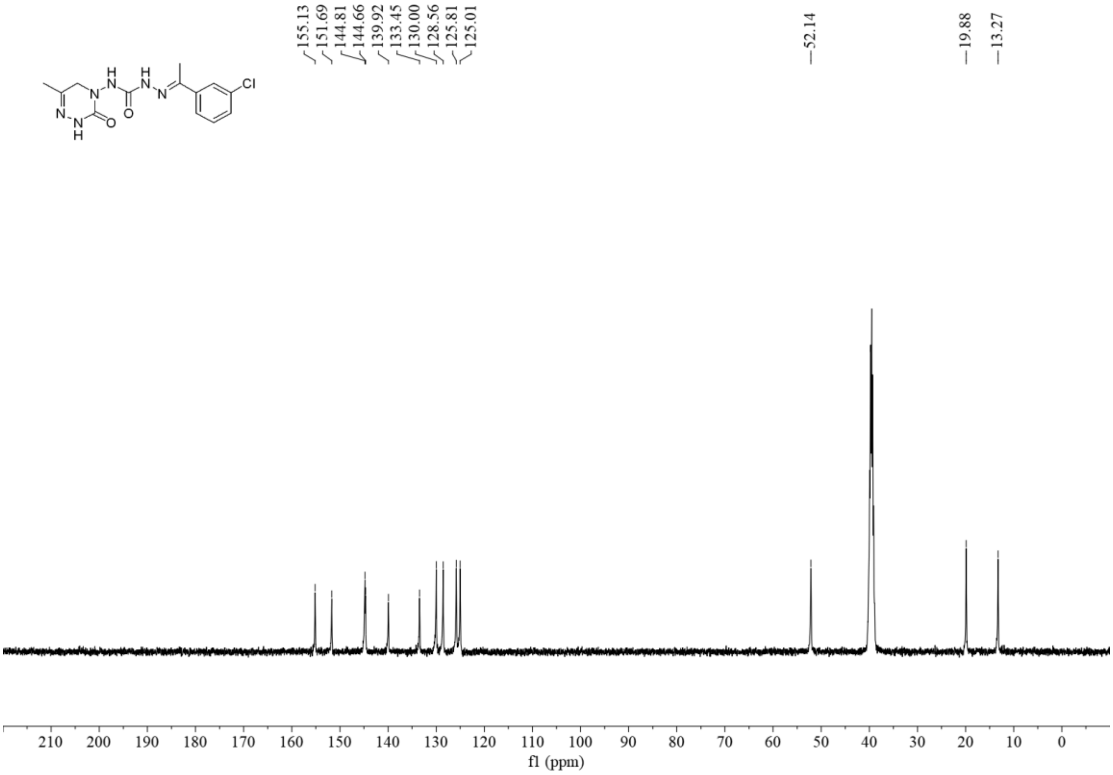

Fig. S74 ^{13}C NMR spectrum of **5n**

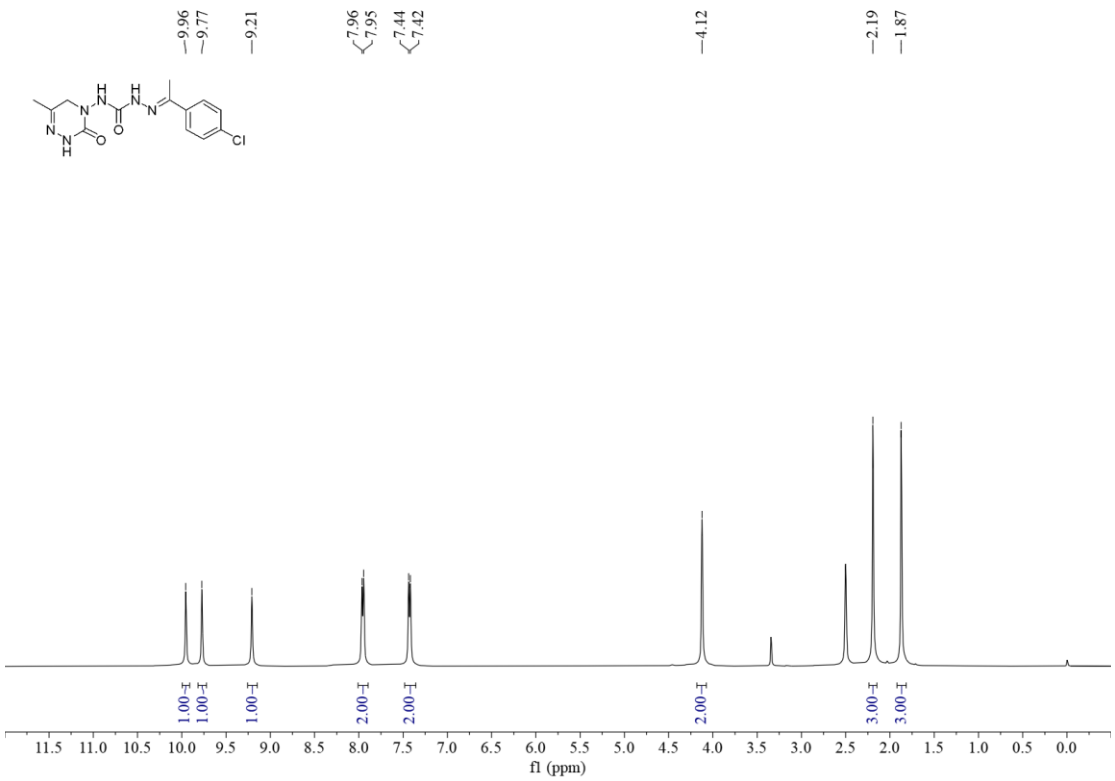

Fig. S75 ^{1}H NMR spectrum of **5o**

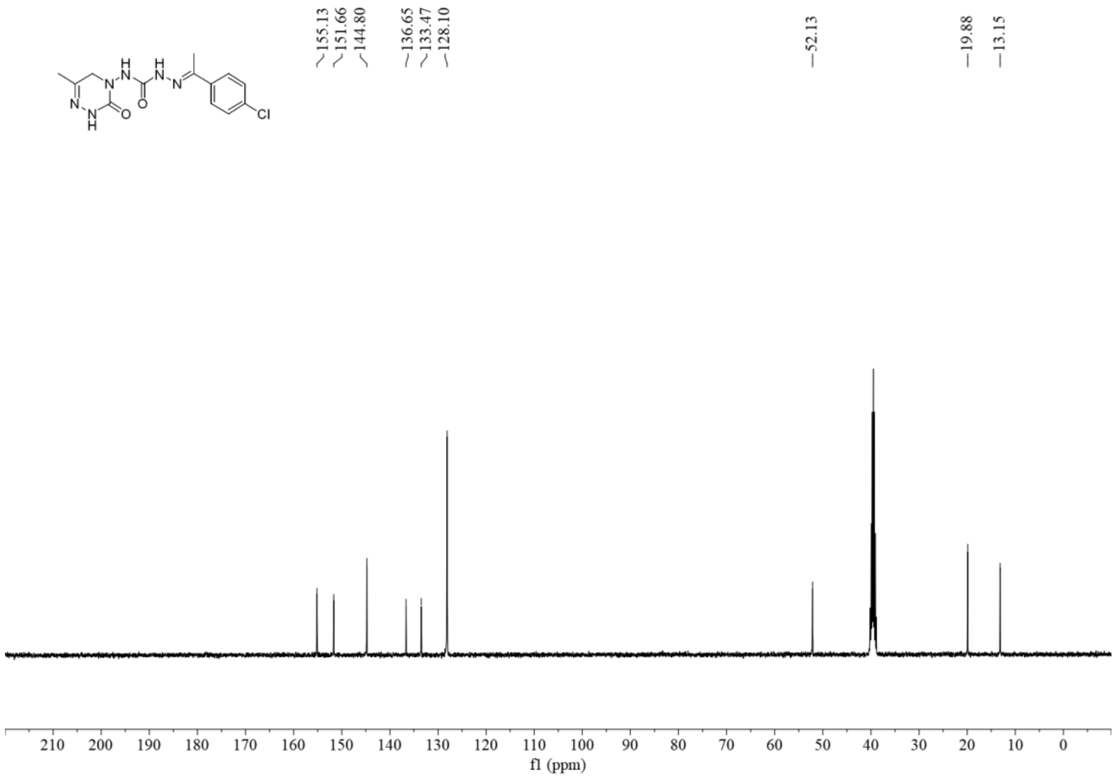

Fig. S76 ^{13}C NMR spectrum of **5o**

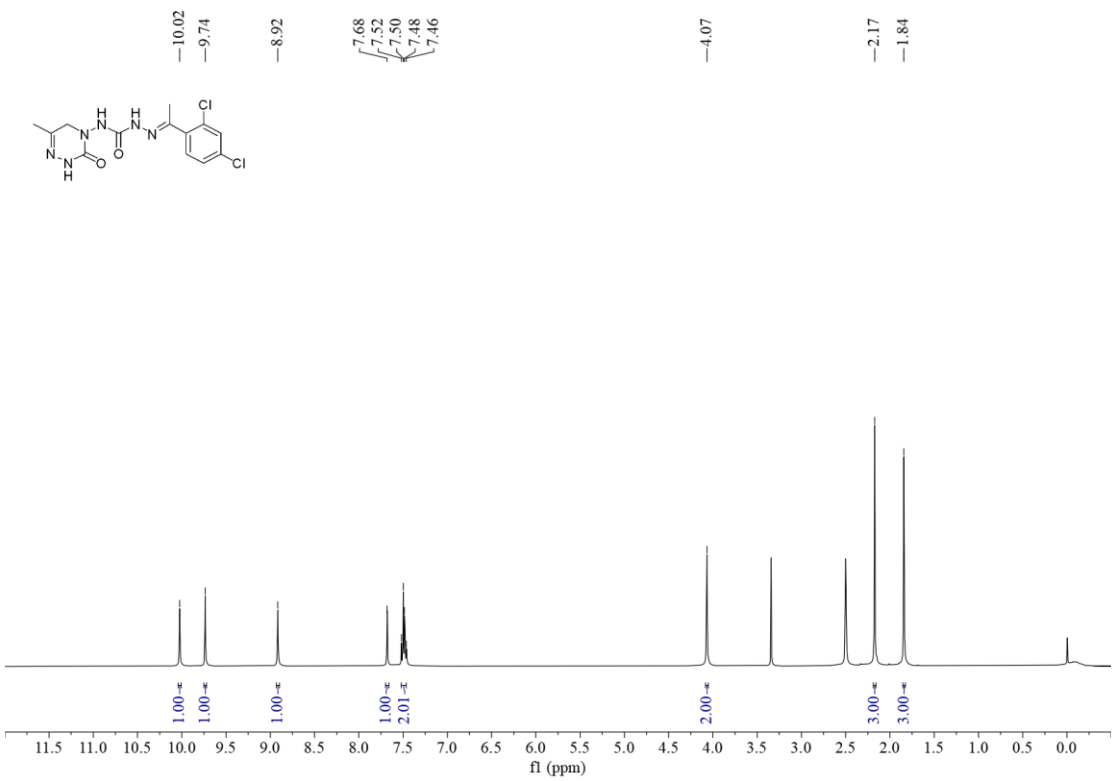

Fig. S77 ^{1}H NMR spectrum of **5p**

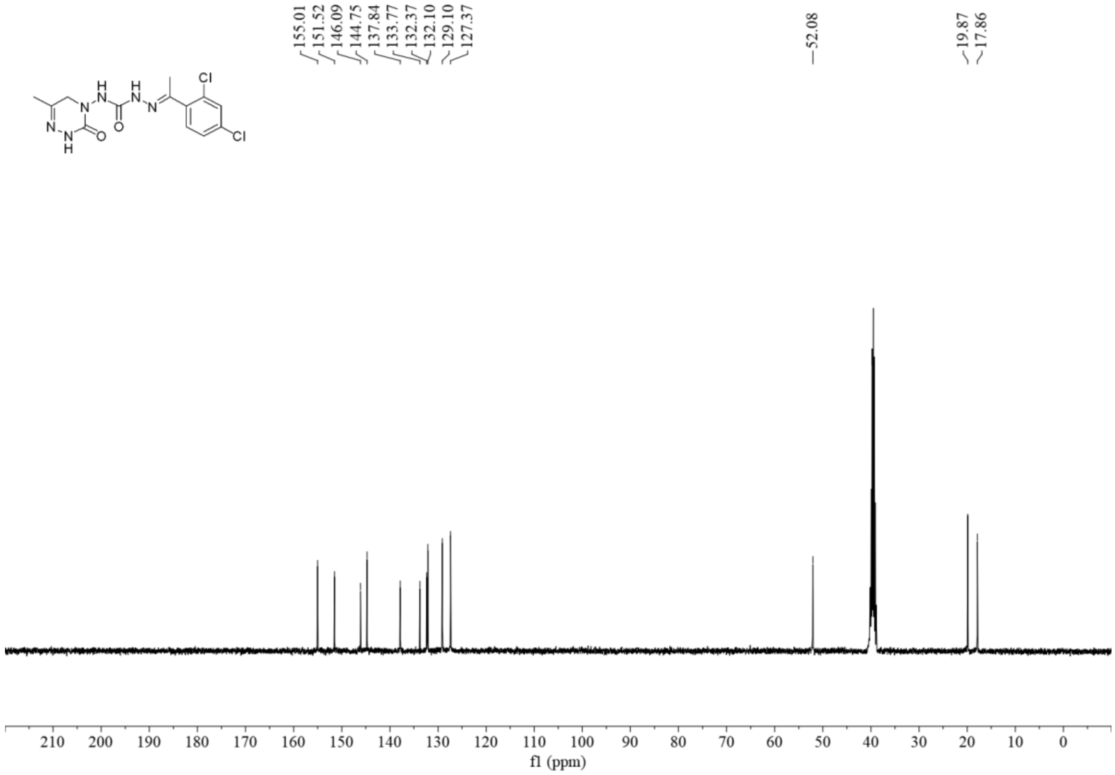

Fig. S78 ^{13}C NMR spectrum of **5p**

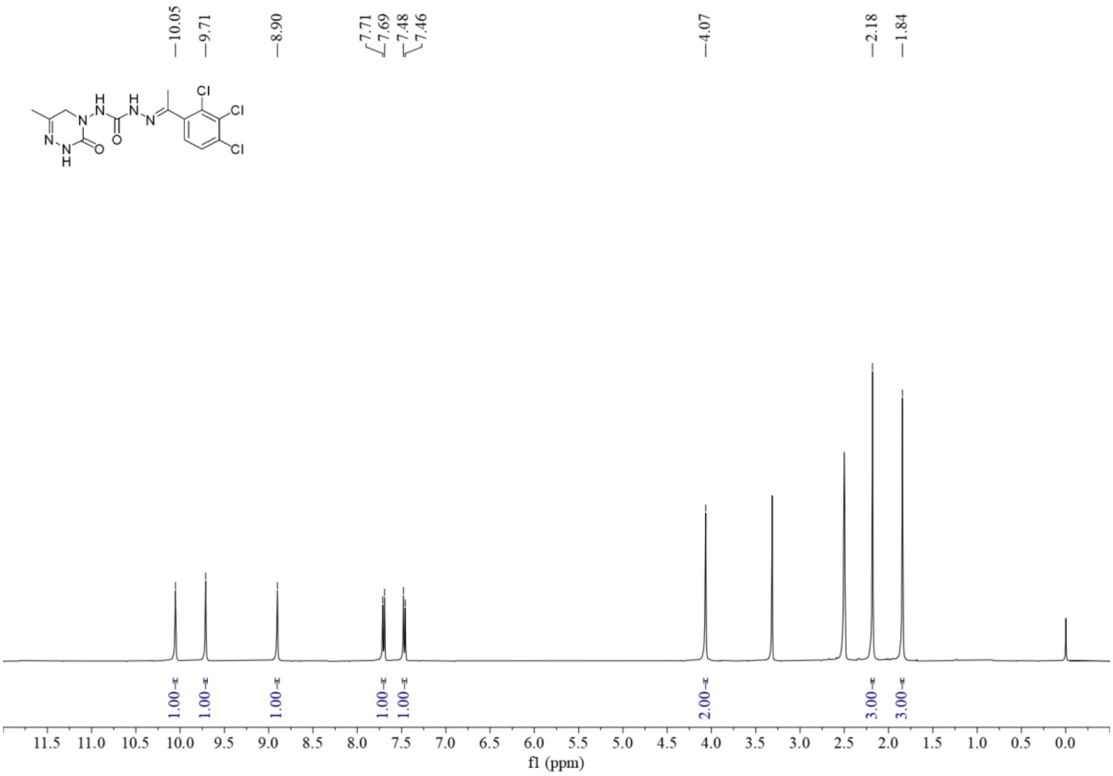

Fig. S79 ^{1}H NMR spectrum of **5q**

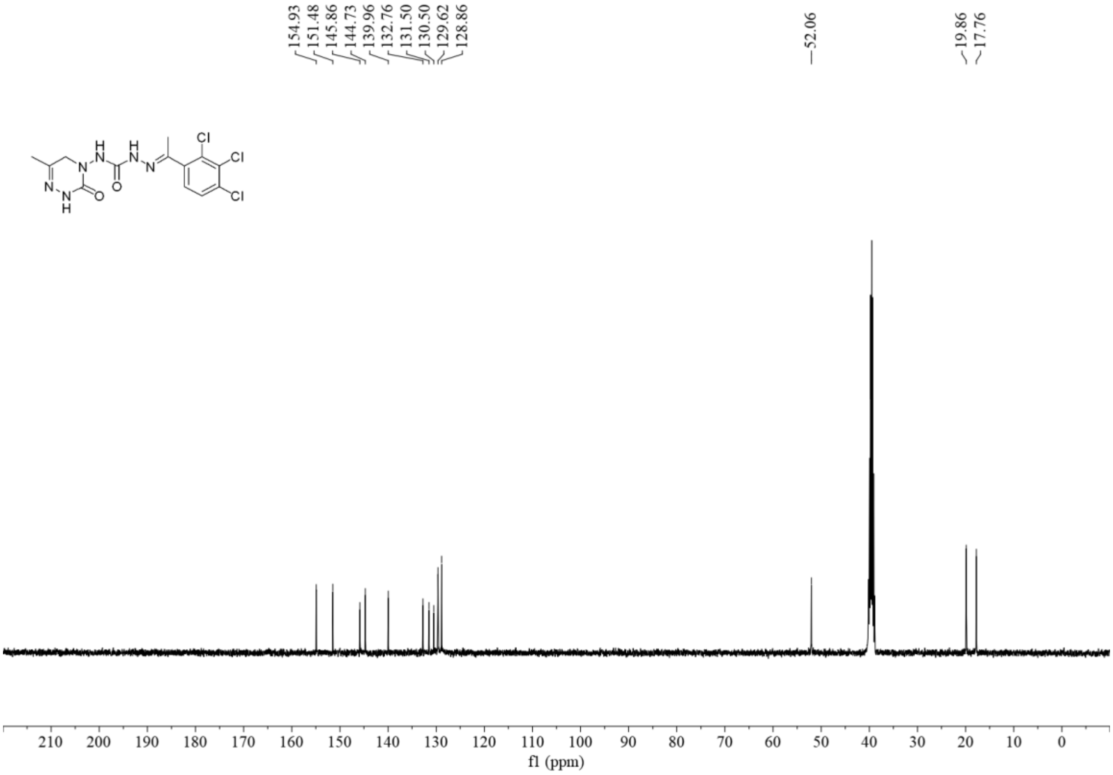

Fig. S80 ^{13}C NMR spectrum of **5q**

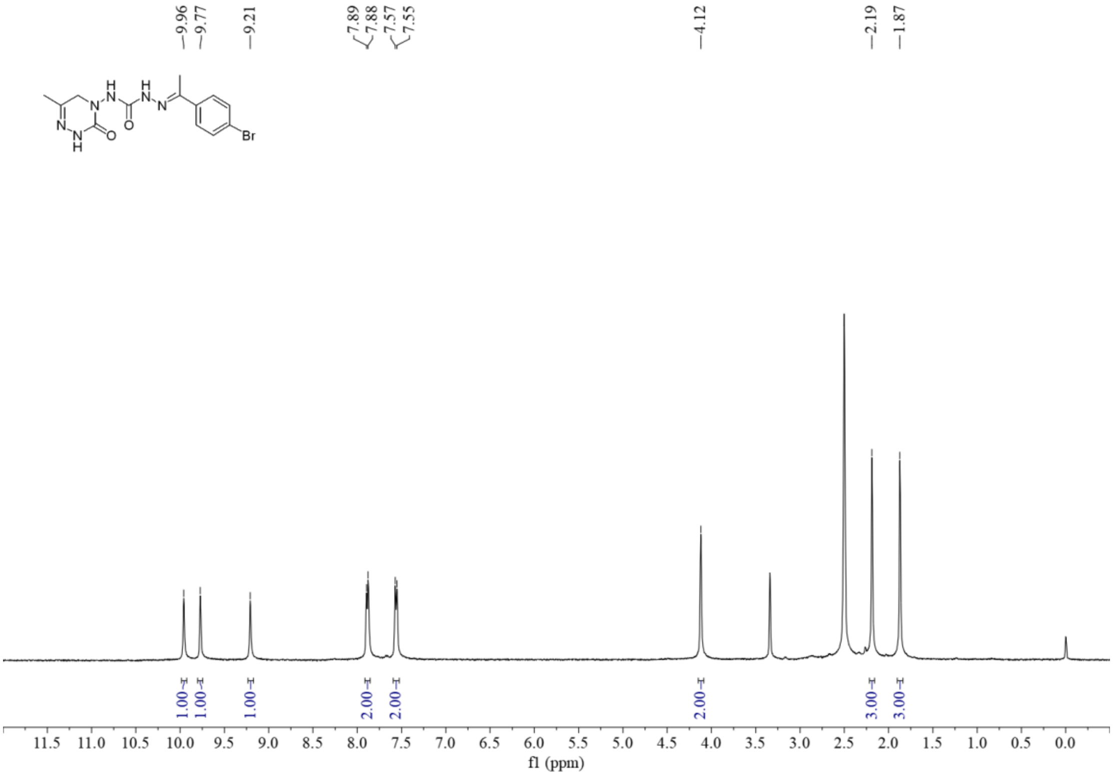

Fig. S81 ^{1}H NMR spectrum of **5r**

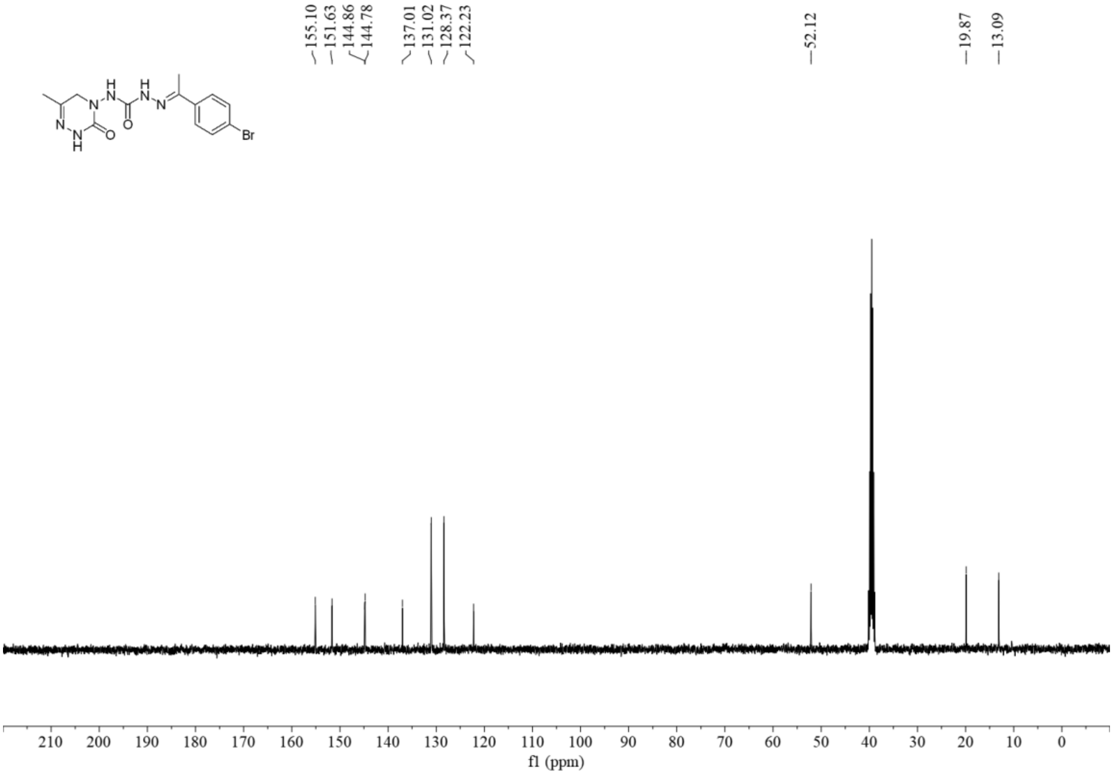

Fig. S82 ^{13}C NMR spectrum of **5r**

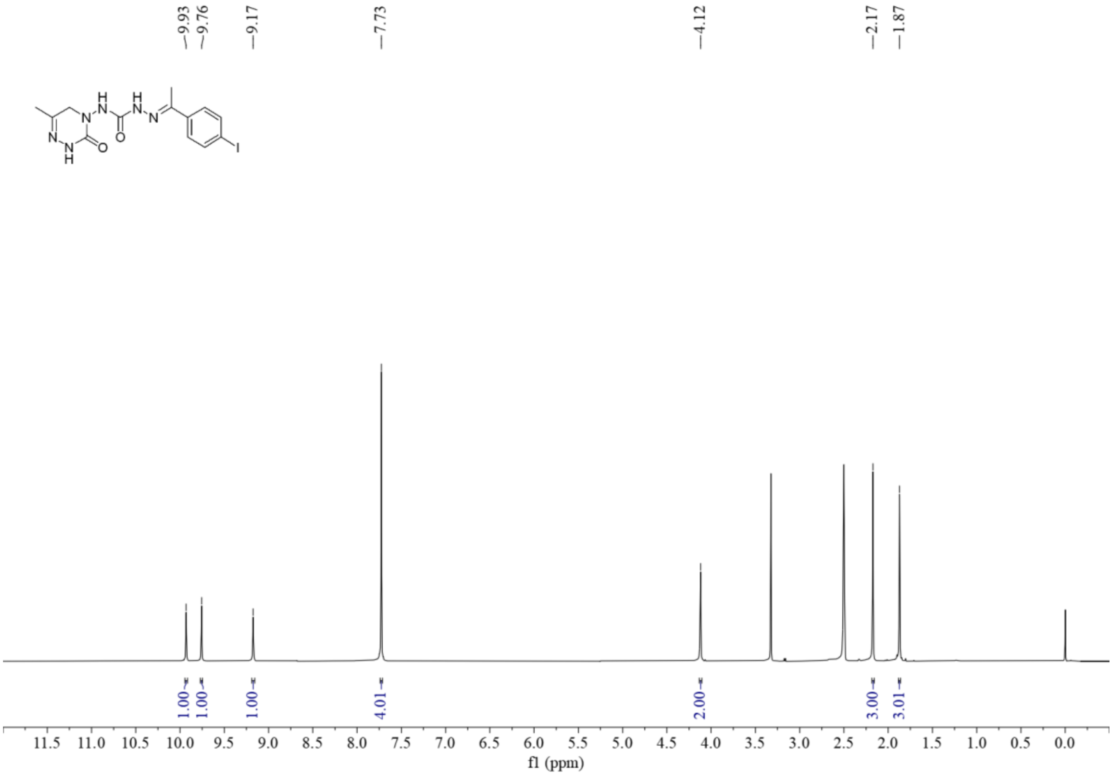

Fig. S83 ^{1}H NMR spectrum of **5s**

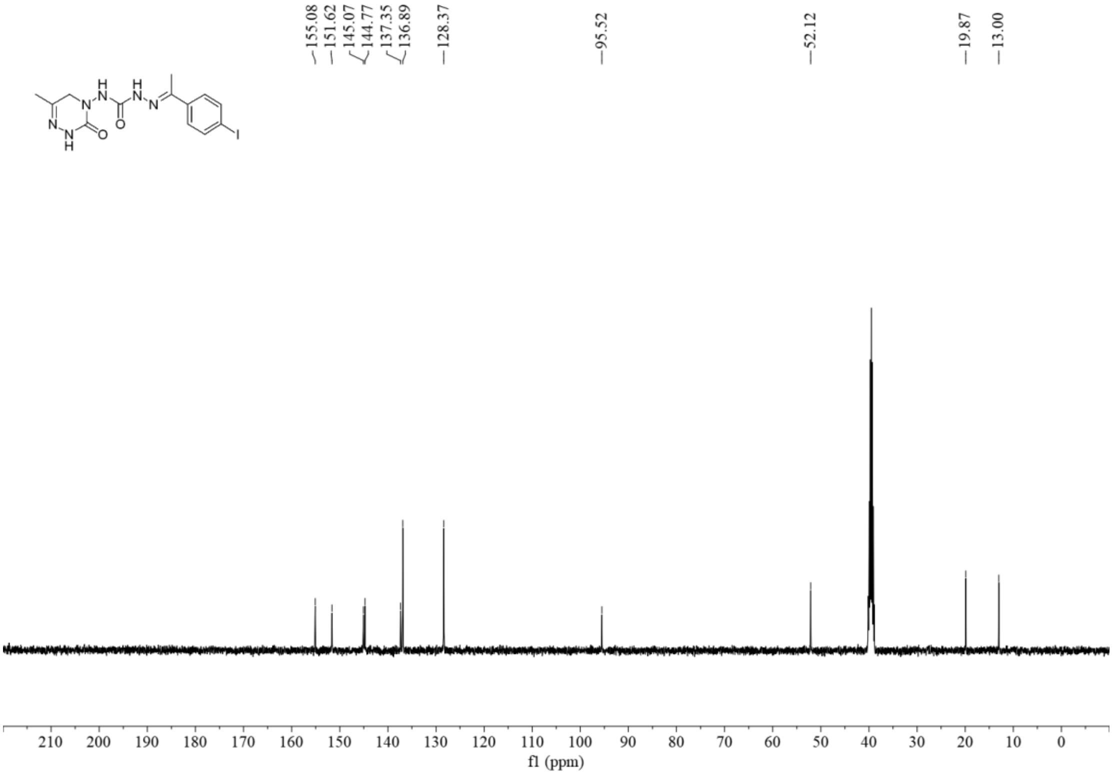

Fig. S84 ^{13}C NMR spectrum of **5s**

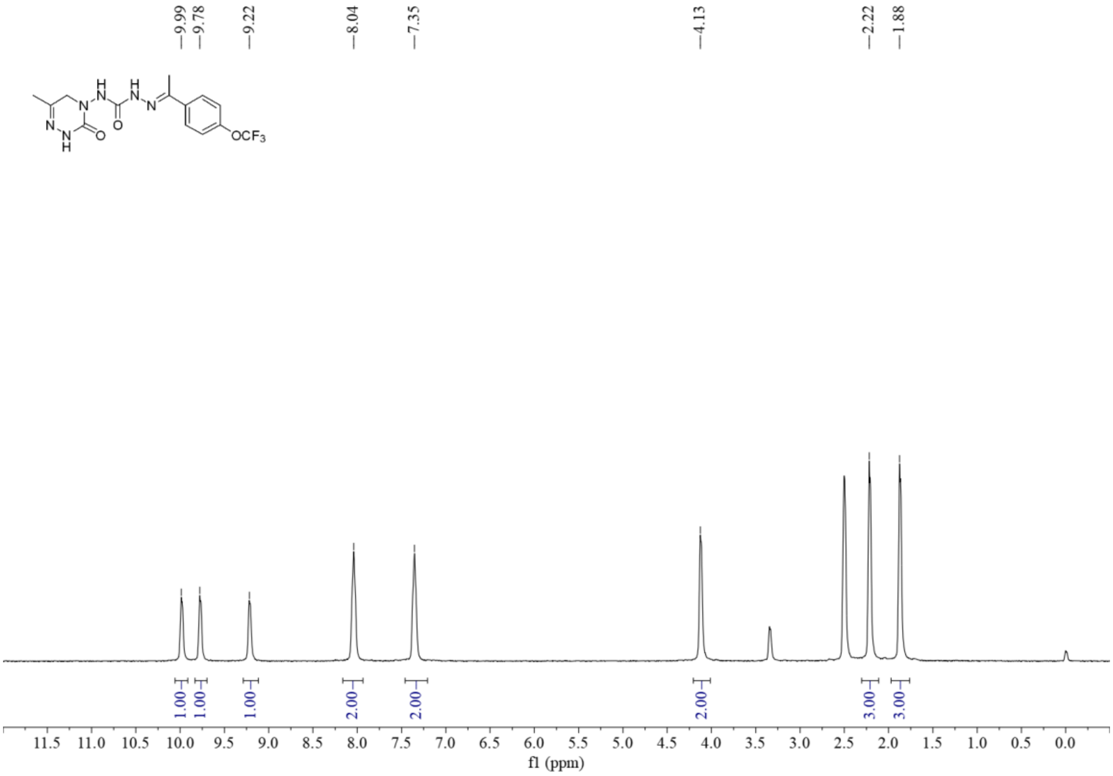

Fig. S85 ^{1}H NMR spectrum of **5t**

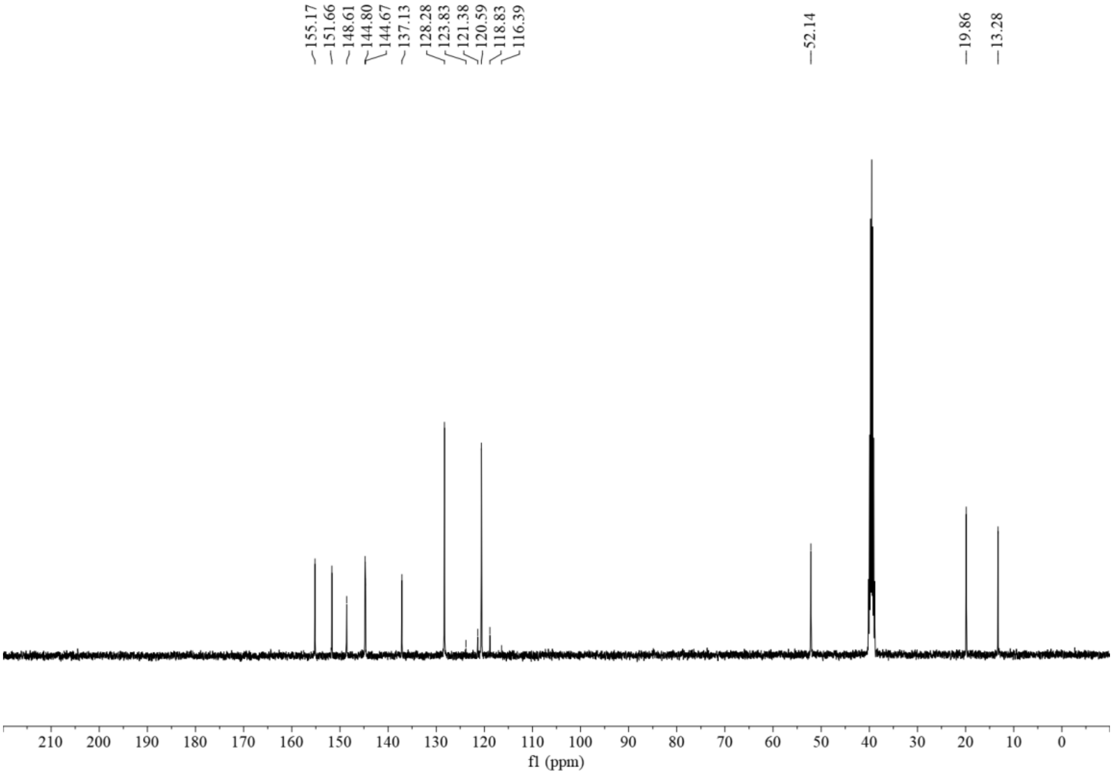

Fig. S86 ^{13}C NMR spectrum of **5t**

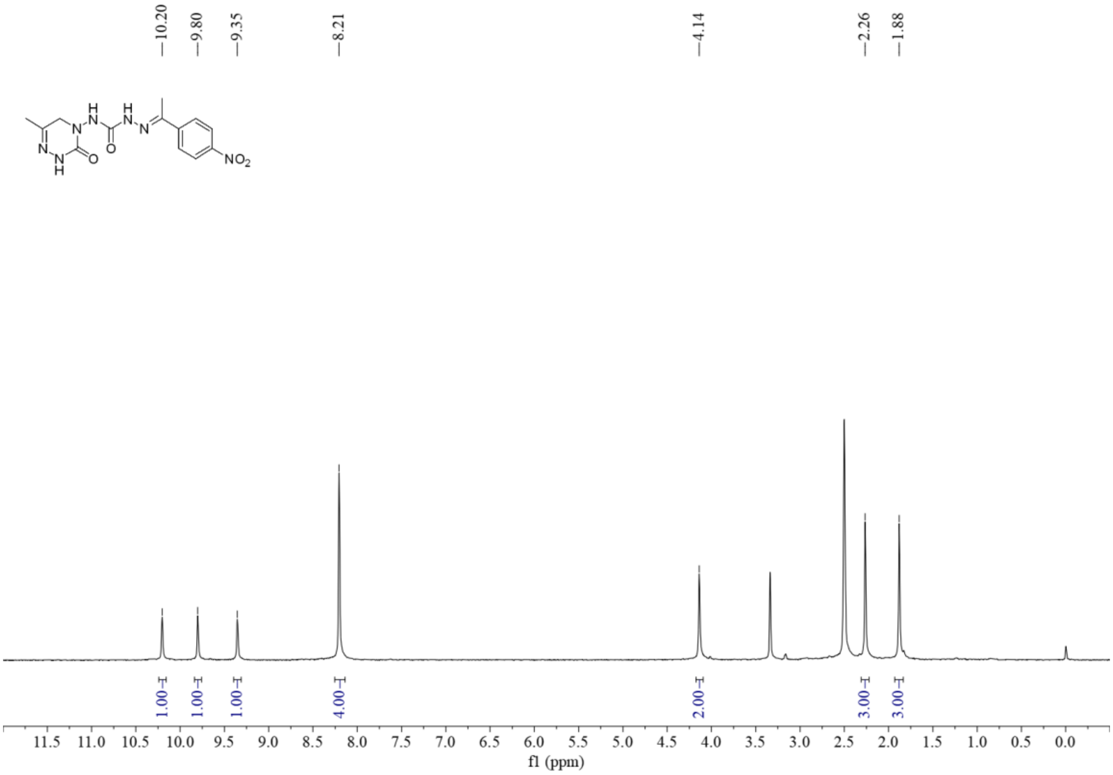

Fig. S87 ^{1}H NMR spectrum of **5u**

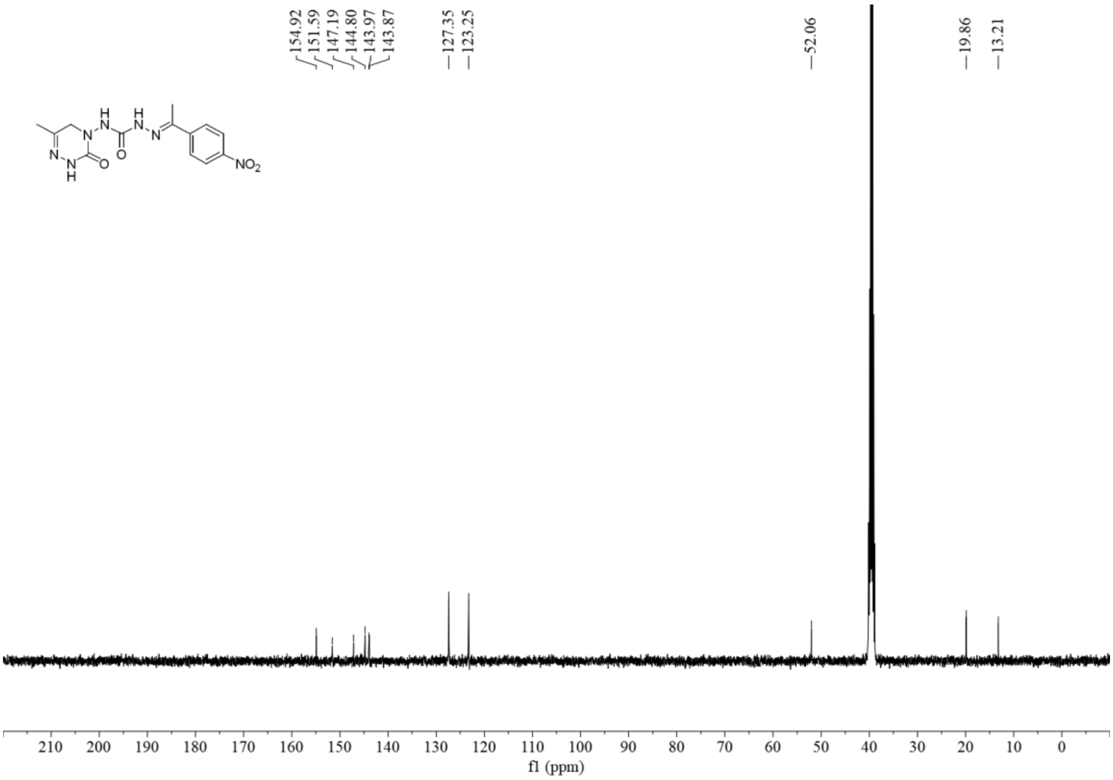

Fig. S88 ^{13}C NMR spectrum of **5u**

Biological Assay

All bioassays were performed on representative test organisms reared in the laboratory. The bioassay was repeated in triplicate at 25 ± 1 °C. Assessments were made on a dead/alive basis, and mortality rates were corrected using Abbott's formula. Evaluations were based on a percentage scale of 0−100, where 0 equals no activity and 100 equals total kill. For comparative purpose, pymetrozine was tested under the same conditions.

Detailed bioassay procedures for the insecticidal activities.

Insecticidal activities against *Aphis craccivora*: The insecticidal activities of compounds **3a-3w**, **5a-5u** and the pymetrozine were tested against A. craccivora by foliar application. About 60 aphids were transferred to the shoot with 3−5 fresh leaves of horsebean. The shoot with aphids was cut and dipped into the test solution for 2 s, after removing extra solutions on the leaf; the aphids were raised in the shoot at 25±1℃ and 85% relative humidity for 96 h. Each experiment for one compound was triplicated. The revised death rate was calculated by Abbott's formula.

Larvicidal Activities against cotton bollworm (*Helicoverpa armigera*), corn borer (*Ostrinia nubilalis*) and oriental armyworm (*Mythimna separata*): Stock solutions of each test compound was prepared in dimethylformamide at a concentration of 600 mg/L. Leaf-dip method was used. Leaf discs (5 cm × 3 cm) were cut from fresh cabbage leaves (or other leaves) and then dipped into the test solution for 3 s. After air-drying, the treated leaf discswere placed individually into vertical tube (or Petri dishes) and the discs were infested with 10 larvae (for example: 10 second-instar diamondback moth larvae, 10 fourth-instar orientalarmyworm larvae). Percentage mortalities were evaluated 3 days

after treatment. Evaluations were based on a percentage scale of 0 - 100, where 0 equals no activity and 100 equals total kill. Each treatment was repeated three times.

Detailed bioassay procedures for the fungicidal activities. The compounds were evaluated in mycelial growth tests in artificial media against 14 plantpathogens at rate of 50 mg/L. Test compound was dissolved in a suitable amount of acetone and diluted with water containing 0.1 % TW - 80 to the concentration of 500 mg/L. To each petri dish was added 1 mL such solution and 9 mL culture medium to make a 50 mg/L of medicated tablet, whereas to another petri dish was added 1 mL sterilized water and 9 mL culture medium as blank control. A diameter of 4 mm of hyphae was cut by a hole puncher along the hyphae for bacteria to the outerplate and moved to the medicated tablet. Each treatment was repeated three times. The dishes were stored in controlled environment cabinets (24 ± 1 °C) for 48 h, after which the diameter of mycelia growth was investigated and percentage inhibition was calculated.

Percentage inhibition (%) = (averaged diameter of mycelia in blank controls – averageddiameter of mycelia in medicated tablets) / averaged diameter of mycelia in blank controls
